# Collaborative study from the Bladder Cancer Advocacy Network for the genomic analysis of metastatic urothelial cancer

**DOI:** 10.1038/s41467-022-33980-9

**Published:** 2022-11-04

**Authors:** Jeffrey S. Damrauer, Wolfgang Beckabir, Jeff Klomp, Mi Zhou, Elizabeth R. Plimack, Matthew D. Galsky, Petros Grivas, Noah M. Hahn, Peter H. O’Donnell, Gopa Iyer, David I. Quinn, Benjamin G. Vincent, Diane Zipursky Quale, Sara E. Wobker, Katherine A. Hoadley, William Y. Kim, Matthew I. Milowsky

**Affiliations:** 1 Lineberger Comprehensive Cancer Center, University of North Carolina, Chapel Hill, NC USA; 2 Department of Microbiology and Immunology, University of North Carolina, Chapel Hill, NC USA; 3 Department of Pharmacology, University of North Carolina, Chapel Hill, NC USA; 4 Department of Hematology and Oncology, Fox Chase Cancer Center, Temple Health, Philadelphia, PA USA; 5 Division of Hematology and Medical Oncology, Tisch Cancer Institute, Icahn School of Medicine at Mount Sinai, New York, NY USA; 6 Department of Medicine, Division of Medical Oncology, University of Washington, Seattle, USA; 7 Clinical Research Division, Fred Hutchinson Cancer Center, Seattle, USA; 8 Johns Hopkins University School of Medicine, Baltimore, MD USA; 9 Section of Hematology/Oncology, Department of Medicine, University of Chicago, Chicago, IL USA; 10 Memorial Sloan Kettering Cancer Center, New York, NY USA; 11 University of Southern California Norris Comprehensive Cancer Center, Los Angeles, CA USA; 12 Division of Hematology, University of North Carolina, Chapel Hill, NC USA; 13 Department of Medicine, University of North Carolina, Chapel Hill, NC USA; 14 Curriculum in Bioinformatics and Computational Biology, Computational Medicine Program, University of North Carolina, Chapel Hill, USA; 15 Bladder Cancer Advocacy Network, Bethesda, MD USA; 16 Department of Pathology and Laboratory Medicine, University of North Carolina, Chapel Hill, NC USA; 17 Department of Genetics, University of North Carolina, Chapel Hill, NC USA; 18 Division of Oncology, University of North Carolina, Chapel Hill, NC USA

**Keywords:** Bladder cancer, Cancer genomics, Bladder, Cancer immunotherapy, Metastasis

## Abstract

Urothelial Cancer - Genomic Analysis to Improve Patient Outcomes and Research (NCT02643043), UC-GENOME, is a genomic analysis and biospecimen repository study in 218 patients with metastatic urothelial carcinoma. Here we report on the primary outcome of the UC-GENOME—the proportion of subjects who received next generation sequencing (NGS) with treatment options—and present the initial genomic analyses and clinical correlates. 69.3% of subjects had potential treatment options, however only 5.0% received therapy based on NGS. We found an increased frequency of *TP53*^*E285K*^ mutations as compared to non-metastatic cohorts and identified features associated with benefit to chemotherapy and immune checkpoint inhibition, including: Ba/Sq and Stroma-rich subtypes, APOBEC mutational signature (SBS13), and inflamed tumor immune phenotype. Finally, we derive a computational model incorporating both genomic and clinical features predictive of immune checkpoint inhibitor response. Future work will utilize the biospecimens alongside these foundational analyses toward a better understanding of urothelial carcinoma biology.

## Abstract (web summary)

The Bladder Cancer Advocacy Network established the UC-GENOME study in order to create a biobank and data repository for metastatic urothelial carcinoma. Here, the authors present the first characterization and analysis of DNA and RNA sequencing data from the 218 patients included in the UC-GENOME.

## Introduction

Substantial progress has been made in understanding the molecular landscape of urothelial carcinoma (UC) over the past several years. Since The Cancer Genome Atlas’ (TCGA) first molecular characterization of muscle-invasive bladder cancers in 2014^1^, additional efforts have identified specific subtypes associated with patient outcomes and response to treatments^2–9^. At present, in spite of these efforts, there is only one targeted agent that has been FDA approved for the management of patients with advanced disease. Additional efforts, including the current study and ongoing and planned clinical trials, will help to better define the role for targeted therapy in UC. Additionally, the use of biomarkers to select treatment is infrequently used with the exception of programmed death-ligand 1 (PD-L1) protein expression for first line immune checkpoint inhibitors (ICI) and *FGFR2/3* alterations for erdafitinib^10,11^. There is an unmet need to identify biomarkers with clinical utility.

Most biomarker research has been performed as retrospective efforts in clinical trials. In an attempt to leverage “real world” experience to build a clinical database and biospecimen repository to develop and validate biomarkers, the Bladder Cancer Advocacy Network (BCAN) aligned efforts with academic institutions to design the Urothelial Cancer – Genomic Analysis to Improve Patient Outcomes and Research (UC-GENOME) study. The overarching goal was to provide every patient with metastatic UC the opportunity to become an exceptional responder with molecular analyses for the identification of predictive as well as prognostic biomarkers. The project launched in 2016 with two co-equal aims: (1) to provide a comprehensive next generation sequencing report including DNA mutations, copy number alterations (CNA), fusions, and tumor mutational burden (TMB), with potential therapeutic options including clinical trials, at no cost to patients; and (2) to create a biorepository and clinical database to perform foundational analyses including DNA and RNA sequencing for future collaborative research efforts.

Here, we present the foundational molecular characterization of the UC-GENOME dataset including DNA and RNA sequencing with analyses incorporating the clinical data elements to provide an overarching view of the tumor and the tumor microenvironment in this real-world population of patients with metastatic UC.

## Results

### Patient characteristics/data description

**Fig. 1:**
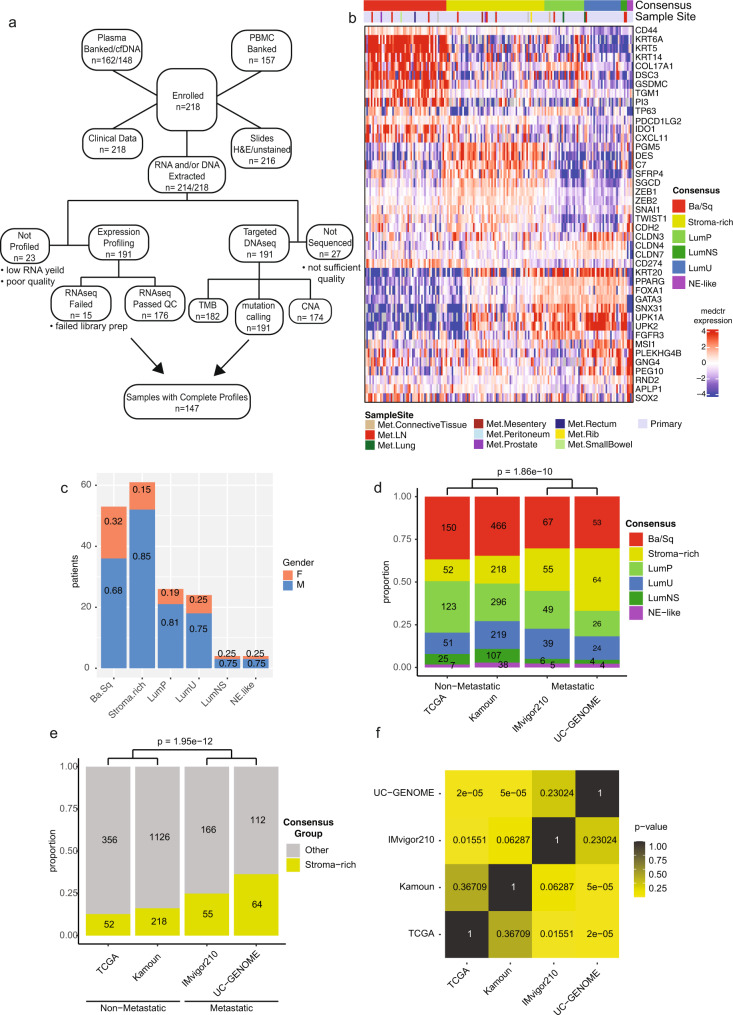
UC-GENOME study design and molecular subtyping shows enrichment of Stroma-rich primary tumors in metastatic datasets. **a** Consort diagram showing enrollment and sample banking along with downstream sample attrition and available data. **b** Subtype calls were made on the UC Genome data for the consensus subtypes. Log2 median centered mRNA expression for selected bladder specific markers were sorted by the Consensus subtype call. **c** Gender distribution by subtype. Y-axis represents the number of patients. The frequency of gender within each subtype is shown within their respective portion of the barplot. **d** Subtype distribution across non-metastatic (TCGA/Kamoun) and metastatic (IMvigor210/UC-GENOME) cohorts. **e** Comparison of Stroma-rich subtypes between all the cohorts. For **d** and **e** the number of samples within each group is shown within their respective portion of the barplot, with the Mantel-Haenszel *χ*^2^ *p*-value is indicated above. **f** Heatmap visualization of the *χ*^2^ *p*-values comparing the proportion of Stroma-rich tumors within each cohort. Source data are provided as a Source Data file.

218 patients with metastatic UC were accrued (Table 1), of which, primary tumors were collected for the majority of patients (87%) with the remaining samples collected from metastatic sites (13%). Most patients were current/former smokers (67.9%) and had a bladder primary tumor at initial diagnosis with high grade and/or invasive disease (83.5%). The most common histologic variants were squamous (15.9%), micropapillary (11%), and adenocarcinoma (5.3%). All other histologic variants occurred at <5% (Supplementary Fig. 1a). Systemic therapy for metastatic UC was administered in 85.8% of patients (chemotherapy (49.1%), ICI (72.9%), targeted therapy (6.9%), and antibody-drug conjugates (11.9%)). With a median follow up from metastatic disease diagnosis of 21.6 months, 127 of 218 (58.3%) patients died (89.8% related to UC). Next generation sequencing (NGS) suggested potential treatment options for 69.3% of patients, however only 5.0% of patients received a targeted therapy based on the results (2.7% received treatment on a clinical trial based on the NGS). DNA and RNA sequencing was successful for 191 and 176 patients, respectively, including 147 samples with complete molecular profiling plus clinical data (Fig. 1a).

**Table 1:** Demographic, clinical characteristics and NGS-based treatment (*n* = 218)

Age at diagnosis	
Mean (range) – years	65.5 (28–85)
Sex – no. (%)	
Male	163 (74.8)
Female	55 (25.2)
Race – no. (%)	
White	172 (78.9)
Black	20 (9.2)
Asian	6 (2.8)
American Indian/Alaska Native	0
Other	0
Unknown	20 (9.2)
ECOG PS – no. (%)	
0	87 (39.9)
1	96 (44.0)
2	28 (12.8)
3	7 (3.2)
Smoking status – no. (%)	
Current	18 (8.3)
Former	130 (59.6)
Never	69 (31.7)
Unknown	1 (0.5)
Tumor origin at initial diagnosis - no. (%)	
Bladder	182 (83.5)
Ureter	12 (5.5)
Renal pelvis	11 (5.0)
Urethra	1 (0.5)
>1 site	12 (5.5)
Primary surgery – no. (%)	
Radical cystectomy	104 (47.7)
TURBT only	52 (23.9)
Radical nephroureterectomy	25 (11.5)
Other	10 (4.6)
No primary surgery	27 (12.4)
Time from initial diagnosis^a^ to metastatic disease	
Median (range) – years	0.8 (0.0–9.8)
Systemic therapy – no. (%)	
Perioperative chemotherapy	100 (45.9)
Neoadjuvant	73 (33.5)
Adjuvant	31 (14.2)
Any systemic therapy for metastatic disease	187 (85.8)
Chemotherapy	107 (49.1)
Immunotherapy	159 (72.9)
Targeted therapy	15 (6.9)
Antibody-drug conjugate therapy	26 (11.9)
Unknown	4 (1.8)
Survival^b^ - no. (%)	
Alive	91 (41.7)
Dead	127 (58.3)
Cause of death – no. (%)	
Disease-related	114 (89.8)
Treatment-related	4 (3.2)
Other	6 (4.7)
Unknown	3 (2.4)
NGS-based treatment decisions – no. (%)	
NGS provided treatment options	
Yes	151 (69.3)
No	63 (28.9)
No response	4 (1.8)
Any targeted therapy received per NGS	11 (5.0)
Targeted therapy on clinical trial per NGS	7 (3.2)

^a^Initial diagnosis with high grade or invasive disease.

^b^Survival status at last follow up. Median time from metastatic disease diagnosis to last follow up: 1.8 year (0.1–17.7).

### Transcriptional profiling reveals enrichment of Stroma-rich primary tumors in metastatic datasets

The Kamoun et al.^3^ consensus molecular subtypes (Fig. 1b) and the underlying expanded subtyping classifications (Supplementary Fig. 1b) were observed in our UC-GENOME cohort with gene expression patterns prototypical of each subtype. Ba/Sq subtype showed increased expression of basal and squamous markers. The luminal subtypes (Luminal papillary [LumP], Luminal unstable [LumU], and Luminal non-specified [LumNS]) shared expression of luminal markers. The stroma-rich subtype had elevated expression of extracellular matrix and smooth muscle genes as well as epithelial to mesenchymal transition associated genes. Increased expression of immune related genes was observed in Ba/Sq and Stroma-rich tumors. The four NE-like tumors highly expressed neuroendocrine-related genes. As previously described^2,12^, females were numerically, but not significantly, enriched in Ba/Sq (17/53, 32%) versus other subtypes (23/117, 19.6%; *p* = 0.08, Fig. 1c). Of the histologic variants, samples with squamous differentiation were significantly enriched in the Ba/Sq subtype (*p* < 0.0001; Supplementary Fig. 1c). We next evaluated if either ECOG or age was associated with subtype. There was no significant association between ECOG and age, nor either variable with subtype (Supplementary Fig. 1d–f).

We compared the subtype specific gene expression patterns of UC-GENOME to a cohort of patients with non-metastatic UC, TCGA BLCA^12^, and a metastatic cohort, IMvigor210^13^. We observed high correlation of gene expression (UC-GENOME versus IMvigor210, mean correlation 0.79 and UC-GENOME versus TCGA, mean correlation 0.72; Supplementary Fig. 1g). While the subtype expression patterns were similar, the subtype distribution differed between non-metastatic and metastatic cohorts (Fig. 1d). A significant increase in proportion of stroma-rich tumors was observed in the metastatic datasets, IMvigor210 and UC-GENOME, compared to non-metastatic cohorts, TCGA and Kamoun (Fig. 1e, f and Supplementary Data 1).

To determine if this was due to bias from the specimen site (i.e. tissue from a primary tumor -vs- tissue from a metastasis), we performed principle component analysis (PCA; Supplementary Fig. 2a, b) and hierarchical clustering (HC; Supplementary Fig. 2c), pseudo-coloring by consensus subtype either primary/met tissue (PCA) or the collection site (HC). Whereas the PC1 and PC2 separate samples into their indicated subtypes, all the metastatic tissue samples fall within the variance range of the primary tissue. Furthermore, with hierarchical clustering, the metastatic tissue samples do not group by either metastatic tissue nor metastatic site, indicating that tissue origin does not confound the gene expression analysis. Additionally, when performing the analysis with primary samples only (Supplementary Fig. 2d, e), the significant enrichment of Stroma-rich tumors within the metastatic UC-GENOME and IMvigor210 cohorts persists (Supplementary Fig. 2f).

### Targeted DNA sequencing demonstrates a high prevalence of TP53 E285K mutations in UC

**Fig. 2:**
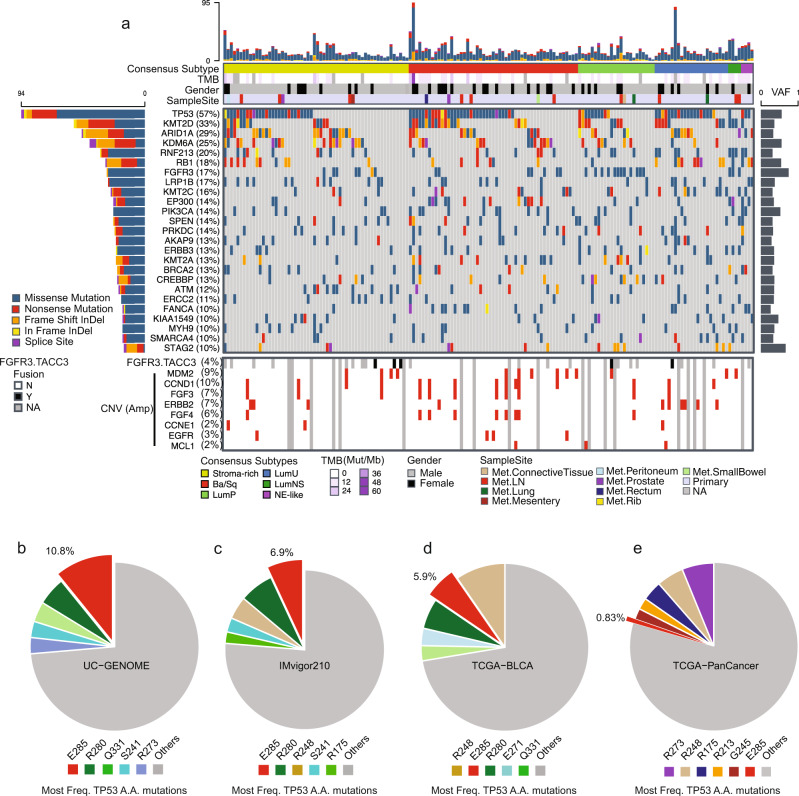
Targeted DNA sequencing analysis demonstrates a high prevalence of *TP53 E285K* mutations in UC. **a** Oncoplot of UC-GENOME bladder tumor samples sequence variant measurements, with annotation tracks for consensus subtype, tumor mutation burden (TMB), and gender. The top 25 genes based on frequency across samples of non-silent variants are shown. Percentages indicate number of tumor samples of total with an identified variant in each respective gene. Gene fusions and the most frequent focal amplifications of those assayed (Methods section) are shown in lower panel. VAF = average variant allele frequency, InDel  = Insertion or deletion, Mb = Megabase. **b** Number of *TP53* E285 mutations by tumor type across TCGA. The percent of the indicated variant within the total number of *TP53* mutations for **b** UC-GENOME, **c** Imvigor210, **d** TCGA BLCA, and **e** TCGA PanCancer. The variants shown are the top 5 most frequently mutated sites plus E285 (if not otherwise present). Source data are provided as a Source Data file.

DNA sequencing of 591 genes was performed on 191 patients including 166 patients with RNA data (Fig. 1a and Supplementary Data 1). At least one non-silent variant was observed in 552 of the 591 genes sequenced. Similar to TCGA^12^, we observed a high prevalence of non-silent variants in *TP53* and *RB1* and the chromatin modifying genes *KMT2D*, *ARID1A, KDM6A, KMT2C, EP300, KMT2A, CREBBP*, and *SMARCA4*, and DNA damage repair genes *BRCA2, PRKDC, ATM, ERCC2*, and *FANCA* (Fig. 2a). We observed frequent hotspot variants in genes associated with aberrant kinase signaling including *FGFR3* (S249C) and *PIK3CA* (E545K) (Supplementary Fig. 3a, b). As previously described, *FGFR3* mutations were enriched in LumP tumors (OR 7.2, p-val 6.1e-5)^5,12^ (Fig. 2a). *FGFR3-TACC3* gene fusions were found in 4% of evaluable samples (Fig. 2a) including one tumor which also had a *FGFR3* S373C mutation. Analysis of CNVs identified amplifications of *CCND1* (10%), *MDM2* (9%), *ERBB2* (7%), *CCNE1* (2%), and *EGFR* (3%) at similar frequency as in TCGA^12^.

The most frequently mutated gene, *TP53*, had an increased frequency of mutation in Ba/Sq and LumU tumors with a corresponding increase in an expression-based signature of p53 pathway dysfunction (Supplementary Fig. 3c). The most frequent *TP53* variant was E285K (11%) (Fig. 2b and Supplementary Fig. 3d). The E285 variant was observed at a higher frequency in the metastatic cohorts (UC-GENOME, 10.8%, and IMvigor210, 6.9%) than in the primary TCGA cohort (5.9%) (Fig. 2b–d). Of all *TP53* mutations across the PanCancer cohort from TCGA, which consists of >10,500 tumors (>4,250 *TP53* mutations), the *TP53* E285K variant was the 30th most frequently mutated amino acid (0.83%), with bladder cancers accounting for 39% (15/38) of the total E285 variants (Fig. 2e).

### Mutational signature profiling reaffirms the importance of APOBEC in UC

**Fig. 3:**
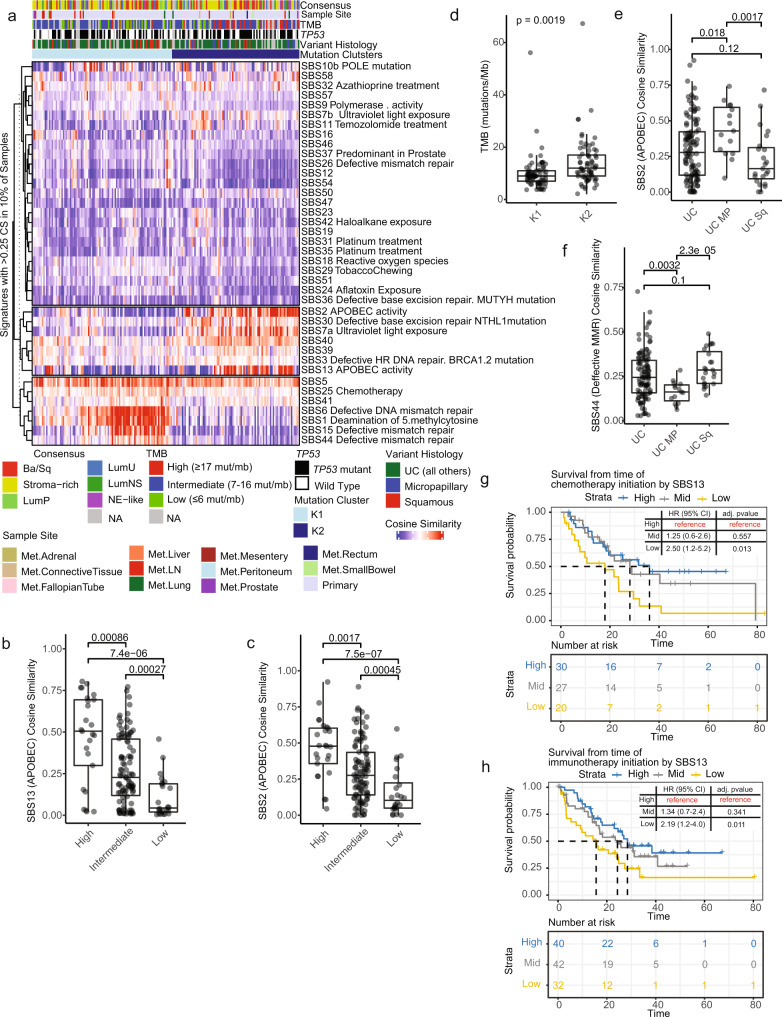
Mutational signature clustering and analysis reaffirms the importance of APOBEC in UC. **a** Heatmap of mutational signatures with cosine similarity (CS) > 0.25 in at least 10% of samples. The samples (*n* = 191 samples) were sorted by the consensus cluster plus (CCP) clusters and clustered by signature. **b**, **c** Boxplots were generated for the CS of SBS13 (**b**) and CS of SBS2 (**c**) for patients divided into TMB tertiles (*n* = 191 samples). **d** Boxplot of TMB by K1 and K2 CCP cluster (*n* = 191 samples). **e** SBS2 and **f** SBS44 were plotted by variant histology for those samples with annotated histology (*n* = 147 samples). All boxplots are shown with boxes representing the IQR and midline at the median. Error bars represent Q1/Q3  ±  1.5*IQR. Two-sided Wilcoxon test *p*-values are shown above the given comparison. Kaplan-Meier curves were used to visualize survival from time of treatment initiation for **g** chemotherapy and **h** immunotherapy. Samples were split into high, medium, and low APOBEC activity based on the tertiles of the rank order CS for SBS13. Cox proportional-hazard modeling was performed with the high group as reference, Hazard Ratio (95% CI) and adjusted *p*-value for each comparison are inset with risk tables below. Source data are provided as a Source Data file.

Mutational signatures and shared patterns of single nucleotide variants (SNVs) can identify potential sources of mutagenic pressure, either intrinsic (mutations in DNA repair genes) or extrinsic (carcinogen exposure)^14^. By performing consensus clustering of the per-sample cosine similarity (CS) to the published COSMICv3 mutation signatures, we identified two distinct sample clusters (Fig. 3a, Supplementary Fig. 4a). Cluster 1 (K1) had high CS with defective DNA mismatch repair (MMR) signatures, while Cluster 2 (K2) had a high CS with APOBEC activity signatures (SBS2 and SBS13) (Fig. 3a). Independently, high TMB tumors were associated with increased APOBEC activity signatures SBS2 and SBS13 (Fig. 3b, c), this was represented in our clustering as K2, which in addition to having increased APOBEC, had increased TMB (*p* = 0.002). Cluster 2 was numerically, but not statistically enriched for TP53 mutations (K1 = 46/87 [53% mutant], K2 = 46/75 [61% mutant], *p* = 0.34). TMB (*p* = 0.002) and both APOBEC mutational signatures (SBS2 [*p* = 0.02] and SBS13 [*p* = 0.002]) were also significantly higher in the *TP53* mutant samples (Supplementary Fig. 4b–d).

The CS for SBS2, but not SBS13, was significantly elevated in micropapillary histology compared to tumors with squamous differentiation (*p* = 0.002) or all other UC tumors (*p* = 0.018) (Fig. 3e and Supplementary Fig. 4e). Micropapillary tumors were also significantly associated with increase POLE mutation signature versus squamous (*p* = 0.026) and other UC tumors (0.02; Supplementary Fig. 4f). In contrast, tumors with squamous differentiation had a significantly increased cosine similarity to mismatch repair defect signatures, such as SBS44, as compared to micropapillary (*p* = 2.3e-5) but not other UC tumors (Fig. 3f).

To evaluate the relationship between APOBEC activity and survival following either chemotherapy or ICI, samples were subdivided into 3 groups according to the CS to SBS2 and SBS13. Having a low CS to SBS13, but not SBS2, was associated with decreased survival as calculated from the initiation of treatment for both chemotherapy (*p* = 0.013) and ICIs (*p* = 0.011) (Fig. 3g, h and Supplementary Fig. 5a, b). Overall survival from the time of diagnosis, however, was not significantly different among the CS group for SBS2/SBS13 (Supplementary Fig. 5c, d), suggesting that CS levels of SBS2 and SBS13 are not merely prognostic.

### Ba/Sq tumors are T cell inflamed but luminal tumors are enriched in memory B cells and plasma cells

**Fig. 4:**
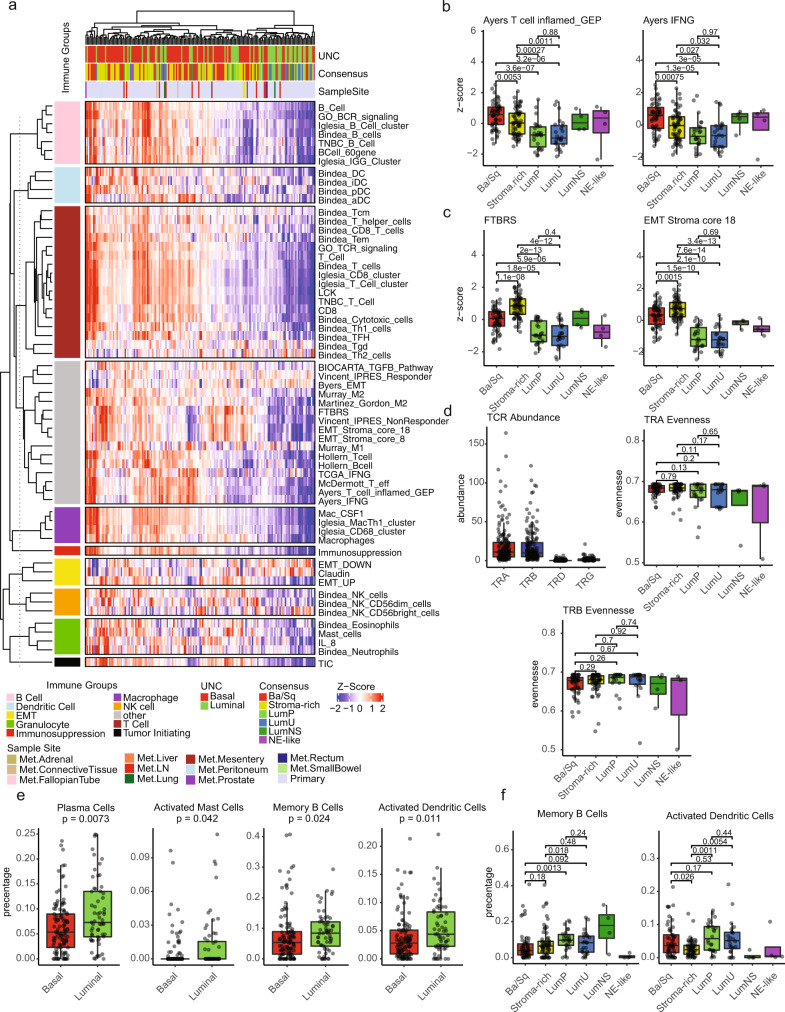
Ba/Sq tumors are T cell inflamed but luminal tumors are enriched in memory B cells and plasma cells. **a** Tumor samples (*n* = 176 samples) were clustered by samples and Immune Gene Signature (IGS) z-scores. IGS were clustered within their respective immune group (right of dashed line). Boxplots for signatures related to ICI response and **b** T-cells or **c** stroma/EMT were plotted by *z*-score and consensus subtype (*n* = 176 samples). **d** TCR family abundance and evenness were estimated from RNA using MiXCR and plotted by Consensus subtype (*n* = 153 samples). **e** Selected CIBESORTx cell types that were significantly associated with either **e** UNC subtypes or **f** Consensus subtypes were plotted by boxplot (*n* = 176). Wilcoxon *p*-values are shown above plot. All boxplots are shown with boxes representing the IQR and midline at the median. Error bars represent Q1/Q3  ±  1.5 × IQR. Source data are provided as a Source Data file.

Prior studies have described differential levels of T cell inflammation associated with the UC molecular subtypes^3,9,15^. The tumor microenvironment (TME) of UC-GENOME was characterized through immune gene expression signatures (IGS) curated from the literature^13,16–19^. As a whole, IGS scores were elevated in Ba/Sq and Stroma-rich subtypes as compared to the luminal subtypes (LumP, LumU, LumNS; Fig. 4a). The Ba/Sq subtype had significantly higher expression of genes within the T cell inflamed and IFNG signatures (Fig. 4b) while LumP and LumU had the lowest median expression. The Stroma-rich subtype had significantly higher scores, as compared to the other subtypes, for IGS related to the presence of stromal infiltration, namely, Fibroblast-TGF-β Response Signature [FTBRS] and EMT_Stroma_core_18 (Fig. 4c). These two signatures have previously been associated with resistance to ICI^18,19^.

We next used MiXCR to infer TCR clonality of tumor infiltrating T cells (Fig. 4d). We identified high abundance of TCR-alpha (TRA) and TCR-beta (TRB), while TCR-gamma (TRG) and TCR-delta (TRD) chains did not have adequate abundance and were excluded from subsequent analyses. There were no differences in evenness (inverse of clonality) across consensus subtypes for TRA or TRB chains (Fig. 4d).

Immune cell proportions were estimated by CIBERSORTx. Using the UNC two subtype classification, most immune cell types were enriched in the UNC basal subtype as previously described^15^, with the exception that luminal tumors were high for plasma cells, activated mast cells, memory B cells, and activated dendritic cells (Fig. 4e). Using the consensus subtypes, memory B cells and activated dendritic cells were also significantly differentially enriched in LumP (Fig. 4f). No correlations were observed between IGS and TMB^20^ or TMB and subtype (Supplementary Fig. 6a–d).

**Fig. 5:**
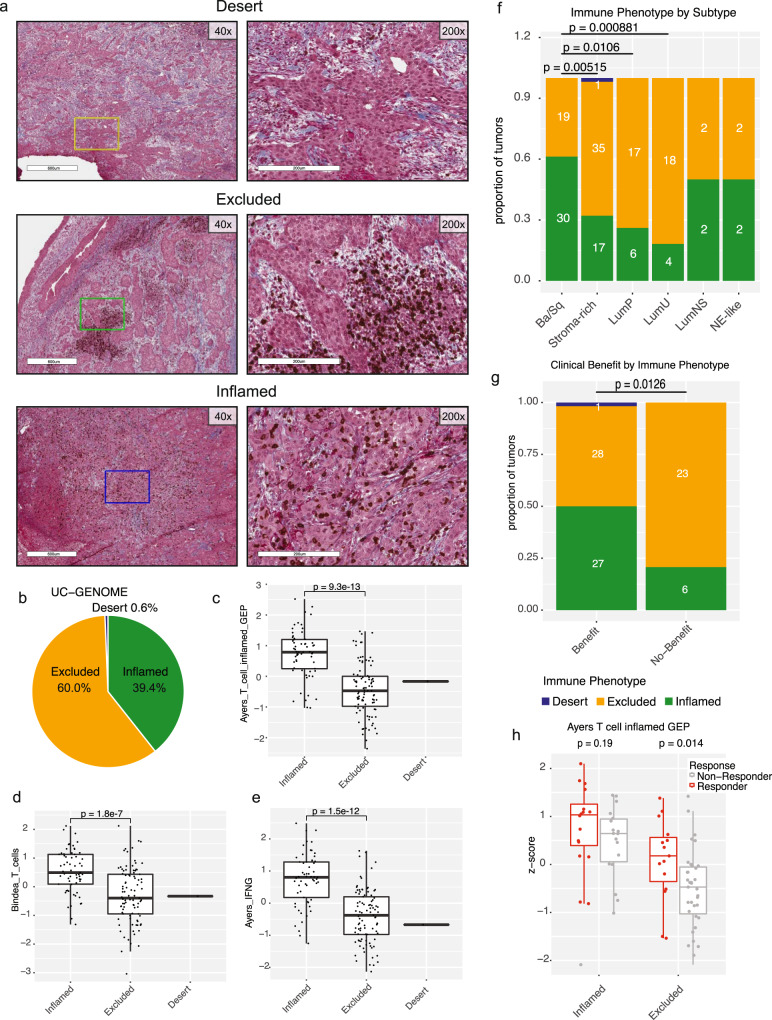
Immune phenotyping demonstrates enrichment of Inflamed tumors in Ba/Sq subtype. **a** FFPE slides were stained with anti-CD8 and Masson’s Trichrome and the pattern of CD8 + cells were evaluated. Samples were either called as “Desert”, “Excluded”, or “Inflamed”. Representative images of the three phenotypes are shown at a total magnification of ×40 (left) and ×200 (right). The area shown at ×200 is boxed within the ×40 image (Desert = yellow, Excluded = green, Inflamed = blue). The area within the box on left is the tumor portion at right. **b** the distribution of immune phenotypes within UC-GENOME (*n* = 155 samples). **c** Boxplots of Ayer’s T cells inflamed, **d** Bindea T cells, and **e** Ayer’s INFG signatures by immune phenotype. Wilcoxon p-values are shown above the comparison groups (*n* = 155 samples). All boxplots are shown with boxes representing the IQR and midline at the median. Error bars represent Q1/Q3  ±  1.5 × IQR. **f** Stacked barplots of the immune phenotype distribution across the consensus subtypes and **g** clinical benefit to ICI are shown with the number of samples within each bar at center. Lines at top represent the comparison groups, with *p*-values calculated using fisher’s exact test. **h** Group boxplot of the Ayer’s T cells inflamed *z*-score by immune phenotype and response status (*n* = 84 samples). Two-sided Wilcoxon *p*-values for responders versus non-responders for each phenotype is shown at top. Source data are provided as a Source Data file.

We next performed histologic immune phenotyping by dual staining FFPE slides with an antibody against CD8 and Masson’s Trichrome. Tumors were either called “Desert” if lacking CD8 + cells, “Excluded” if CD8 + cells were restricted to the stroma, or “Inflamed” if CD8 + cells were seen in both the stroma and infiltrating the tumor (see Methods section, Fig. 5a). 155 tumors were suitable to make phenotype calls, of which 93 (60%) were determined to be Excluded, 61 (39.4%) were labeled as Inflamed, and 1 (0.6%) called Desert (Fig. 5b). Inflamed tumors had significantly elevated IGS scores for multiple T cell signatures, including: Ayers T cell inflamed (*p* = 9.3e-13), Bindea T cell (*p* = 1.8e-7) and Ayers IFNG (*p* = 1.5e-12) (Fig. 5c–e). Since we had seen these aforementioned signatures are elevated in the Ba/Sq subtype (Fig. 4a, b), we asked whether there was an immune phenotype bias in any of the consensus subtypes. Indeed, the Ba/Sq subtype was significantly enriched in the Inflamed phenotype as compared to the Stroma-rich (*p* = 0.005), LumP (*p* = 0.01), and LumNS (*p* = 0.0009; Fig. 5f). This Ba/Sq enrichment was also present within the IMvigor210 dataset (Supplementary Fig. 6e). We next assessed the relationship between the histologic immune phenotype and clinical benefit (CR + PR + stable disease [SD]). Patients who experience benefit from ICI were split, 28/27 between Excluded and Inflamed, however patients who did not have any clinical benefit were significantly enriched for the Excluded phenotype (*p* = 0.0126) (Fig. 5g). Among Excluded tumors, those with response (complete response [CR] + partial response [PR]) to ICI also had, albeit not significantly, increased levels of Ayers T cell inflamed signature score (*p* = 0.19; Fig. 5h). Our analysis confirms the important role of stroma in promoting resistance to ICI^13,18^ in addition to the association between IHC subtype and molecular subtype, the enrichment of IHC Inflamed and IHC Excluded tumors in the Ba/Sq, and Stroma-rich tumors respectively.

### Clinical response to chemotherapy and ICI: an exploratory analysis

**Fig. 6:**
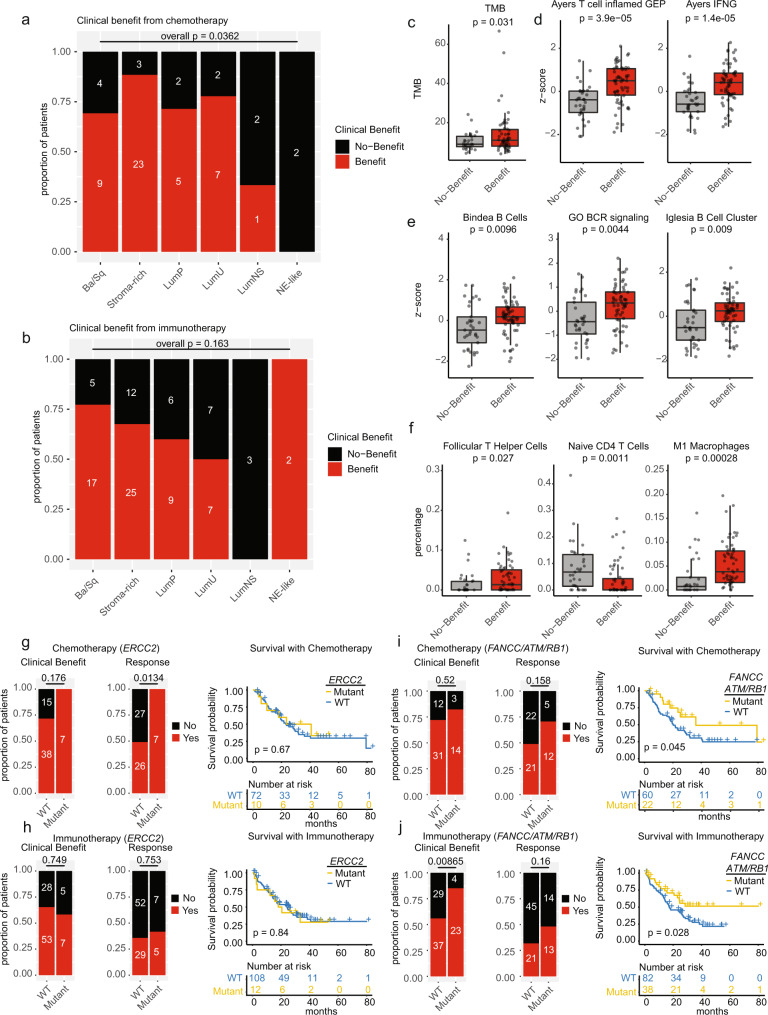
Molecular associations with clinical benefit and response. **a** Clinical benefit to chemotherapy and **b** immunotherapy are shown as stacked bar plots with No-Benefit (progressive disease) (black) and clinical benefit (complete response, partial response, and stable disease) (red) displayed by subtype. Bars are the proportion of patients within each subtype, n represents the absolute number in the group. Clinical benefit to ICI plotted by **c** TMB (*n* = 157 samples) and IGS scores (*n* = 176 samples) for **d** Ayers T cell inflamed GEP and Ayers IFNG, **e** Bindea B-cells, GO BCR signaling, Iglesia B cell cluster, and **f** CIBERSORTx proportions of follicular helper T cells, naïve CD4 T cells, and M1 macrophages. All boxplots are shown with boxes representing the IQR and midline at the median. Error bars represent Q1/Q3  ±  1.5 × IQR. Two-sided Wilcoxon *p*-values are shown above the comparison. Clinical benefit and response, along with KM plots for overall survival to chemotherapy or immunotherapy are shown for patients who had tumors with either **g**, **h** *ERCC2* mutations or **i**, **j** *FANCC*, *ATM*, and *RB1* mutations. Source data are provided as a Source Data file.

Previous studies have interrogated the predictive and prognostic value of UC subtypes^4^. UC-GENOME patients, stratified by consensus subtype were assessed for correlations to response and clinical benefit to chemotherapy or ICI. Overall, 55% of patients (33/60) responded to chemotherapy, however, within subtype analysis identified Stroma-rich (17/26) and LumU (7/9) as having >50% response (Supplementary Fig. 6f). In contrast, clinical benefit with chemotherapy was seen in 75% of tumors, with only LumNS (1/3) and NE-like (0/2) having a minority of patients derive clinical benefit (Fig. 6a). The response rate to ICI within UC-GENOME was 36% (34/93), with no subtype having greater than a 50% response (Supplementary Fig. 6g). In contrast to response, at least 50% of patients within all subtypes derived clinical benefit to ICI (with the exception of LumNS), with an overall clinical benefit rate of 65% (60/93; Fig. 6b).

ICI clinical benefit was associated with increased TMB (Fig. 6c), T cell inflamed, and IFNG IGS scores (Fig. 6d). Increased B cell abundance and signaling (Fig. 6e) along with an increase in the percentage of T follicular helper (Tfh) and M1 macrophage and decreased percentage of naïve CD4 T cells, were also associated with increased clinical benefit to ICI (Fig. 6e, f). However, immune infiltration signatures, FTBRS and EMT Stroma, were not associated with ICI response either within the cohort as a whole or solely within IHC Excluded tumors as documented previously^13^ (Supplementary Fig. 6h, i).

Somatic mutations in *ERRC2* or a mutation in *FANCC, ATM, or RB1* have been previously shown to predict response to neoadjuvant cisplatin-based chemotherapy^21,22^. In UC-GENOME, *ERCC2* mutations were associated with a significantly higher response to chemotherapy, validating this biomarker in a metastatic setting; however, it was not associated with ICI response nor improved overall survival with chemotherapy or ICI (Fig. 6g, h). There was no association with clinical benefit or response between chemotherapy and *RB1*, *ATM*, or *FANCC* mutations. However, patients with these alterations did have an improvement in survival from the time of initiation of treatment as compared to patients without these alterations. Patients with *RB1*, *ATM*, or *FANCC* mutations who received ICI showed an increased clinical benefit, but not response, along with an improved overall survival (Fig. 6i, j).

### An integrated model of clinical and genomic features predicts response to ICI

**Fig. 7:**
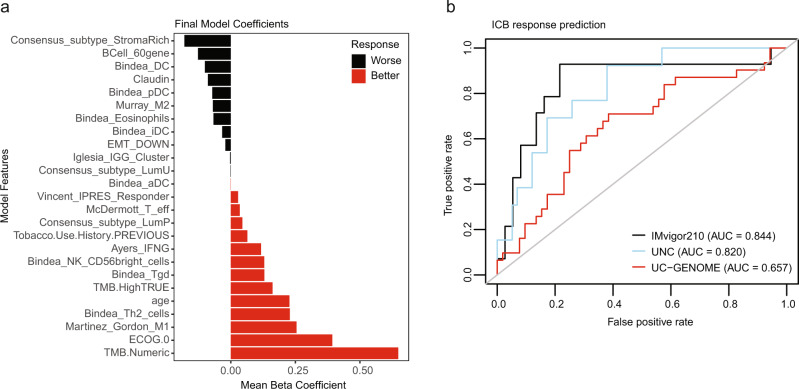
An integrated elastic net model of clinical and genomic features predicts response to ICI. **a** The predictor coefficients are shown for the final model trained on the entire discovery set. **b** The receiver operating characteristic curves are shown for the model performance on the validation portion of the IMvigor210 dataset (*n* = 51), and the entire UNC-108 (*n* = 88), and UC-GENOME (*n* = 166) independent datasets. Source data are provided as a Source Data file.

The potential to better predict benefit from ICI has important implications for optimizing patient care. While several potential biomarkers have been examined in isolation, few studies have integrated clinical and genomic features to develop predictive models^23–27^. We used clinical and immunogenomic data (Supplementary Data 2) from a discovery set of ICI treated patients with metastatic UC (IMvigor210) and applied elastic net (EN) logistic regression modeling to develop an integrated predictive model of ICI response. From 50-fold cross-validation on the discovery set, features of worse response included: B cell gene signature, stroma-rich consensus subtype, and claudin signature (Supplementary Fig. 7a), while features of better response were: baseline ECOG performance score 0, TMB, and M1 macrophage signature. The final EN model consisted of 25 predictors, similar to the cross-validation results (Fig. 7a).

The performance of the final EN ICI predictor model was tested on the validation portion of the IMvigor210 dataset (*n* = 51), UC-GENOME, and an additional independent dataset (UNC-108)^28^ (Fig. 7b). This model accurately predicted ICI response in the IMvigor210 validation set (AUC = 0.84) and in UNC-108 (AUC = 0.82) datasets. While the model did not perform as well in the UC-GENOME cohort, it did perform significantly better then random chance (AUC = 0.65, *p* = 0.009). Elevated model prediction scores were significantly associated with ICI response in the IMvigor210 validation set (Supplementary Fig. 7b), and in UNC-108 (Supplementary Fig. 7c). Importantly, the final EN ICI predictor was significantly better at predicting ICI response than a model using TMB alone (AUC = 0.84 vs. 0.68, *p* = 0.038) as well as other modeling approaches (Supplementary Fig. 7d, e). The final EN model was not predictive of response to chemotherapy (AUC = 0.536, *p* = 0.345) (Supplementary Fig. 7f) demonstrating its specificity to ICI as well as arguing that it does not merely model for favorable prognosis.

## Discussion

We present the results of the foundational analyses of the BCAN-led UC-GENOME project including the integration of DNA and RNA sequencing data with clinical variables to describe the molecular landscape of UC. The UC-GENOME cohort and associated data allowed us to uncover a number of metastatic related features as we compared our results with non-metastatic datasets (TCGA and Kamoun et al.)^3,9^ such as: a higher proportion of Stroma-rich molecular subtype and enrichment of the *TP53* E285K hotspot mutation in primary tumors from metastatic patients. Additionally, we present and validate an integrated model of clinical and genomic factors associated with ICI outcome in this “real world” population of patients with metastatic UC.

Of the >4,000 *TP53* mutations in Pan-TCGA tumors, the *TP53* E285K mutation accounted for only 0.83% (*n* = 38). However, of the *TP53* E285K mutations, 39% (*n* = 15) were from bladder tumors, even though the bladder samples only account for 5.6% of all *TP53* mutations. Notably, this mutation is an APOBEC-attributable hotspot mutation^29^ and was enriched in metastatic UC cohorts. One possible explanation is that upregulated APOBEC activity promotes tumor progression and *TP53* E285K mutations are merely a manifestation of elevated APOBEC activity but do not promote metastases. Arguing against this, however, is that APOBEC-high tumors have longer overall survival. Moreover, of the top 10 most frequent APOBEC-associated hotspot mutations^29^ only the *TP53* E285K mutation was enriched in our metastatic UC-GENOME cohort relative to the non-metastatic cohort (TCGA-BLCA). These findings are consistent with a putative model whereby elevated APOBEC activity cultivates the APOBEC-associated *TP53* E285K hotspot mutation, which actively promotes tumor progression. This model would account for the enrichment of *TP53* E285K in patients with metastatic UC.

We examined previously reported putative biomarkers (*ERCC2*, *ATM, FANCC, or RB1*) associated with response to neoadjuvant chemotherapy (NAC)^21,22^. In UC-GENOME, *ERCC2* mutations were significantly enriched in chemotherapy responders while mutations in *ATM, FANCC*, and *RB1* were not; neither biomarker was associated with prolonged OS. The lack of association between *ATM, FANCC*, or *RB1* mutation and response may be secondary to the use of carboplatin (rather than cisplatin) in a significant proportion of patients as these biomarkers were developed in cohorts uniformly treated with cisplatin-based NAC. Alternatively, *ATM, FANCC*, and *RB1* mutations may have different biological consequences in different disease states (MIBC -vs- metastatic). While Teo and colleagues have shown that patients with mutations in DNA damage and repair (DDR) genes have enhanced response to platinum-based chemotherapy^30^, we present that *ERCC2* mutations specifically may serve as a biomarker associated with platinum-based chemotherapy response in the metastatic setting.

We and others have documented subtype-specific differences in the tumor microenvironment and in general have concluded that tumors of the Ba/Sq subtype express higher levels of IGS. Other subtypes with relatively high IGS expression include UNC Claudin-low^15^ and Consensus Stroma-rich tumors^3^. We were therefore struck by the increased proportion of CIBERSORTx cell types: plasma cells, memory B cells, and activated dendritic cells in UNC Luminal tumors. These cell types are associated with tertiary lymphoid structures (TLS) and accumulating evidence correlates the presence of TLS with ICI response^31^. In this context, the correlation between the proportion of CIBERSORTx defined CD4 + Tfh cells and B cell IGS with ICI response are also consistent with TLS playing an important role in ICI response in the UC-GENOME cohort as multiple reports in breast cancer have shown that CD4 + Tfh cells lie in close proximity to germinal center B cells and are important regulators of antigen specific B cell responses^19,32^.

Recent observations have underscored the importance of spatial biology of tumors as it relates to ICI^13,33^. Additionally in bladder cancer, the importance of tumor stroma (i.e. cancer associated fibroblasts [CAFs]) as a negative predictor of ICI response has been demonstrated by multiple groups^13,18,28^. Herein, we performed a similar analysis, staining for CD8 and trichome on FFPE sections with assignment of immune phenotype (Inflamed, Excluded, and Desert) by a GU pathologist. We report that the relationship between immune phenotype and molecular subtype finding that Ba/Sq tumors are enriched in the Inflamed immune phenotype while Stroma-rich, LumP, and LumU tumors have a higher proportion of Excluded tumors. Moreover, our work validates the negative impact of stroma on ICI response as there was a significant enrichment of Excluded tumors in UC-GENOME patients without clinical benefit to ICI. Nonetheless, much work on the spatial biology of bladder cancer remains to be performed.

We developed an elastic net model that integrates both clinical and immunogenomic variables to predict ICI response. Our EN ICI predictive model performs better then random chance or TMB alone in all validation datasets examined but had a higher degree of accuracy in IMvigor210 and UNC-108 than UC-GENOME (Fig. 7b). Drawing from prior published work from Jialu et al., one potential factor for the performance disparity between the cohorts could relate to the different methods used for generating the transcriptomic data (used in downstream IGS)^34^. While IMvigor210 and UNC-108 were generated through capture-based RNA sequencing, the UC-GENOME transcriptome data were generated using total RNAseq. Indeed, when we calculated univariate regressions of each immune signature versus ICI response for each data set, we found that the regression of β-coefficients were strongly correlated between IMvigor210 and UNC-108 (*R* = 0.73) but weakly correlated between UC-GENOME and IMvigor210 (*R* = 0.36) and between UC-GENOME and UNC-108 (*R* = 0.36; Supplementary Fig. 7g–i).

Several challenges and limitations of the current study deserve attention. Although targeted DNA sequencing was employed rather than whole exome or whole genome sequencing, the significantly mutated genes detected were consistent with prior sequencing studies. While the study achieved its goal of providing NGS at no cost to the patient with reported treatment options including potential clinical trials for most patients (69%), only 5.0% of patients received a targeted therapy and 2.7% receiving targeted therapy on a clinical trial based on the NGS results. The likely reasons for the low utilization of NGS results to guide treatment include the absence of FDA approved targeted therapies along with the development and FDA approval of immune checkpoint inhibitors during the accrual period (July 2016–May 2019). As the goal of the study was to collect samples within a real-world experience, response was reported based on investigator assessment without the use of formal response criteria, this also limited the number of patients with fully annotated response data. Regular updates to cBioPortal will occur to provide the research community with the most up to date information for future analyses. In terms of the predictive modeling, the EN model can only be applied to the BLCA cohorts since it incorporates UC specific molecular subtypes (i.e. Consensus Stroma-rich). Finally, our analyses are correlative in nature and functional validation will be required to impart causality.

The UC-GENOME project provides a rich biobank (FFPE, plasma, PBMCs) of clinically annotated specimens and their associated molecular data, as described here. The overarching goal of this study was to provide researchers a resource for future collaborative translational research efforts, which can utilize the clinical data, foundational analyses, and bio-banked specimens of this project to advance the development of biomarkers and new treatments for patients with UC. To achieve this goal, The Bladder Cancer Advocacy Network (BCAN) will engage the research community by creating a novel request for proposal mechanism to utilize the UC-GENOME data and specimens toward its mission to advance bladder cancer research and support those impacted by the disease.

## Methods

### IRB approvals

UC-GENOME (ClinicalTrials.gov identifier: NCT02643043) was supported by the Bladder Cancer Advocacy Network (BCAN) and conducted at University of North Carolina at Chapel Hill, Fox Chase Cancer Center, Icahn School of Medicine at Mount Sinai, University of Washington/ Fred Hutchinson Cancer Research Center, Johns Hopkins University, University of Chicago, Memorial Sloan Kettering Cancer Center, and University of Southern California. The study was coordinated by the Hoosier Cancer Research Network (HCRN). The protocol was approved by all of the participating institutional review boards: University of North Carolina Institutional Review Board, Fox Chase Cancer Center Institutional Review Board, Icahn School of Medicine at Mount Sinai Institutional Review Board, Fred Hutch Institutional Review Board, Johns Hopkins Medicine Institutional Review Board, University of Chicago Institutional Review Board, Memorial Sloan Kettering Institutional Review Board, and USC Health Sciences Campus Institutional Review Board. All patients provided written informed consent. The study protocol is available as part of the Source Data file.

Eligible patients had histologically confirmed UC with metastatic disease at the time of registration and tumor tissue available/suitable for molecular analyses. Tumor tissue was obtained from the primary tumor site or in a minority of cases a metastatic site (Table 1). Tumor tissue and blood was collected and banked for future research (Fig. 1a).

As detailed in the trial protocol, the studies primary objectives included: (1) Estimate the proportion of subjects with metastatic UC enrolled who receive NGS and have a personalized report generated with potential treatment options. (2) Create a biospecimen and data repository by collecting and storing blood and archival tumor tissue (biospecimens) from subjects and linking molecular and biological information from those biospecimens to clinical data in order to promote future translational research in metastatic UC. Secondary objectives included (1) Estimate the proportion of subjects whose personalized report includes targeted therapy options (approved or investigational drugs). (2) Estimate the proportion of subjects who enroll in a clinical trial of targeted therapy based on NGS results. (3) Estimate the proportion of subjects who receive targeted therapy (outside of a clinical trial) based on NGS results. (4) Describe the demographics, treatment history and outcomes for subjects enrolled in the study. (5) Document the number and type of clinical trials and basic/translational science or other research projects based on the biospecimen and data repository.

### RNA/DNA

A single block per patient was selected and 4–25 slides (5 and 10 um) from that corresponding block were submitted for processing along with an H&E stained slide. The H&E slide was reviewed, tumor circled, and an estimate of percentage tumor was made by a research pathologist. The pathologist determined how many slides for assay input and requested either full scrape of slide or macrodissection for each slide.

### RNA sequencing

Total RNA sequencing libraries were generated from 500 ng of total RNA isolated from formalin-fixed paraffin-embedded (FFPE) tissue, using the Illumina TruSeq RiboZero Gold protocol (https://support.illumina.com/) and sequenced 75 bp paired-end on an Illumina HiSeq 4000. Reads were aligned to the hg38 genome using STAR^35^ and genes were quantified using Salmon^36^.

Molecular subtyping was performed on log2 transformed upper-quartile normalized expression data using the BLCAsubtyping^3^ and consensusMIBC^3^ R package. Gene annotation conversions were made using biomaRt^37,38^. RNA markers were extracted from Robertson et al.^9^. and visualized using ComplexHeatmaps^39^. To calculate the inter-cohort gene expression correlation, the TCGA BLCA dataset was filtered, retaining genes that were highly (median log2 expression ≥ 5) and variably expressed (STDEV ≥ 1; *n* = 4357 genes); this gene list was then used to filter the UC-GENOME, IMvigo210 and Kamoun datasets. The Spearman correlation was calculated using the median expression (within the indicated subtype/cohort) for each gene retained after filtering.

### DNA sequencing

Tumor only targeted DNA sequencing was performed by Caris Life Sciences (Irving, Texas) on genomic DNA isolated from FFPE tumor samples using a minimum of 50 ng of DNA, of which >20% was required to be of tumor origin, as assessed by H&E staining. The DNA was sequenced via the Agilent custom designed SureSelect XT assay (Caris MI TumorSeek 592-Gene NGS Panel, details at www.carislifesciences.com) on the Illumina NextSeq platform. Copy number variation was determined by comparing the depth of sequencing of genomic loci to a diploid control as well as the known performance of these genomic loci. Gene fusion and variant transcript detection were performed on mRNA isolated from FFPE tumor samples using the Archer FusionPlex Solid Tumor Panel and sequenced on Illumina MiSeq.

Variants were analyzed with maftools^40^. For visualization, only the most disruptive variant based on SIFT and PolyPhen classifications was chosen when a sample had multiple variants per gene. TCGA variant data was obtained from cBioPortal^41^.

### Mutational signatures

The R package SomaticSignatures^42^ was used to identify mutational signatures in the UC-GENOME cohort (*n* = 191). Signatures were compared to 49 Single Base Substitution (SBS) signatures from COSMIC version 3^14^ with cosine similarity (CS). To determine the optimal number of somatic signature clusters we chose COSMIC signatures in which 10% of samples have greater than >0.25 cosine similarity (CS) and clustered samples using Consensus Cluster Plus (CCP)^43^ for all samples. The CS signature heatmap was visualized with Complex Heatmaps^39^. For boxplots and survival curves using the mutational signatures, the dataset was reduced to samples with both RNA and DNA sequencing data available (*n* = 176). Samples were binned into APOBEC High/Mid/Low by rank ordering the samples by the CS to the indicated APOBEC signature and splitting by tertiles.

### Characterization of the tumor microenvironment and immune signatures

Z-scores were calculated based on all genes within previously published immune gene signatures on a per sample basis^13,16–19^. Immune cell fractions were calculated by CIBERSORTx using the default parameters^44^.

TCR clonality was analyzed via MiXCR from RNAseq data to generate TCR clonotype expression matrices for each sample. Data was analyzed with custom scripts as well as the tcR package.

CD8 (Cell Marque, catalog #108R-16, clone: SP16, dilution: 1:400)/Masson’s trichrome (Fisher Scientific, catalog #22-110-648) stained slides were reviewed by a fellowship trained genitourinary pathologist to determine immune phenotype. Slides were scanned at ×100 magnification to evaluate for overall tumor cellularity and staining quality. In areas of invasive tumor, slides were then evaluated at ×200 for CD8 + immune cell infiltrates (brown chromogen). As per Mariathasan^13^, “Desert” categorization required less than or equal to 10 CD8 + T-cells identified in the specimen, averaged over 20 fields (i.e., less than 100 CD8 + in 10 fields). “Excluded” categorization required localization of immune infiltrates to the stroma without extension into the tumor nests. These immune infiltrates were present in both the stroma located between nests in an invasive focus (intra-tumoral), and at the interface between invasive tumor and deeper stroma (peri-tumoral). “Inflamed” categorization required infiltration of CD8 + T cells into the tumor nests with direct contact of immune cells and tumor cells. Scattered CD8 + T cells seen within tumor, without overt stroma infiltrates, were identified in some cases and were not considered sufficient for “Inflamed” and were called “Exluded.

### Elastic net

Eight clinical variables (TMB, TMB high [>10 mutations/Mb], ECOG performance status, age, sex, tobacco use, prior platinum, and prior BCG), consensus subtype, and 61 immune gene signature scores were evaluated as potential predictors of response to ICI. Three datasets, IMvigor210^13^, UNC-108^28^, and UC-GENOME were used for discovery and validation. Within each dataset, continuous variables were standardized (mean = 0.5, SD = 0.5) and samples with any NA values were omitted. The IMvigor210 dataset was divided into discovery (*n* = 104) and validation (*n* = 51) sets with balanced immunotherapy response. In the discovery set, elastic net regression with 50-fold cross-validation was used to build an optimal logistic model for response using the R package caret (tuneLength = 15). The β-coefficient mean and 95% confidence interval for each predictor were calculated separately using 50-fold cross-validation with the tuning parameters from the optimal logistic model. Performance of the final model was evaluated in the validation portion of IMvigor210, UNC-108, and UC-GENOME. Performance of the final model in IMvigor210 was compared with the performance of random forest and gradient tree boosting using the R package caret.

### Statistical analysis

Categorical variables were compared using Fisher’s exact or chi-square test. Continuous variable comparisons were made using *t*-test or Wilcoxon rank sum (in cases of non-normal distribution). All boxplots are shown with boxes representing the IQR and midline at the median. Error bars represent Q1/Q3  ±  1.5 × IQR. Correlations were performed using Pearson correlation unless otherwise stated. Multiple comparison correction was performed using Bonferroni correction. Survival analyses were performed using Kaplan–Meier with log-rank tests. Statistical analyses were performed using R unless otherwise noted.

### Reporting summary

Further information on research design is available in the Nature Research Reporting Summary linked to this article.

## Supplementary information

Supplementary Information

Description to Additional Supplementary Information

Supplementary Data 1

Supplementary Data 2

Reporting Summary

Source_Data_Files

## Data Availability

The UC-GENOME mutation annotation file (MAF), gene expression matrix and clinical data generated in this study have been deposited in the cBioPortal database under “Urothelial Carcinoma, Nature Communications (BCAN/HCRN 2022)” [https://www.cbioportal.org/study/summary?id=blca_bcan_hcrn_2022]. All RNA and DNA FASTQ files are available on dbGaP, phs003066.v1.p1 [https://www.ncbi.nlm.nih.gov/projects/gap/cgi-bin/study.cgi?study_id=phs003066.v1.p1] under restricted access for disease specific research, with the exception of samples obtained from Johns Hopkins University (JHU). The JHU samples are only available to investigators from non-profit entities with an IRB conducting disease specific research, under dbGaP study ID phs003094,v1.p1 [https://www.ncbi.nlm.nih.gov/projects/gap/cgi-bin/study.cgi?study_id=phs003094.v1.p1]. The data used to generate the figures in this study as well as the code for the elastic net classifier are provided in the Supplementary Information/Source Data file and also via FigShare.com [https://figshare.com/articles/journal_contribution/UC-GENOME/19491287]. Upper quartile normalized RSEM gene expression data and DNA sequencing data for TCGA were downloaded from the GDC legacy archive [https://portal.gdc.cancer.gov/legacy-archive/]. IMvigor210 data were downloaded from the European Genome-Phenome Archive, EGAS00001004343 [https://ega-archive.org/studies/EGAS00001004343]. UNC-108 RNA sequencing data were obtained from GEO, GSE176307 [https://www.ncbi.nlm.nih.gov/geo/query/acc.cgi?acc=GSE176307] and DNA sequencing data were obtained through request of the authors. Source data are provided as a Source Data file.

## Code availability

The elastic net modeling code has been provided in the Source Data file and via .
