# Supplementary material for: Collaborative study from the Bladder Cancer Advocacy Network for the genomic analysis of metastatic urothelial cancer
Source: Nat Commun. 2022 Nov 4;13:6658. doi: 10.1038/s41467-022-33980-9 (PMC9636269; doi:10.1038/s41467-022-33980-9)
Supplement: Supplementary file 1 — Supplementary Information [file 41467_2022_33980_MOESM1_ESM.pdf]

Supplementary Figure 1

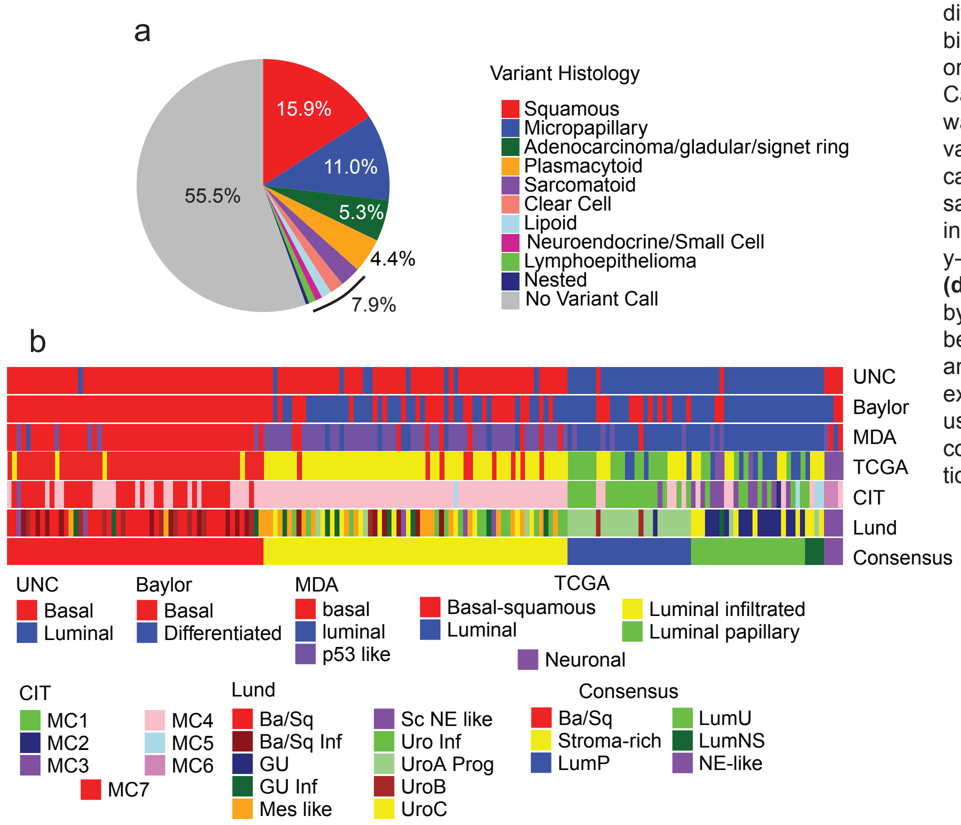

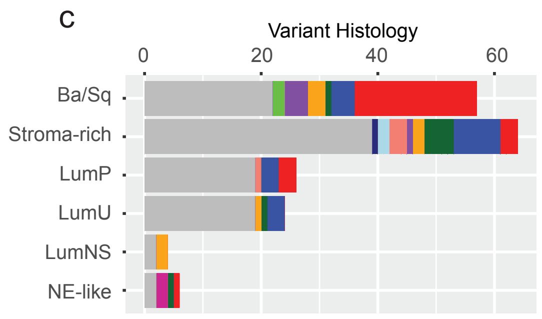

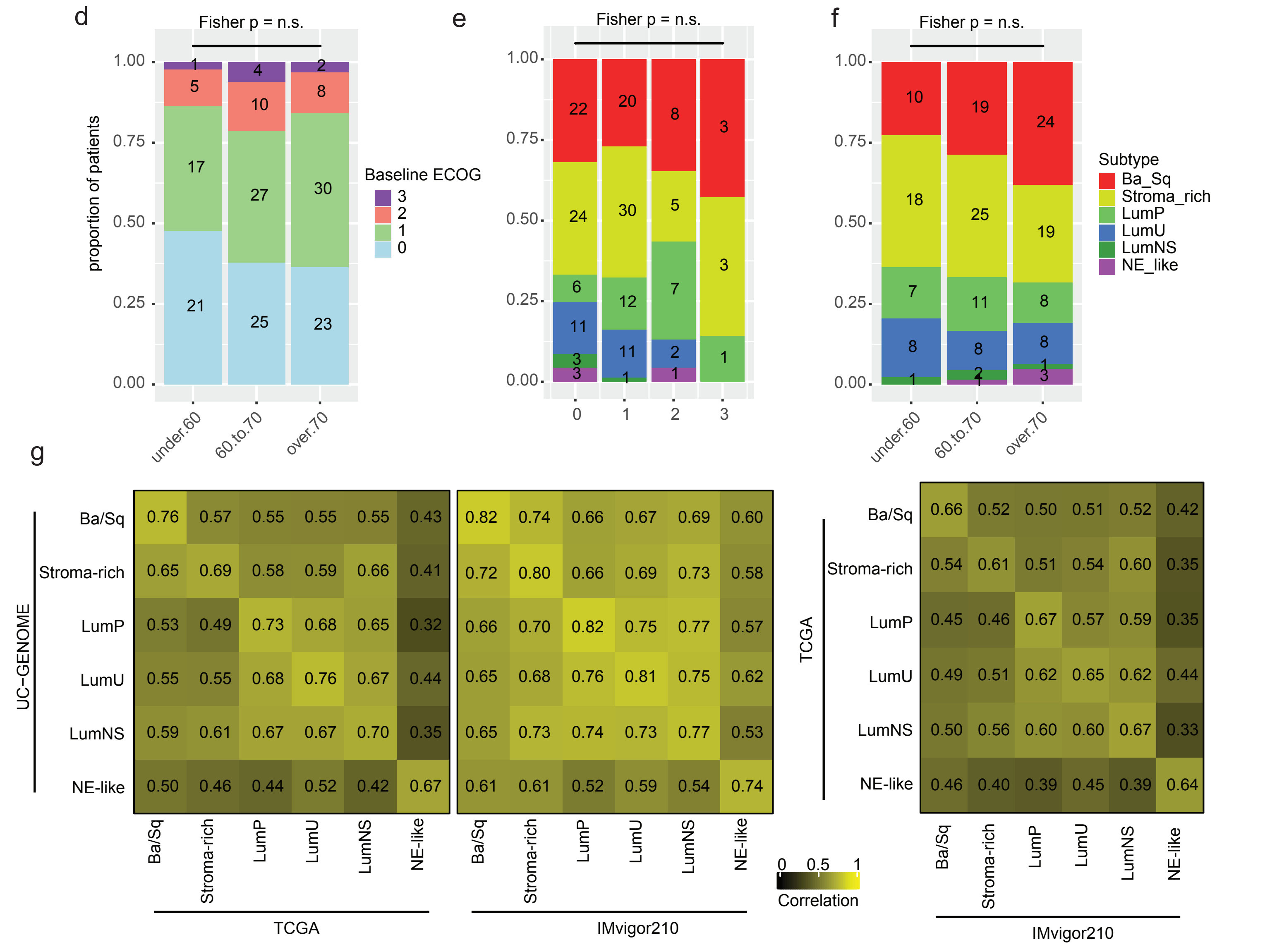

Supplementary Figure 1: Histologic variants and divergent differentiation, as notated by the tissue source site, were binned into 1 of 10 histology types. If no variant was present or provided, the sample was categorized as “No Variant Call”. **(a)** The relationship of the variants at the cohort level was visualized as a pie chart. The frequency of the indicated variant with the cohort is inset within the slice. **(b)** Subtype calls for all samples with RNA sequencing data (n=176). The samples were then sorted based on the consensus subtyping calls. **(c)** The variants group plotted by subtype with the y-axis representing the number of cases per variant/subtype. **(d)** Stacked barplots comparing ECOG by age, **(e)** subtype by ECOG, and **(f)** subtype by age. **(g)** The correlation between the consensus subtypes for UC-GENOME, TCGA, and IMvigor210 were calculated based on the most highly expressed and variable genes. Heatmaps were generated using the correlation coefficient, with black representing low correlation and yellow representing a high level of correlation. Source data are provided as a Source Data file.

Supplementary Figure 2

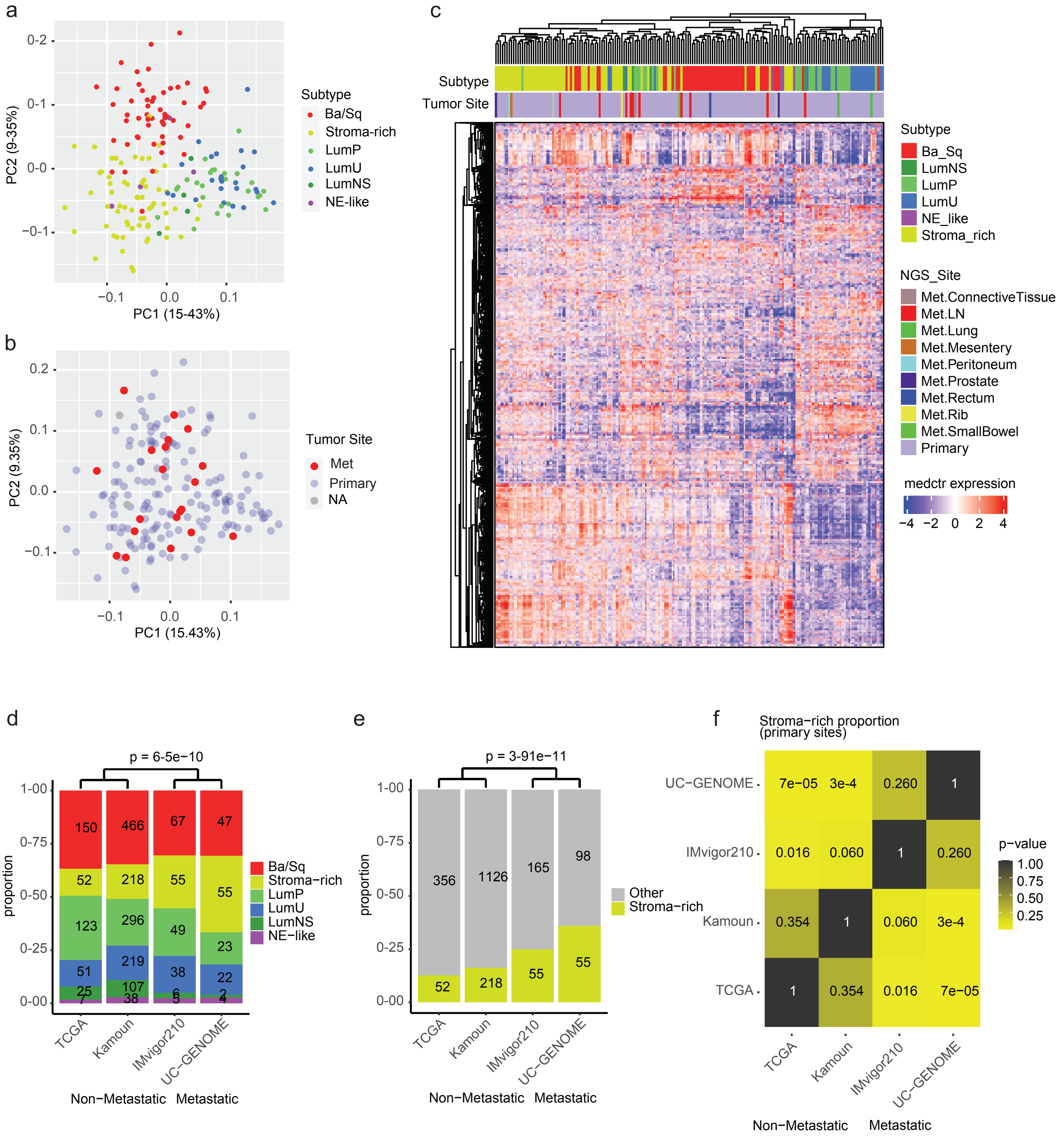

Supplementary Figure 2: (a) Principle component analysis (PCA) was performed using the most high expressed and variable genes pseudo-coloring by subtype and **(b)** either the specimen was take from the primary tumor of metastatic UC or a tumor from a metastatic site. **(c)** The genes used in the prior PCA analysis were then hierarchically clustered and visualized as a heatmap, annotating the consensus subtype and specific site of collection. **(d)** The dataset was then filtered to only include specimens collected from the primary tumor site and distribution of the all subtypes between TCGA, Kamoun, IMvigor210 and UC-GENOME were visualized by stacked barplot, as well as the **(e)** distribution of Stroma-rich vs all other subtypes. The number of samples within each group is shown within their respective portion of the barplot, with the Mantel-Haenszel chi-squared p-value is indicated above. **(f)** The chi-squared p-values comparing the proportion of Stroma-rich subtype between the 4 cohorts was then visualized via a heatmap. Source data are provided as a Source Data file.

Supplementary Figure 3

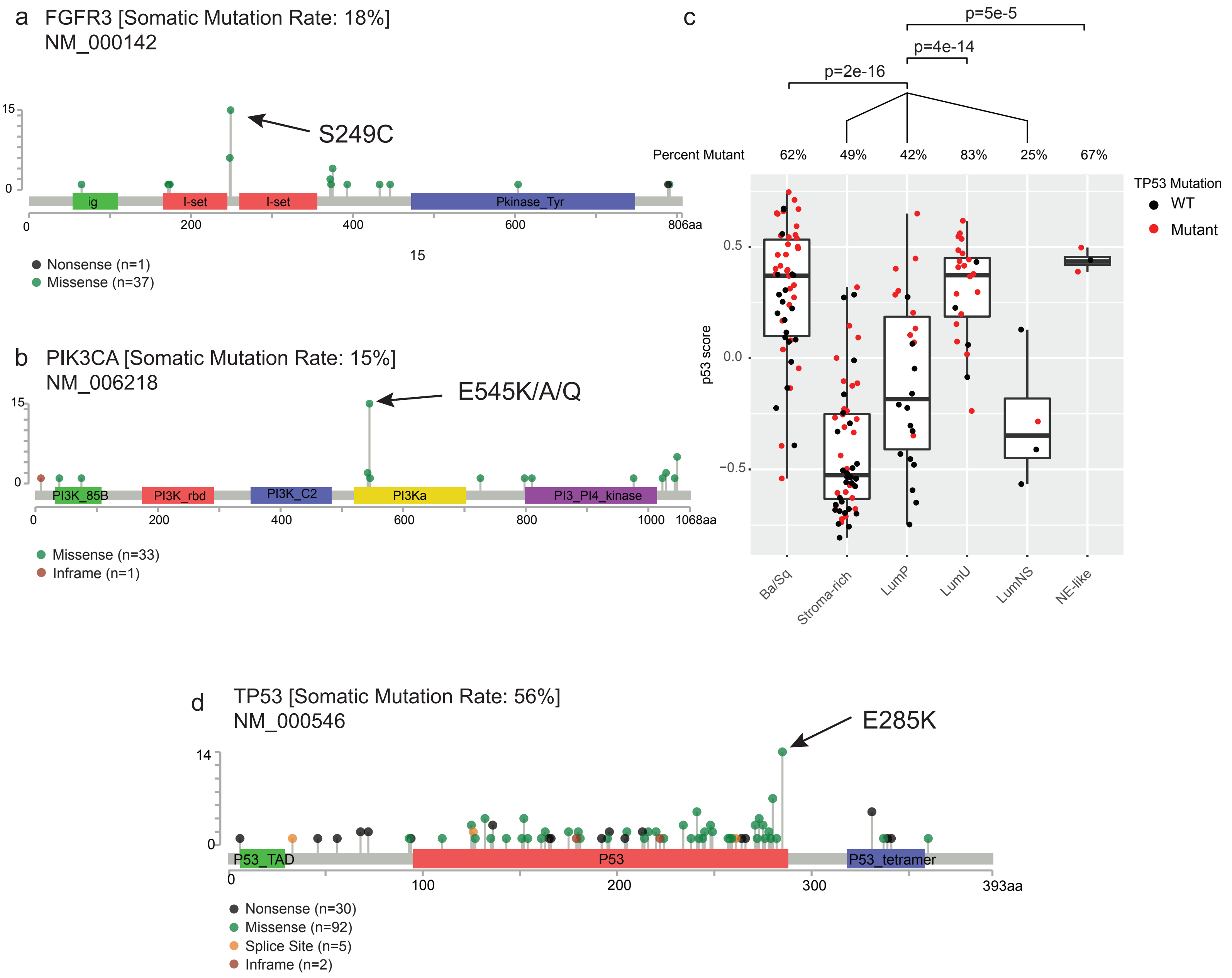

Supplementary Figure 3: Lollipop plots for the frequently mutated kinase signaling genes **(a)** FGFR3 and **(b)** PIK3CA. Colored circles represent mutation class, with the length of the stick proportional to the number of mutations at the given amino acid. **(c)** p53 pathway alteration scores were calculated based on Troester et. al.. The score were plotted by subtype with black points denoting samples WT for TP53 and red points corresponding to TP53 mutant samples (n=176 samples) All boxplots are shown with boxes representing the IQR and midline at the median. Error bars represent Q1/Q3 ± 1.5*IQR. Two-sided t-test p-values are shown for the indicated comparisons. **(d)** Lollipop plot for TP53. Source data are provided as a Source Data file.

Supplementary Figure 4

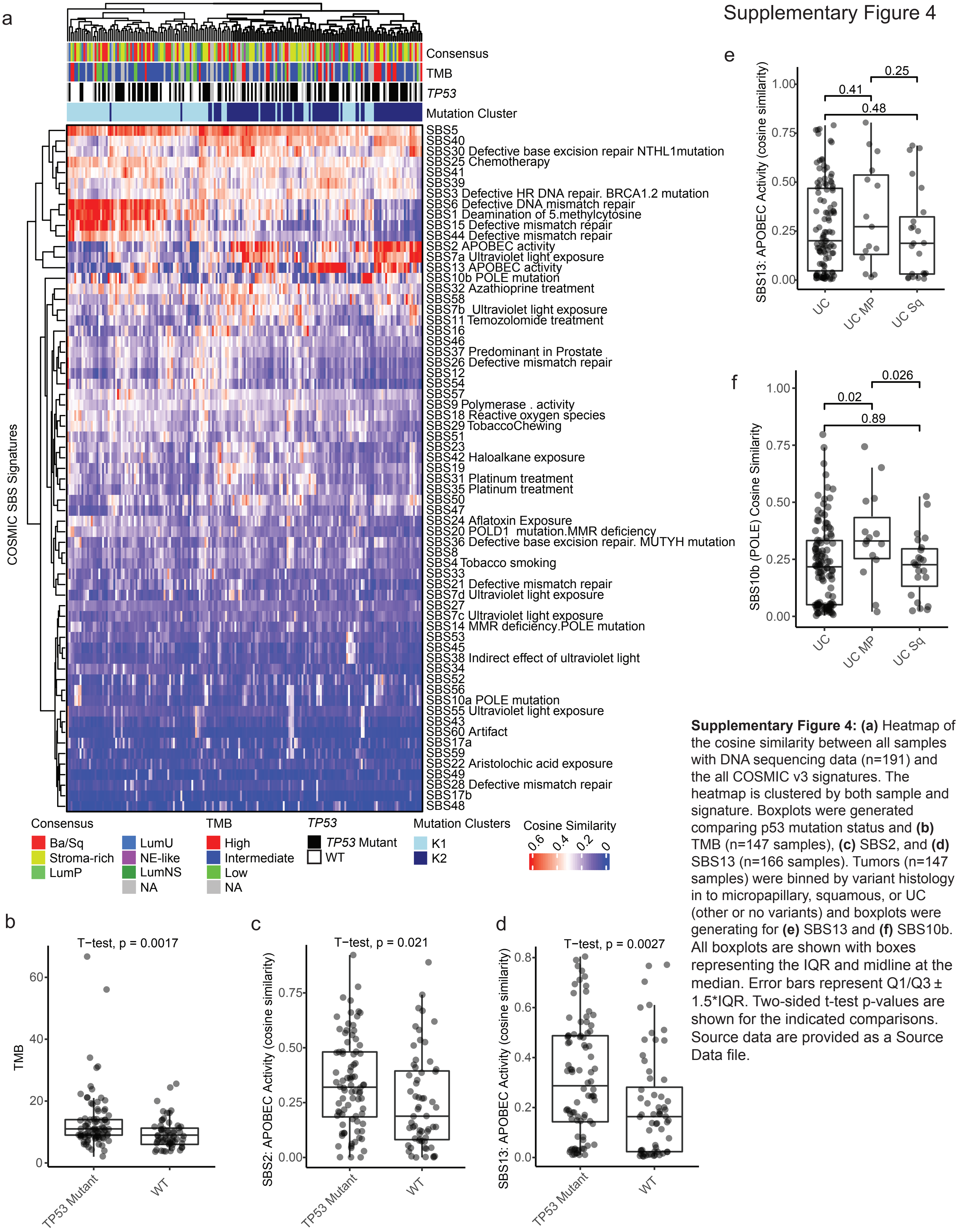

Supplementary Figure 4: (a) Heatmap of the cosine similarity between all samples with DNA sequencing data (n=191) and the all COSMIC v3 signatures. The heatmap is clustered by both sample and signature. Boxplots were generated comparing p53 mutation status and **(b)** TMB (n=147 samples), **(c)** SBS2, and **(d)** SBS13 (n=166 samples). Tumors (n=147 samples) were binned by variant histology in to micropapillary, squamous, or UC (other or no variants) and boxplots were generating for **(e)** SBS13 and **(f)** SBS10b. All boxplots are shown with boxes representing the IQR and midline at the median. Error bars represent Q1/Q3 ± 1.5*IQR. Two-sided t-test p-values are shown for the indicated comparisons. Source data are provided as a Source Data file.

Supplementary Figure 5

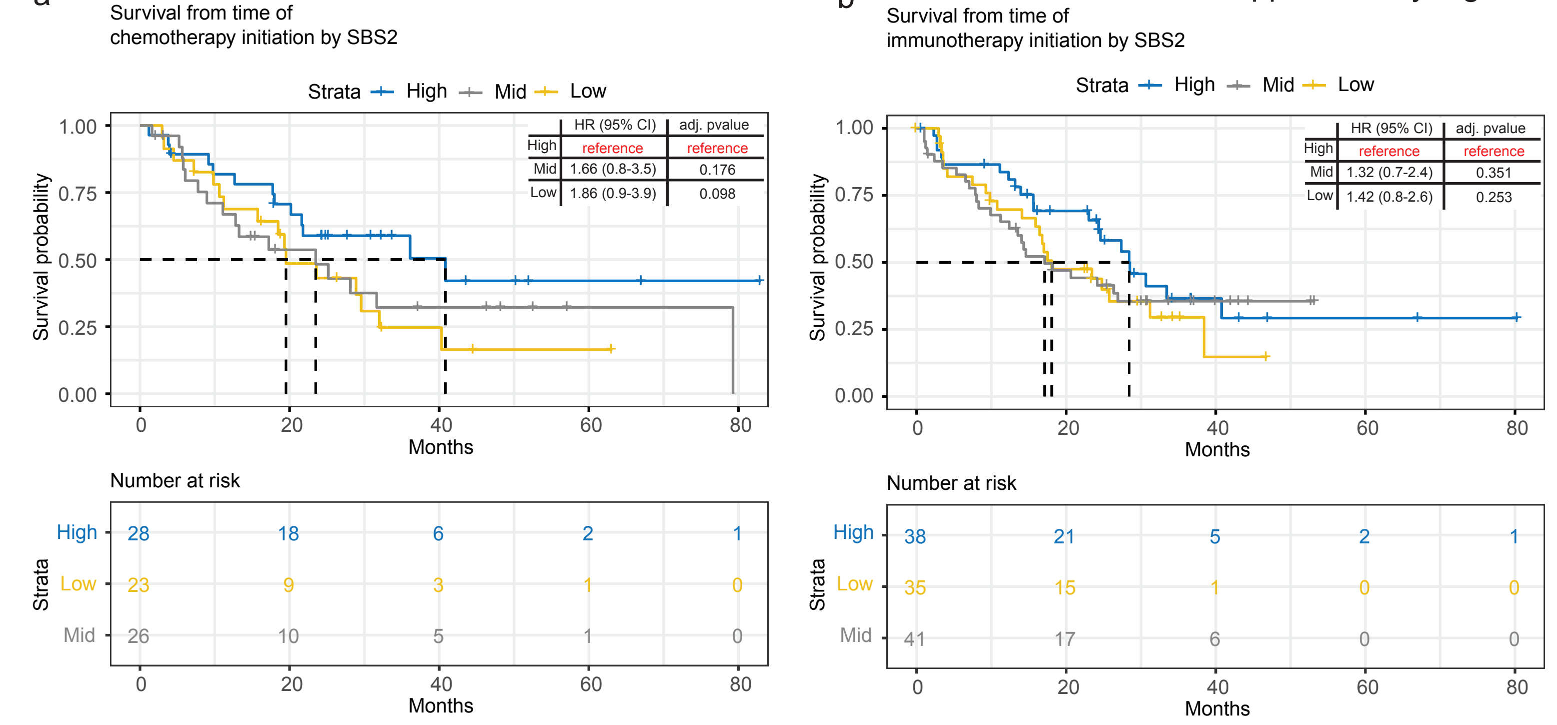

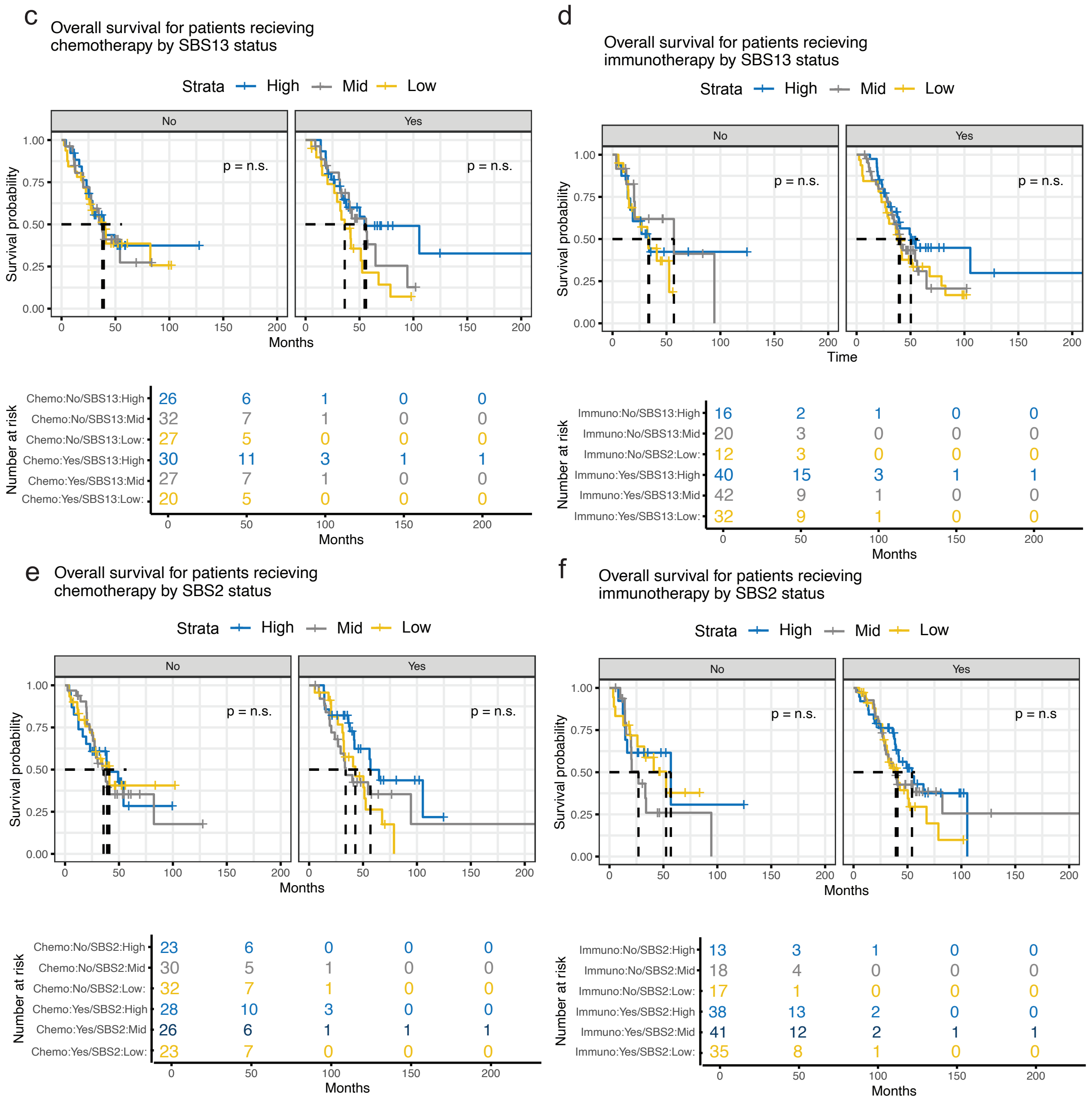

Supplementary Figure 5: Samples were split into high, med and low APOBEC activity based on the tertiles of the rank order cosine similarity for the signature indicated. Kaplan Meier curves were used to visualize survival from time of treatment initiation for **(a)** SBS2 chemotherapy and **(b)** immunotherapy. Cox proportional modeling was performed with the high group as reference, Hazard Ratio (95% CI) and adjusted p-value for each comparison are inset with risk tables below. Overall survival based on the APOBEC activity groups was plotted by Kaplan Meier curve for **(c)** SBS13 chemotherapy and **(d)** immunotherapy, and **(e)** SBS2 chemotherapy and **(f)** immunotherapy. No comparison for overall survival was significant. Source data are provided as a Source Data file.

Supplementary Figure 6

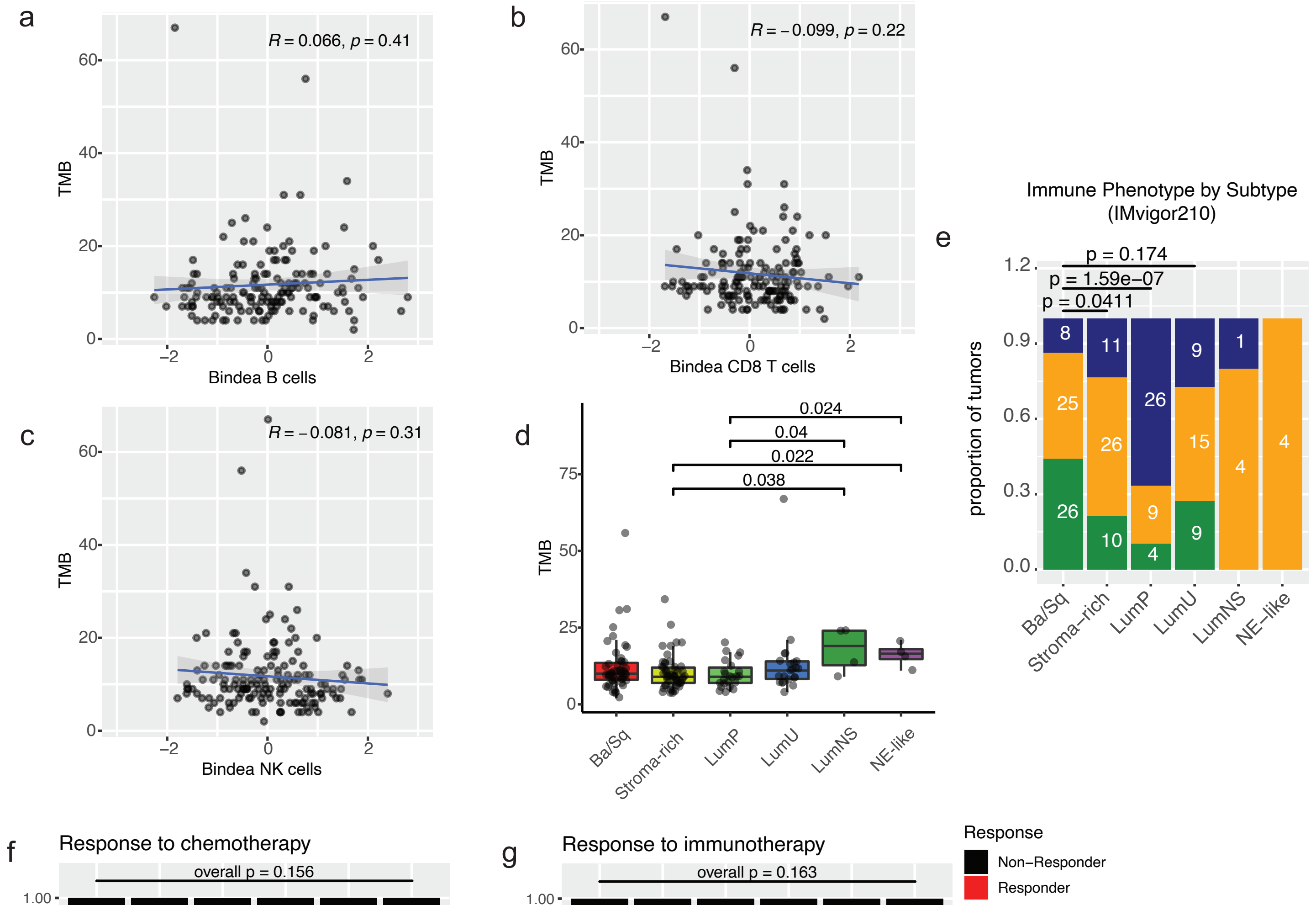

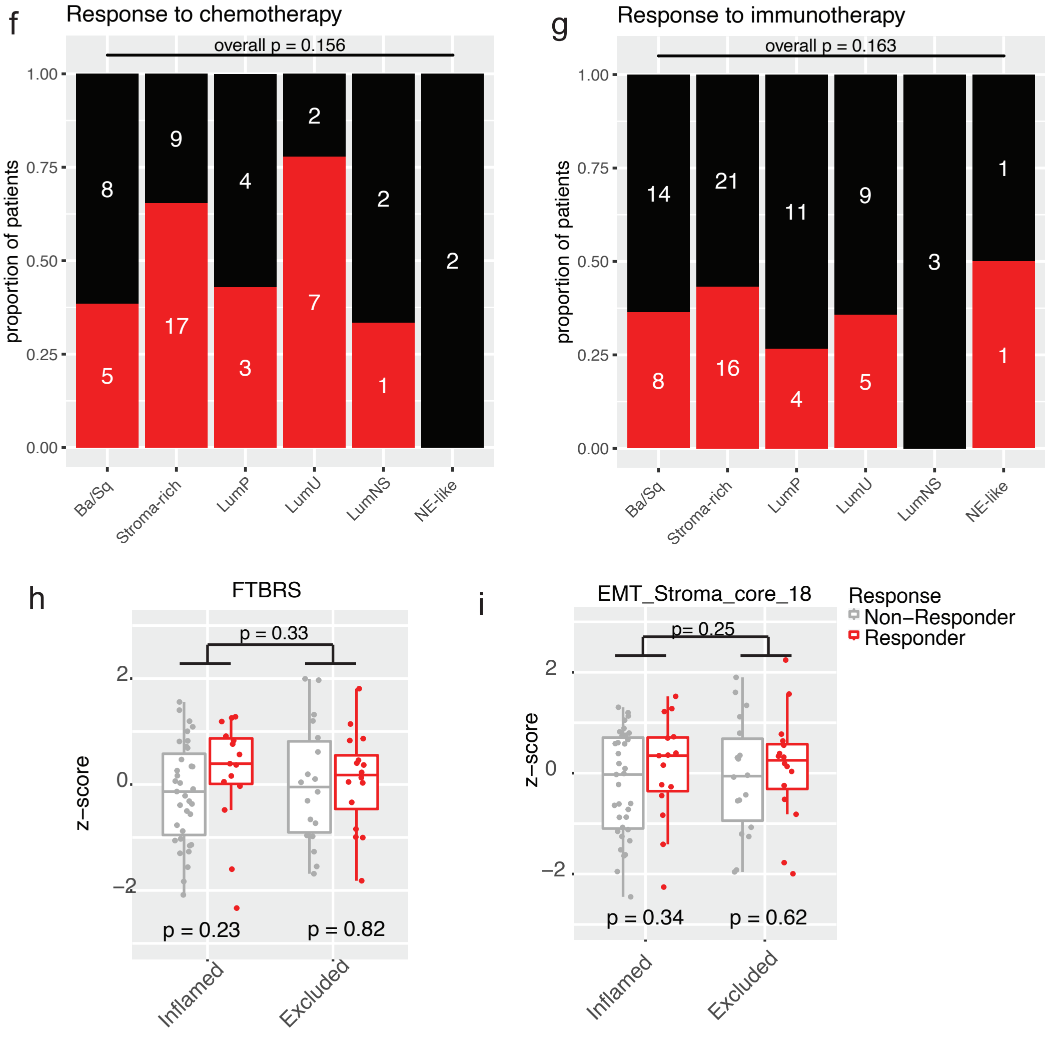

Supplementary Figure 6: TMB (n=157 samples) was compared against the z-score for the IGS from Bindea et. al. for **(a)** B cells, **(b)** CD8 T cells, and **(c)** NK cells. The line of regression line is shown in blue along with the 95% confidence interval. Pearson coefficient and the R value are inset. **(d)** Subtype specific TMB (n=157 samples) was plotted and p-values for significant comparisons (t-test) are shown. **(e)** Immune phenotypes for IMvigor210 were taken from Mariathasan et. al. and were plotted by consensus subtype. Distributions were compared by fisher exact test for the indicated pairs. **(f)** Response to chemotherapy and **(g)** immunotherapy are shown as stacked bar plots with Non-responder (stable and progress disease) (black) and responder (complete or partial response) (red) displayed by subtype. Bars are the proportion of samples within each subtype, n represents the absolute number in the group.**(h)** FTBRS and **(i)** EMT Stroma 18 scores were plotted by responder vs non-responder for the indicated immune phenotype (n=84 samples). Two-sided t-tests were preformed on responder vs non-responders for the each immune phenotype. All boxplots are shown with boxes representing the IQR and midline at the median. Error bars represent Q1/Q3 ± 1.5*IQR. p-values were calculated by two-sided t-tests for the indicated comparisons. Source data are provided as a Source Data file.

Supplementary Figure 7

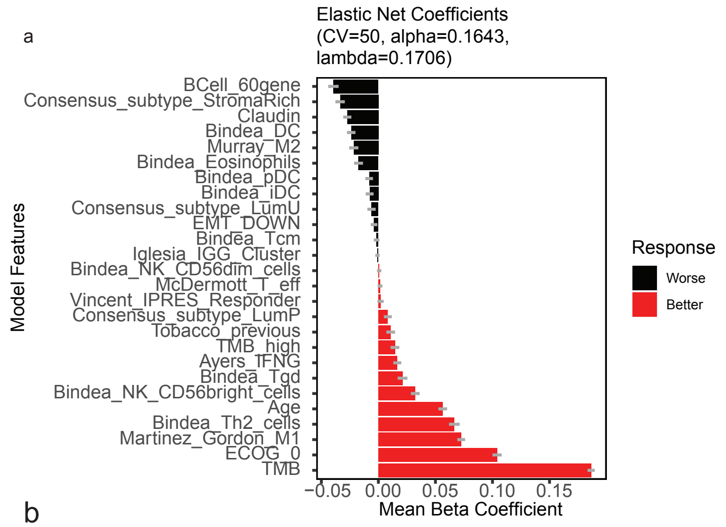

(g)
Supplemental Figure 7: (a) From 50-fold elastic net cross-validation on the IMvigor210 discovery set, means and 95% confidence intervals are shown for the predictors with 95% confidence intervals that exclude 0. Performance was evaluated in the IMvigor210 validation set **(b)** and in **(c)** UNC-108. Density distributions are shown for response and no response by model prediction score (scaled from 0 to 100) with loess regression curves and 0.95 confidence intervals calculated from bootstrapped data (50x, step = 1). Logistic regression of model prediction score by response (response = 1, no response = 0) is shown, and the logistic regression curve and 0.95 confidence interval are plotted. ROC curves are shown for the performance of two other model types—gradient tree boosting and random forest—on the IMvigor210 discovery **(d)** and validation **(e)** sets. **(f)** ROC curves are shown for the performance the EN model on UC-GENOME for ICI repsonse and chemotherapy response. **(g)** The β-coefficients from univariate logistic regressions of immune signatures versus response are compared between the three data sets, **(g)** UC-GENOME/UNC-108**, (h)** UC-GENOME/IMvigor210 and **(i)** IMvigor210/UNC=108, with linear regression lines and 0.95 confidence bands plotted. Spearman R and p-values are shown. Final model parameters: xgbTree; nrounds = 5, eta = 0.05, max_depth = 1, gamma = 0.1, colsample_bytree = 0.8, min_child_weight = 1, subsample = 1) and random forest (rf; seed = 3456, ntree = 2, nodesize = 20. Source data are provided as a Source Data file.

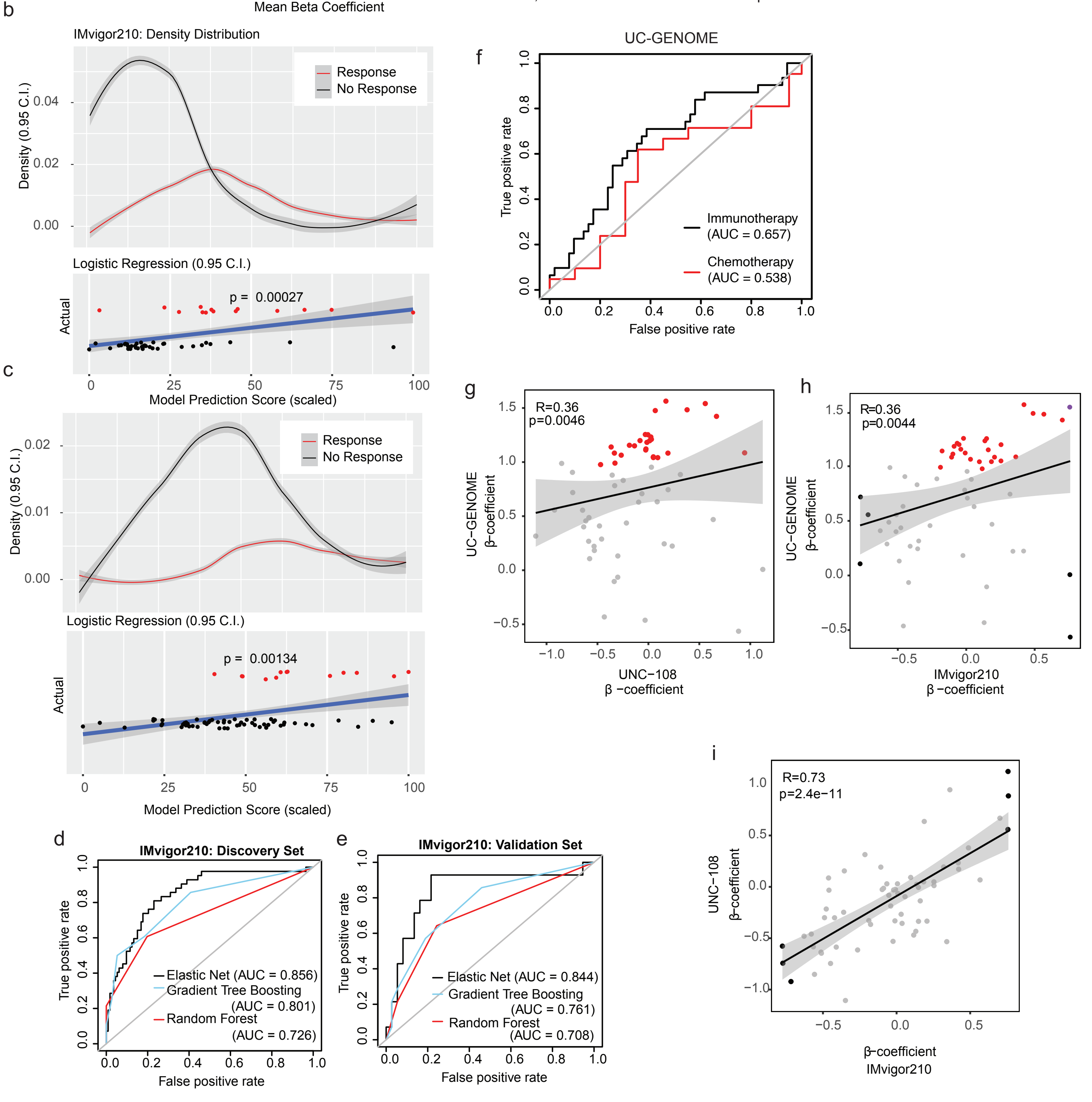

Supplemental Data 1
Caris 592 Gene panel

Supplementary Table 1			
ABL1 (9q34.12)	BCL2L11 (2q13)	CDC73 (1q31.2)	DAXX (6p21.32)
ABL2 (1q25.2)	BCL2L2 (14q11.2)	CDH1 (16q22.1)	DDB2 (11p11.2)
ACKR3 (2q37.3)	BCL3 (19q13.32)	CDH11 (16q21)	DDIT3 (12q13.3)
ACSL3 (2q36.1)	BCL6 (3q27.3)	CDK12 (17q12)	DDR2 (1q23.3)
ACSL6 (5q31.1)	BCL7A (12q24.31)	CDK4 (12q14.1)	DDX10 (11q22.3)
ADGRA2 (8p11.23)	BCL9 (1q21.2)	CDK6 (7q21.2)	DDX5 (17q23.3)
AFDN (6q27)	BCOR (Xp11.4)	CDK8 (13q12.13)	DDX6 (11q23.3)
AFF1 (4q21.3-22.1)	BCORL1 (Xq26.1)	CDKN1B (12p13.1)	DEK (6p22.3)
AFF3 (2q11.2)	BCR (22q11.23)	CDKN2A (9p21.3)	DICER1 (14q32.13)
AFF4 (5q31.1)	BIRC3 (11q22.2)	CDKN2B (9p21.3)	DNM2 (19p13.2)
AKAP9 (7q21.2)	BLM (15q26.1)	CDKN2C (1p32.3)	DNMT3A (2p23.3)
AKT1 (14q32.33)	BMPR1A (10q23.2)	CDX2 (13q12.2)	DOT1L (19p13.3)
AKT2 (19q13.2)	BRAF (7q34)	CEBPA (19q13.11)	EBF1 (5q33.3)
AKT3 (1q43-44)	BRCA1 (17q21.31)	CHCHD7 (8q12.1)	ECT2L (6q24.1)
ALDH2 (12q24.12)	BRCA2 (13q13.1)	CHEK1 (11q24.2)	EGFR (7p11.2)
ALK (2p23.2-23.1)	BRD3 (9q34.2)	CHEK2 (22q12.1)	EIF4A2 (3q27.3)
AMER1 (Xq11.2)	BRD4 (19p13.12)	CHIC2 (4q12)	ELF4 (Xq26.1)
APC (5q22.2)	BRIP1 (17q23.2)	CHN1 (2q31.1)	ELK4 (1q32.1)
AR (Xq12)	BTG1 (12q21.33)	CIC (19q13.2)	ELL (19p13.11)
ARAF (Xp11.3)	BTK (Xq22.1)	CIITA (16p13.13)	ELN (7q11.23)
ARFRP1 (20q13.33)	BUB1B (15q15.1)	CLP1 (11q12.1)	EML4 (2p21)
ARHGAP26 (5q31.3)	C15orf65 (15q21.3)	CLTC (17q23.1)	EMSY (11q13.5)
ARHGEF12 (11q23.3)	CACNA1D (3p21.1)	CLTCL1 (22q11.21)	EP300 (22q13.2)
ARID1A (1p36.11)	CALR (19p13.13)	CNBP (3q21.3)	EPHA3 (3p11.1)
ARID2 (12q12)	CAMTA1 (1p36.31-36.23)	CNOT3 (19q13.42)	EPHA5 (4q13.1-13.2)
ARNT (1q21.3)	CANT1 (17q25.3)	CNTRL (9q33.2)	EPHB1 (3q22.2)
ASPSCR1 (17q25.3)	CARD11 (7p22.2)	COL1A1 (17q21.33)	EPS15 (1p32.3)
ASXL1 (20q11.21)	CARS1 (11p15.4)	COPB1 (11p15.2)	ERBB2 (17q12)
ATF1 (12q13.12)	CASP8 (2q33.1)	COX6C (8q22.2)	ERBB3 (12q13.2)
ATIC (2q35)	CBFA2T3 (16q24.3)	CREB1 (2q33.3)	ERBB4 (2q34)
ATM (11q22.3)	CBFB (16q22.1)	CREB3L1 (11p11.2)	ERC1 (12p13.33)
ATP1A1 (1p13.1)	CBL (11q23.3)	CREB3L2 (7q33)	ERCC1 (19q13.32)
ATP2B3 (Xq28)	CBLB (3q13.11)	CREBBP (16p13.3)	ERCC2 (19q13.32)
ATR (3q23)	CBLC (19q13.32)	CRKL (22q11.21)	ERCC3 (2q14.3)
ATRX (Xq21.1)	CCDC6 (10q21.2)	CRLF2 (Xp22.33)	ERCC4 (16p13.12)
AURKA (20q13.2)	CCN6 (6q21)	CRTC1 (19p13.11)	ERCC5 (13q33.1)
AURKB (17p13.1)	CCNB1IP1 (14q11.2)	CRTC3 (15q26.1)	ERG (21q22.2)
AXIN1 (16p13.3)	CCND1 (11q13.3)	CSF1R (5q32)	ESR1 (6q25.1-25.2)
AXL (19q13.2)	CCND2 (12p13.32)	CSF3R (1p34.3)	ETV1 (7p21.2)
BAP1 (3p21.1)	CCND3 (6p21.1)	CTCF (16q22.1)	ETV4 (17q21.31)
BARD1 (2q35)	CCNE1 (19q12)	CTLA4 (2q33.2)	ETV5 (3q27.2)
BCL10 (1p22.3)	CD274 (9p24.1)	CTNNA1 (5q31.2)	ETV6 (12p13.2)
BCL11A (2p16.1)	CD74 (5q33.1)	CTNNB1 (3p22.1)	EWSR1 (22q12.2)
BCL11B (14q32.2)	CD79A (19q13.2)	CYLD (16q12.1)	EXT1 (8q24.11)
BCL2 (18q21.33)	CD79B (17q23.3)	CYP2D6 (22q13.2)	EXT2 (11p11.2)

Supplementary Table 1, continued…			
EZH2 (7q36.1)	GATA1 (Xp11.23)	IL21R (16p12.1)	MAF (16q23.2)
EZR (6q25.3)	GATA2 (3q21.3)	IL6ST (5q11.2)	MAFB (20q12)
FANCA (16q24.3)	GATA3 (10p14)	IL7R (5p13.2)	MALT1 (18q21.32)
FANCC (9q22.32)	GID4 (17p11.2)	INHBA (7p14.1)	MAML2 (11q21)
FANCD2 (3p25.3)	GMPS (3q25.31)	IRF4 (6p25.3)	MAP2K1 (15q22.31)
FANCE (6p21.31)	GNA11 (19p13.3)	IRS2 (13q34)	MAP2K2 (19p13.3)
FANCF (11p14.3)	GNA13 (17q24.1)	ITK (5q33.3)	MAP2K4 (17p12)
FANCG (9p13.3)	GNAQ (9q21.2)	JAK1 (1p31.3)	MAP3K1 (5q11.2)
FANCL (2p16.1)	GNAS (20q13.32)	JAK2 (9p24.1)	MAX (14q23.3)
FAS (10q23.31)	GOLGA5 (14q32.12)	JAK3 (19p13.11)	MCL1 (1q21.2)
FBXO11 (2p16.3)	GOPC (6q22.1)	JAZF1 (7p15.2-15.1)	MDM2 (12q15)
FBXW7 (4q31.3)	GPC3 (Xq26.2)	JUN (1p32.1)	MDM4 (1q32.1)
FCRL4 (1q23.1)	GPHN (14q23.3-24.1)	KAT6A (8p11.21)	MDS2 (1p36.11)
FEV (2q35)	GRIN2A (16p13.2)	KAT6B (10q22.2)	MECOM (3q26.2)
FGF10 (5p12)	GSK3B (3q13.33)	KCNJ5 (11q24.3)	MED12 (Xq13.1)
FGF14 (13q33.1)	H3-3A (1q42.12)	KDM5A (12p13.33)	MEF2B (19p13.11)
FGF19 (11q13.3)	H3-3B (17q25.1)	KDM5C (Xp11.22)	MEN1 (11q13.1)
FGF23 (12p13.32)	H3C2 (6p22.2)	KDM6A (Xp11.3)	MET (7q31.2)
FGF3 (11q13.3)	H4C9 (6p22.1)	KDR (4q12)	MITF (3p13)
FGF4 (11q13.3)	HERPUD1 (16q13)	KDSR (18q21.33)	MLF1 (3q25.32)
FGF6 (12p13.32)	HEY1 (8q21.13)	KEAP1 (19p13.2)	MLH1 (3p22.2)
FGFR1 (8p11.23)	HGF (7q21.11)	KIAA1549 (7q34)	MLLT1 (19p13.3)
FGFR1OP (6q27)	HIP1 (7q11.23)	KIF5B (10p11.22)	MLLT10 (10p12.31)
FGFR2 (10q26.13)	HLF (17q22)	KIT (4q12)	MLLT11 (1q21.3)
FGFR3 (4p16.3)	HMGA1 (6p21.31)	KLF4 (9q31.2)	MLLT3 (9p21.3)
FGFR4 (5q35.2)	HMGA2 (12q14.3)	KLHL6 (3q27.1)	MLLT6 (17q12)
FH (1q43)	HNF1A (12q24.31)	KLK2 (19q13.33)	MN1 (22q12.1)
FHIT (3p14.2)	HNRNPA2B1 (7p15.2)	KMT2A (11q23.3)	MNX1 (7q36.3)
FIP1L1 (4q12)	HOOK3 (8p11.21)	KMT2C (7q36.1)	MPL (1p34.2)
FLCN (17p11.2)	HOXA11 (7p15.2)	KMT2D (12q13.12)	MRE11 (11q21)
FLI1 (11q24.3)	HOXA13 (7p15.2)	KNL1 (15q15.1)	MRTFA (22q13.1-13.2)
FLT1 (13q12.3)	HOXA9 (7p15.2)	KRAS (12p12.1)	MSH2 (2p21-16.3)
FLT3 (13q12.2)	HOXC11 (12q13.13)	KTN1 (14q22.3)	MSH6 (2p16.3)
FLT4 (5q35.3)	HOXC13 (12q13.13)	LASP1 (17q12)	MSI2 (17q22)
FNBP1 (9q34.11)	HOXD11 (2q31.1)	LCK (1p35.2)	MSN (Xq12)
FOXA1 (14q21.1)	HOXD13 (2q31.1)	LCP1 (13q14.13)	MTCP1 (Xq28)
FOXL2 (3q22.3)	HRAS (11p15.5)	LGR5 (12q21.1)	MTOR (1p36.22)
FOXO1 (13q14.11)	HSP90AA1 (14q32.31)	LHFPL6 (13q13.3-14.11)	MUC1 (1q22)
FOXO3 (6q21)	HSP90AB1 (6p21.1)	LIFR (5p13.1)	MUTYH (1p34.1)
FOXO4 (Xq13.1)	IDH1 (2q34)	LMO1 (11p15.4)	MYB (6q23.3)
FOXP1 (3p13)	IDH2 (15q26.1)	LMO2 (11p13)	MYC (8q24.21)
FSTL3 (19p13.3)	IGF1R (15q26.3)	LPP (3q27.3-28)	MYCL (1p34.2)
FUBP1 (1p31.1)	IKBKE (1q32.1)	LRIG3 (12q14.1)	MYCN (2p24.3)
FUS (16p11.2)	IKZF1 (7p12.2)	LRP1B (2q22.1-22.2)	MYD88 (3p22.2)
GAS7 (17p13.1)	IL2 (4q27)	LYL1 (19p13.13)	MYH11 (16p13.11)

Supplementary Table 1, continued…			
MYH9 (22q12.3)	PAX8 (2q14.1)	RAD21 (8q24.11)	SMAD2 (18q21.1)
NACA (12q13.3)	PBRM1 (3p21.1)	RAD50 (5q31.1)	SMAD4 (18q21.2)
NBN (8q21.3)	PBX1 (1q23.3)	RAD51 (15q15.1)	SMARCA4 (19p13.2)
NCKIPSD (3p21.31)	PCM1 (8p22)	RAD51B (14q24.1)	SMARCB1 (22q11.23)
NCOA1 (2p23.3)	PCSK7 (11q23.3)	RAF1 (3p25.2)	SMARCE1 (17q21.2)
NCOA2 (8q13.3)	PDCD1 (2q37.3)	RALGDS (9q34.13-34.2)	SMO (7q32.1)
NCOA4 (10q11.22)	PDCD1LG2 (9p24.1)	RANBP17 (5q35.1)	SNX29 (16p13.13-13.12)
NDRG1 (8q24.22)	PDE4DIP (1q21.2)	RAP1GDS1 (4q23)	SOCS1 (16p13.13)
NF1 (17q11.2)	PDGFB (22q13.1)	RARA (17q21.2)	SOX10 (22q13.1)
NF2 (22q12.2)	PDGFRA (4q12)	RB1 (13q14.2)	SOX2 (3q26.33)
NFE2L2 (2q31.2)	PDGFRB (5q32)	RBM15 (1p13.3)	SPECC1 (17p11.2)
NFIB (9p23-22.3)	PDK1 (2q31.1)	RECQL4 (8q24.3)	SPEN (1p36.21-36.13)
NFKB2 (10q24.32)	PER1 (17p13.1)	REL (2p16.1)	SPOP (17q21.33)
NFKBIA (14q13.2)	PHF6 (Xq26.2)	RET (10q11.21)	SRC (20q11.23)
NIN (14q22.1)	PHOX2B (4p13)	RHOH (4p14)	SRGAP3 (3p25.3)
NKX2-1 (14q13.3)	PICALM (11q14.2)	RICTOR (5p13.1)	SRSF2 (17q25.1)
NONO (Xq13.1)	PIK3CA (3q26.32)	RMI2 (16p13.13)	SRSF3 (6p21.31-21.2)
NOTCH1 (9q34.3)	PIK3CG (7q22.3)	RNF213 (17q25.3)	SS18 (18q11.2)
NOTCH2 (1p12)	PIK3R1 (5q13.1)	RNF43 (17q22)	SS18L1 (20q13.33)
NPM1 (5q35.1)	PIK3R2 (19p13.11)	ROS1 (6q22.1)	SSX1 (Xp11.23)
NR4A3 (9q31.1)	PIM1 (6p21.2)	RPL10 (Xq28)	STAG2 (Xq25)
NRAS (1p13.2)	PLAG1 (8q12.1)	RPL22 (1p36.31)	STAT3 (17q21.2)
NSD1 (5q35.3)	PML (15q24.1)	RPL5 (1p22.1)	STAT4 (2q32.2-32.3)
NSD2 (4p16.3)	PMS1 (2q32.2)	RPN1 (3q21.3)	STAT5B (17q21.2)
NSD3 (8p11.23)	PMS2 (7p22.1)	RPTOR (17q25.3)	STIL (1p33)
NT5C2 (10q24.32-24.33)	POLE (12q24.33)	RUNX1 (21q22.12)	STK11 (19p13.3)
NTRK1 (1q23.1)	POT1 (7q31.33)	RUNX1T1 (8q21.3)	SUFU (10q24.32)
NTRK2 (9q21.33)	POU2AF1 (11q23.1)	SBDS (7q11.21)	SUZ12 (17q11.2)
NTRK3 (15q25.3)	POU5F1 (6p21.33)	SDC4 (20q13.12)	SYK (9q22.2)
NUMA1 (11q13.4)	PPARG (3p25.2)	SDHAF2 (11q12.2)	TAF15 (17q12)
NUP214 (9q34.13)	PPP2R1A (19q13.41)	SDHB (1p36.13)	TAL1 (1p33)
NUP93 (16q13)	PRCC (1q23.1)	SDHC (1q23.3)	TAL2 (9q31.2)
NUP98 (11p15.4)	PRDM1 (6q21)	SDHD (11q23.1)	TBL1XR1 (3q26.32)
NUTM1 (15q14)	PRDM16 (1p36.32)	SEPTIN5 (22q11.21)	TCEA1 (8q11.23)
NUTM2B (10q22.3)	PRF1 (10q22.1)	SEPTIN6 (Xq24)	TCF12 (15q21.3)
OLIG2 (21q22.11)	PRKAR1A (17q24.2)	SEPTIN9 (17q25.3)	TCF3 (19p13.3)
OMD (9q22.31)	PRKDC (8q11.21)	SET (9q34.11)	TCF7L2 (10q25.2-25.3)
P2RY8 (Xp22.33)	PRRX1 (1q24.2)	SETBP1 (18q12.3)	TCL1A (14q32.13)
PAFAH1B2 (11q23.3)	PSIP1 (9p22.3)	SETD2 (3p21.31)	TENT5C (1p12)
PAK3 (Xq23)	PTCH1 (9q22.32)	SF3B1 (2q33.1)	TERT (5p15.33)
PALB2 (16p12.2)	PTEN (10q23.31)	SFPQ (1p34.3)	TET1 (10q21.3)
PATZ1 (22q12.2)	PTPN11 (12q24.13)	SH2B3 (12q24.12)	TET2 (4q24)
PAX3 (2q36.1)	PTPRC (1q31.3-32.1)	SH3GL1 (19p13.3)	TFE3 (Xp11.23)
PAX5 (9p13.2)	RABEP1 (17p13.2)	SLC34A2 (4p15.2)	TFEB (6p21.1)
PAX7 (1p36.13)	RAC1 (7p22.1)	SLC45A3 (1q32.1)	TFG (3q12.2)

Supplementary Table 1, continued…	
TFPT (19q13.42)	ZNF331 (19q13.42)
TFRC (3q29)	ZNF384 (12p13.31)
TGFBR2 (3p24.1)	ZNF521 (18q11.2)
THRAP3 (1p34.3)	ZNF703 (8p11.23)
TLX1 (10q24.31)	ZRSR2 (Xp22.2)
TLX3 (5q35.1)	
TMPRSS2 (21q22.3)	
TNFAIP3 (6q23.3)	
TNFRSF14 (1p36.32)	
TNFRSF17 (16p13.13)	
TOP1 (20q12)	
TP53 (17p13.1)	
TPM3 (1q21.3)	
TPM4 (19p13.12-13.11)	
TPR (1q31.1)	
TRAF7 (16p13.3)	
TRIM26 (6p22.1)	
TRIM27 (6p22.1)	
TRIM33 (1p13.2)	
TRIP11 (14q32.12)	
TRRAP (7q22.1)	
TSC1 (9q34.13)	
TSC2 (16p13.3)	
TSHR (14q31.1)	
TTL (2q14.1)	
U2AF1 (21q22.3)	
UBR5 (8q22.3)	
USP6 (17p13.2)	
VEGFA (6p21.1)	
VEGFB (11q13.1)	
VHL (3p25.3)	
VTI1A (10q25.2)	
WAS (Xp11.23)	
WDCP (2p23.3)	
WIF1 (12q14.3)	
WRN (8p12)	
WT1 (11p13)	
WWTR1 (3q25.1)	
XPA (9q22.33)	
XPC (3p25.1)	
XPO1 (2p15)	
YWHAE (17p13.3)	
ZBTB16 (11q23.2)	
ZMYM2 (13q12.11)	
ZNF217 (20q13.2)	

Supplementary Data 2
Model Variables

Variable	Category	Type	Source	Description
Response	clinical	categorical		levels: CR/PR, SD/PD
Age	clinical	continuous		
BCG	clinical	categorical		prior BCG treatment
Consensus_subtype_BaSq	clinical	categorical	Kamoun 2020	basal/squamous
Consensus_subtype_LumP	clinical	categorical	Kamoun 2020	luminal papillary
Consensus_subtype_LumU	clinical	categorical	Kamoun 2020	luminal unstable
Consensus_subtype_StromaRich<br>h	clinical	categorical	Kamoun 2020	stroma-rich
ECOG_0	clinical	categorical	Oken 1982	ECOG performance status = 0
ECOG_2plus	clinical	categorical	Oken 1982	ECOG performance status ≥ 2
Prior_platinum	clinical	categorical		prior platinum therapy
Sex	clinical	categorical		levels: male, female
Tobacco	clinical	categorical		levels: never, previous, current
Ayers_IFNG	immunogenomic	continuous	Ayers 2017	
Ayers_T_cell_inflamed_GEP	immunogenomic	continuous	Ayers 2017	
B_Cell	immunogenomic	continuous	Iglesia 2014	
Bindea_aDC	immunogenomic	continuous	Bindea 2013	activated dendritic cell
Bindea_B_cells	immunogenomic	continuous	Bindea 2013	
Bindea_CD8_T_cells	immunogenomic	continuous	Bindea 2013	
Bindea_Cytotoxic_cells	immunogenomic	continuous	Bindea 2013	
Bindea_DC	immunogenomic	continuous	Bindea 2013	
Bindea_Eosinophils	immunogenomic	continuous	Bindea 2013	
Bindea_iDC	immunogenomic	continuous	Bindea 2013	inactivated dendritic cell
Bindea_Neutrophils	immunogenomic	continuous	Bindea 2013	
Bindea_NK_CD56bright_cells	immunogenomic	continuous	Bindea 2013	

Supplementary Data 2
Model Variables

Variable	Category	Type	Source	Description
Bindea_NK_CD56dim_cells	immunogenomic	continuous	Bindea 2013	
Bindea_NK_cells	immunogenomic	continuous	Bindea 2013	
Bindea_pDC	immunogenomic	continuous	Bindea 2013	plasmacytoid dendritic cell
Bindea_T_cells	immunogenomic	continuous	Bindea 2013	
Bindea_T_helper_cells	immunogenomic	continuous	Bindea 2013	
Bindea_Tcm	immunogenomic	continuous	Bindea 2013	central memory T cell
Bindea_Tem	immunogenomic	continuous	Bindea 2013	effector memory T cell
Bindea_TFH	immunogenomic	continuous	Bindea 2013	follicular helper T cell
Bindea_Tgd	immunogenomic	continuous	Bindea 2013	gamma-delta T cell
Bindea_Th1_cells	immunogenomic	continuous	Bindea 2013	
Bindea_Th2_cells	immunogenomic	continuous	Bindea 2013	
BIOCARTA_TGFB_Pathway	immunogenomic	continuous	BioCarta	
Byers_EMT CD8	immunogenomic	continuous	Byers 2013	epithelial-mesenchymal
Claudin EMT_DOWN	immunogenomic	continuous	Iglesia 2014	transition
EMT_Stroma_core_18	immunogenomic	continuous	Prat 2010	CDLN3, CLDN4, CLDN7
EMT_Stroma_core_8 EMT_UP	immunogenomic	continuous	Hayashi 2016	pan-fibroblast TGF-β response
FTBRS	immunogenomic	continuous	Wang 2018	T cell receptor signaling GO
	immunogenomic	continuous	Wang 2018	term
	immunogenomic	continuous	Hayashi 2016 Mariathasan	B cell receptor signaling GO
	immunogenomic	continuous	2018	term
				tumor mutational burden
				tumor mutational burden > 10
				AML pembrolizumab non-
				responder
				AML pembrolizumab
				responder
GO_BCR_signaling	immunogenomic	continuous	Iglesia 2014	

Supplementary Data 2
Model Variables

Variable	Category	Type	Source
GO_TCR_signaling Hollern_Bcell	immunogenomic	continuous	Iglesia 2014
Hollern_Tcell Iglesia_IGG_Cluster	immunogenomic	continuous	Hollern 2019
Iglesia_MacTh1_Cluster	immunogenomic	continuous	Hollern 2019
Iglesia_T_Cell_Cluster IL_8	immunogenomic	continuous	Iglesia 2014
Immunosuppression LCK	immunogenomic	continuous	Iglesia 2014
Mac_CSF1 Macrophages	immunogenomic	continuous	Iglesia 2014
Martinez_Gordon_M1	immunogenomic	continuous	Iglesia 2014
Martinez_Gordon_M2 Mast_cells	immunogenomic	continuous	Kardos 2016
McDermott_T_eff Murray_M1	immunogenomic	continuous	Iglesia 2014
Murray_M2 T_Cell TCGA_IFNG	immunogenomic	continuous	Iglesia 2014
TIC	immunogenomic	continuous	Bindea 2013
	immunogenomic	continuous	Martinez 2014
	immunogenomic	continuous	Martinez 2014
	immunogenomic	continuous	Bindea 2013 McDermott
	immunogenomic	continuous	2018
	immunogenomic	continuous	Murray 2014
	immunogenomic	continuous	Murray 2014
	immunogenomic	continuous	Iglesia 2014
	immunogenomic	continuous	Thorsson 2018
	immunogenomic	continuous	Chan 2009
TMB	immunogenomic	continuous	
TMB_high TNBC_B_Cell	immunogenomic	categorical	Iglesia 2014
	immunogenomic	continuous	
Vincent_IPRES_NonResponder	immunogenomic	continuous	Zeidner 2018
Vincent_IPRES_Responder	immunogenomic	continuous	Zeidner 2018
