# Supplementary material for: Collaborative study from the Bladder Cancer Advocacy Network for the genomic analysis of metastatic urothelial cancer
Source: Nat Commun. 2022 Nov 4;13:6658. doi: 10.1038/s41467-022-33980-9 (PMC9636269; doi:10.1038/s41467-022-33980-9)
Supplement: Supplementary file 2 — Description to Additional Supplementary Information [file 41467_2022_33980_MOESM2_ESM.pdf]

Description of additional Supplementary Files

Supplementary Data 1: Gene included in the Caris 592 gene targeted sequencing panel

Supplementary Data 2: Elastic net model variables
