# Supplementary material for: Collaborative study from the Bladder Cancer Advocacy Network for the genomic analysis of metastatic urothelial cancer
Source: Nat Commun. 2022 Nov 4;13:6658. doi: 10.1038/s41467-022-33980-9 (PMC9636269; doi:10.1038/s41467-022-33980-9)
Supplement: Supplementary file 5 — Reporting Summary [file 41467_2022_33980_MOESM5_ESM.pdf]

Corresponding author(s): Kim and Milowsky

Last updated by author(s): 10/25/2022

Reporting Summary

Nature Portfolio wishes to improve the reproducibility of the work that we publish. This form provides structure for consistency and transparency in reporting. For further information on Nature Portfolio policies, see our Editorial Policies and the Editorial Policy Checklist.

Statistics

For all statistical analyses, confirm that the following items are present in the figure legend, table legend, main text, or Methods section.

n/a	Confirmed	
☐	☒	The exact sample size (*n*) for each experimental group/condition, given as a discrete number and unit of measurement
☐	☒	A statement on whether measurements were taken from distinct samples or whether the same sample was measured repeatedly
☐	☒	The statistical test(s) used AND whether they are one- or two-sided<br>*Only common tests should be described solely by name; describe more complex techniques in the Methods section.*
☐	☒	A description of all covariates tested
☐	☒	A description of any assumptions or corrections, such as tests of normality and adjustment for multiple comparisons
☐	☒	A full description of the statistical parameters including central tendency (e.g. means) or other basic estimates (e.g. regression coefficient) AND variation (e.g. standard deviation) or associated estimates of uncertainty (e.g. confidence intervals)
☐	☒	For null hypothesis testing, the test statistic (e.g. *F*, *t*, *r*) with confidence intervals, effect sizes, degrees of freedom and *P* value noted<br>*Give P values as exact values whenever suitable.*
☐	☒	For Bayesian analysis, information on the choice of priors and Markov chain Monte Carlo settings
☐	☒	For hierarchical and complex designs, identification of the appropriate level for tests and full reporting of outcomes
☐	☒	Estimates of effect sizes (e.g. Cohen's *d*, Pearson's *r*), indicating how they were calculated

Our web collection on statistics for biologists contains articles on many of the points above.

Software and code

Policy information about availability of computer code

Data collection	RNAseq reads were aligned using STAR 2.7.2a and quantified using Salmon v1.0.0. DNA seq was performed and variants called by Caris Life Science.
Data analysis	A R packages have been previously published for R version 4.1.1. Packages included: biomaRt 2.52.0, BLCAsubtypingv2.1, SomaticSignatures 2.32.0, Consensus Cluster Plus (CCP) 1.60.0, CIBERSORTx, MiXCR v3.0.13, tcRv2.x, caret 6.0-92

For manuscripts utilizing custom algorithms or software that are central to the research but not yet described in published literature, software must be made available to editors and reviewers. We strongly encourage code deposition in a community repository (e.g. GitHub). See the Nature Portfolio guidelines for submitting code & software for further information.

Data

Policy information about availability of data

All manuscripts must include a data availability statement. This statement should provide the following information, where applicable:

- Accession codes, unique identifiers, or web links for publicly available datasets
- A description of any restrictions on data availability
- For clinical datasets or third party data, please ensure that the statement adheres to our policy

The UC-GENOME mutation annotation file (MAF), gene expression matrix and clinical data generated in this study have been deposited in the cBioPortal database under “Urothelial Carcinoma, Nature Communications (BCAN/HCRN 2022)” [https://www.cbioportal.org/study/summary?id=blca_bcan_hcrn_2022]. All RNA and DNA FASTQ files are available on dbGaP, phs003066.v1.p1 [https://www.ncbi.nlm.nih.gov/projects/gap/cgi-bin/study.cgi?study_id=phs003066.v1.p1] under restricted access for disease specific research, with the exception of samples obtained from Johns Hopkins University (JHU). The JHU samples are available, are only available to investigators from non-profit entities with an IRB conducting disease specific research, dbGaP study ID phs003094.v1.p1, [https://

www.ncbi.nlm.nih.gov/projects/gap/cgi-bin/study.cgi?study_id=phs003094.v1.p1]. The data used to generate the figures in this study as well as the code for the elastic net classifier are provided in the Supplementary Information/Source Data file and also via FigShare.com [https://figshare.com/articles/journal_contribution/UC-GENOME/19491287]. Upper quartile normalized RSEM gene expression data and DNA sequencing data for TCGA was downloaded from the GDC legacy archive [https://portal.gdc.cancer.gov/legacy-archive/]. IMvigor210 data was downloaded from the European Genome-Phenome Archive, EGAS00001004343. UNC-108 RNA sequencing data was obtained from GEO, GSE176307 and DNA sequencing data through request of the authors.study_id=phs003094.v1.p1]. The data used to generate the figures in this study as well as the code for the elastic net classifier are provided in the Supplementary Information/Source Data file and also via FigShare.com [https://figshare.com/articles/journal_contribution/UC-GENOME/19491287]. Upper quartile normalized RSEM gene expression data and DNA sequencing data for TCGA was downloaded from the GDC legacy archive [https://portal.gdc.cancer.gov/legacy-archive/]. IMvigor210 data was downloaded from the European Genome-Phenome Archive, EGAS00001004343 [https://ega-archive.org/studies/EGAS00001004343]. UNC-108 RNA sequencing data was obtained from GEO, GSE176307 [https://www.ncbi.nlm.nih.gov/geo/query/acc.cgi?acc=GSE176307] and DNA sequencing data through request of the authors.

Field-specific reporting

Please select the one below that is the best fit for your research. If you are not sure, read the appropriate sections before making your selection.

☒ Life sciences ☐ Behavioural & social sciences ☐ Ecological, evolutionary & environmental sciences

For a reference copy of the document with all sections, see nature.com/documents/nr-reporting-summary-flat.pdf

Life sciences study design

All studies must disclose on these points even when the disclosure is negative.

Sample size: Sample size was determined by the number of patients that were enrolled on NCT02643043 and had successful RNA and DNA sequencing.

Data exclusions: No data was excluded

Replication: Metastatic (IMvigor210, UNC-108) and non-metastatic cohorts (TCGA, Kamoun) were used to validate/compare the findings of the primary dataset. For proportion of subtype and mutation frequency analysis, independent analysis of the datasets were performed and the findings were compared to that of the primary dataset. The principle findings of our study were successfully validated in the metastatic cohorts, while diverged from the non-metastatic cohorts. Additionally, in the elastic net model, the IMvigor210 dataset was divided into a training and validation cohort, and further successfully validated against UNC-108 and UC-GENOME.

Randomization: This is not relevant to the study as there was no randomization.

Blinding: As this was the foundational analysis for a metastatic bladder cancer dataset, with one of the objectives being to provide a NGS report back to the patient's physician, blinding was not necessary as there was no subject allocation.

Reporting for specific materials, systems and methods

We require information from authors about some types of materials, experimental systems and methods used in many studies. Here, indicate whether each material, system or method listed is relevant to your study. If you are not sure if a list item applies to your research, read the appropriate section before selecting a response.

Materials & experimental systems

n/a	Involved in the study	
☐	☒	Antibodies
☒	☐	Eukaryotic cell lines
☒	☐	Palaeontology and archaeology
☒	☐	Animals and other organisms
☐	☒	Human research participants
☐	☒	Clinical data
☒	☐	Dual use research of concern

Methods

n/a	Involved in the study	
☒	☐	ChIP-seq
☒	☐	Flow cytometry
☒	☐	MRI-based neuroimaging

Antibodies

Antibodies used: CD8 (SP16) Rabbit Monoclonal Antibody, Vendor: Cell Marque, catalogue #108R-16

Validation: The antibody has been validated per the description on the vendors website: https://www.cellmarque.com/antibodies/CM/2102/CD8_SP16
As part of this study the UNC Pathology Service Core has validated the antibody on human benign tonsil.

Human research participants

Policy information about studies involving human research participants

Population characteristics	All patients were at least 18 years of age (28-85 yo) and had been diagnosed with metastatic urothelial cancer of the bladder, urethra, ureter, or renal pelvis. The patients were predominately Male (75%) and white (79%), with current/former smokers comprising 68% of the cohort. A majority of the patients (86%) received a systemic therapy for metastatic disease.
Recruitment	Patients were recruited in the medical oncology clinics with PIs at their respective institutions that participated in the study (sites included: University of North Carolina at Chapel Hill, Fox Chase Cancer Center, Icahn School of Medicine at Mount Sinai, University of Washington/ Fred Hutchinson Cancer Research Center, Johns Hopkins University, University of Chicago, Memorial Sloan Kettering Cancer Center, and University of Southern California). To be enrolled in the study, patients were required to have tumor tissue available and suitable for molecular analysis, therefore patients who did not have enough tumor available could not enroll. This may have biased the study toward higher volume disease. Additionally, the patients were recruited by physicians at one of the contributing institutions all of which are major academic sites, biasing the population to those with the means and ability to make receive care at these sites.
Ethics oversight	The IRBs of the following contributing institutions approved this study: University of North Carolina at Chapel Hill, Fox Chase Cancer Center, Icahn School of Medicine at Mount Sinai, University of Washington/ Fred Hutchinson Cancer Research Center, Johns Hopkins University, University of Chicago, Memorial Sloan Kettering Cancer Center, and University of Southern California. All samples and clinical and molecular data were de-identified with The Hoosier Cancer Research Network serving as the honest broker.

Note that full information on the approval of the study protocol must also be provided in the manuscript.

Clinical data

Policy information about clinical studies

All manuscripts should comply with the ICMJE guidelines for publication of clinical research and a completed CONSORT checklist must be included with all submissions.

Clinical trial registration	NCT02643043
Study protocol	https://clinicaltrials.gov/ct2/show/NCT02643043
Data collection	Accrual started 7/2016 and last until 5/2019. Data collection occurred between 7/2019 and 1/2020. Patients were enrolled at the following sites: University of North Carolina at Chapel Hill, Fox Chase Cancer Center, Icahn School of Medicine at Mount Sinai, University of Washington/ Fred Hutchinson Cancer Research Center, Johns Hopkins University, University of Chicago, Memorial Sloan Kettering Cancer Center, and University of Southern California (participating site with no accruals).
Outcomes	Primary Objectives<br>1) Estimate the proportion of subjects with metastatic UC enrolled who receive NGS and have a personalized report generated with potential treatment options.<br>2) Create a biospecimen and data repository by collecting and storing blood and archival tumor tissue (biospecimens) from subjects and linking molecular and biological information from those biospecimens to clinical data in order to promote future translational research in metastatic UC.<br><br>Secondary Objectives<br>1) Estimate the proportion of subjects whose personalized report includes targeted therapy options (approved or investigational drugs).<br>2) Estimate the proportion of subjects who enroll in a clinical trial of targeted therapy based on NGS results.<br>3) Estimate the proportion of subjects who receive targeted therapy (outside of a clinical trial) based on NGS results.<br>4) Describe the demographics, treatment history and outcomes for subjects enrolled in the study.<br>5) Document the number and type of clinical trials and basic/translational science or other research projects based on the biospecimen and data repository.
