# Supplementary material for: Collaborative study from the Bladder Cancer Advocacy Network for the genomic analysis of metastatic urothelial cancer
Source: Nat Commun. 2022 Nov 4;13:6658. doi: 10.1038/s41467-022-33980-9 (PMC9636269; doi:10.1038/s41467-022-33980-9)
Supplement: Supplementary file 6 — Source_Data_Files [file 41467_2022_33980_MOESM6_ESM.zip › Source_Data_Files/Fig.7_Sup.Fig.7_ElasticNet_Modeling_Data_Code.zip!/ElasticNet Modeling Data and Code/ElasticNet.Modeling.html]

ElasticNet Modeling


# ElasticNet Modeling

#### Damrauer, Beck, et. al.

#### 4/26/2022

# ElasticNet (EN) modeling for Immune checkpoint inhibition (ICI) response (Damrauer, Beck, et. al., 2022)

The below reference code was used to generate the EN model for prediction of response to ICI and their associated figure

# Data processing and model training (Training and Test set)

```
## 76 x 1 sparse Matrix of class "dgCMatrix"
##                                        s1
## (Intercept)                 -1.9147502247
## SexM                         .           
## Tobacco.Use.HistoryNEVER     .           
## Tobacco.Use.HistoryPREVIOUS  0.0641210998
## B_Cell                       .           
## Immunosuppression            .           
## LCK                          .           
## Mac_CSF1                     .           
## T_Cell                       .           
## TIC                          .           
## TNBC_B_Cell                  .           
## TNBC_T_Cell                  .           
## Iglesia_B_Cell_cluster       .           
## Iglesia_CD8_cluster          .           
## Iglesia_CD68_cluster         .           
## BCell_60gene                -0.1268971765
## Iglesia_IGG_Cluster         -0.0028901818
## Iglesia_MacTh1_cluster       .           
## Iglesia_T_Cell_cluster       .           
## Bindea_aDC                   0.0005844761
## Bindea_B_cells               .           
## Bindea_CD8_T_cells           .           
## Bindea_Cytotoxic_cells       .           
## Bindea_DC                   -0.1002538216
## Bindea_Eosinophils          -0.0671420797
## Bindea_iDC                  -0.0333978818
## CD8                          .           
## Macrophages                  .           
## Mast_cells                   .           
## Bindea_Neutrophils           .           
## Bindea_NK_CD56bright_cells   0.1294889533
## Bindea_NK_CD56dim_cells      .           
## Bindea_NK_cells              .           
## Bindea_pDC                  -0.0717792905
## Bindea_T_cells               .           
## Bindea_T_helper_cells        .           
## Bindea_Tcm                   .           
## Claudin                     -0.0885909364
## Bindea_Tem                   .           
## Bindea_TFH                   .           
## Bindea_Tgd                   0.1301467797
## Bindea_Th1_cells             .           
## Bindea_Th2_cells             0.2285375017
## Byers_EMT                    .           
## BIOCARTA_TGFB_Pathway        .           
## Martinez_Gordon_M1           0.2547340014
## Martinez_Gordon_M2           .           
## Murray_M1                    .           
## EMT_DOWN                    -0.0208950610
## Murray_M2                   -0.0693702172
## EMT_UP                       .           
## GO_BCR_signaling             .           
## GO_TCR_signaling             .           
## IL_8                         .           
## McDermott_T_eff              0.0348553892
## FTBRS                        .           
## EMT_Stroma_core_8            .           
## EMT_Stroma_core_18           .           
## TCGA_IFNG                    .           
## Ayers_IFNG                   0.1171107377
## Ayers_T_cell_inflamed_GEP    .           
## Vincent_IPRES_Responder      0.0280096097
## Vincent_IPRES_NonResponder   .           
## Hollern_Tcell                .           
## Hollern_Bcell                .           
## TMB.Numeric                  0.6487074217
## TMB.HighTRUE                 0.1617875511
## BCG                          .           
## ECOG.0                       0.3930607008
## ECOG.2plus                   .           
## prior.platinum               .           
## age                          0.2271380497
## consensusClass.BaSq          .           
## consensusClass.LumP          0.0446597938
## consensusClass.LumU         -0.0013722301
## consensusClass.StromaRich   -0.1795813719
```

## Final Model

## Test set validation

```
## $A
## [1] 0.8557331
## 
## $n.total
## [1] 104
## 
## $n.events
## [1] 28
## 
## $n.noevents
## [1] 76
## 
## $p.value
## [1] 1.478639e-08
```

```
## 
##  DeLong's test for two correlated ROC curves
## 
## data:  roc1 and roc2
## Z = 2.0752, p-value = 0.03797
## alternative hypothesis: true difference in AUC is not equal to 0
## 95 percent confidence interval:
##  0.009216638 0.322829694
## sample estimates:
## AUC of roc1 AUC of roc2 
##   0.8436293   0.6776062
```

## Independant Validation on UNC-108

## Model performance in UC-GENOME

```
## [1] 88
```

```
## $A
## [1] 0.6569479
## 
## $n.total
## [1] 83
## 
## $n.events
## [1] 31
## 
## $n.noevents
## [1] 52
## 
## $p.value
## [1] 0.008726958
```

## ROCs comparing UC-GENOME/IMvigor210/UNC-108

## Sup. 7: Plot of the training set model

## Sup. 7: Plots of density and glm (IMVigor210)

## Sup.7: plots for the model/training set

## Sup. 7: Plots of density and glm (UNC-108)

```
##  [1] "B_Cell"                     "Immunosuppression"         
##  [3] "LCK"                        "Mac_CSF1"                  
##  [5] "T_Cell"                     "TIC"                       
##  [7] "TNBC_B_Cell"                "TNBC_T_Cell"               
##  [9] "Iglesia_B_Cell_cluster"     "Iglesia_CD8_cluster"       
## [11] "Iglesia_CD68_cluster"       "BCell_60gene"              
## [13] "Iglesia_IGG_Cluster"        "Iglesia_MacTh1_cluster"    
## [15] "Iglesia_T_Cell_cluster"     "Bindea_aDC"                
## [17] "Bindea_B_cells"             "Bindea_CD8_T_cells"        
## [19] "Bindea_Cytotoxic_cells"     "Bindea_DC"                 
## [21] "Bindea_Eosinophils"         "Bindea_iDC"                
## [23] "CD8"                        "Macrophages"               
## [25] "Mast_cells"                 "Bindea_Neutrophils"        
## [27] "Bindea_NK_CD56bright_cells" "Bindea_NK_CD56dim_cells"   
## [29] "Bindea_NK_cells"            "Bindea_pDC"                
## [31] "Bindea_T_cells"             "Bindea_T_helper_cells"     
## [33] "Bindea_Tcm"                 "Claudin"                   
## [35] "Bindea_Tem"                 "Bindea_TFH"                
## [37] "Bindea_Tgd"                 "Bindea_Th1_cells"          
## [39] "Bindea_Th2_cells"           "Byers_EMT"                 
## [41] "BIOCARTA_TGFB_Pathway"      "Martinez_Gordon_M1"        
## [43] "Martinez_Gordon_M2"         "Murray_M1"                 
## [45] "EMT_DOWN"                   "Murray_M2"                 
## [47] "EMT_UP"                     "GO_BCR_signaling"          
## [49] "GO_TCR_signaling"           "IL_8"                      
## [51] "McDermott_T_eff"            "FTBRS"                     
## [53] "EMT_Stroma_core_8"          "EMT_Stroma_core_18"        
## [55] "TCGA_IFNG"                  "Ayers_IFNG"                
## [57] "Ayers_T_cell_inflamed_GEP"  "Vincent_IPRES_Responder"   
## [59] "Vincent_IPRES_NonResponder" "Hollern_Tcell"             
## [61] "Hollern_Bcell"              "Consensus"                 
## [63] "Age"                        "Smoking.History"           
## [65] "TMB"                        "TMB.high"                  
## [67] "prevplatinum"               "ECOG.0"                    
## [69] "ECOG.2plus"                 "binaryResponse"
```

## Sup. 7: ROCs for various prediction methods

```
##  [1] "X"                          "Sex"                       
##  [3] "Race"                       "Tobacco.Use.History"       
##  [5] "Met.Disease.Status"         "Tissue"                    
##  [7] "B_Cell"                     "Immunosuppression"         
##  [9] "LCK"                        "Mac_CSF1"                  
## [11] "T_Cell"                     "TIC"                       
## [13] "TNBC_B_Cell"                "TNBC_T_Cell"               
## [15] "Iglesia_B_Cell_cluster"     "Iglesia_CD8_cluster"       
## [17] "Iglesia_CD68_cluster"       "BCell_60gene"              
## [19] "Iglesia_IGG_Cluster"        "Iglesia_MacTh1_cluster"    
## [21] "Iglesia_T_Cell_cluster"     "Bindea_aDC"                
## [23] "Bindea_B_cells"             "Bindea_CD8_T_cells"        
## [25] "Bindea_Cytotoxic_cells"     "Bindea_DC"                 
## [27] "Bindea_Eosinophils"         "Bindea_iDC"                
## [29] "CD8"                        "Macrophages"               
## [31] "Mast_cells"                 "Bindea_Neutrophils"        
## [33] "Bindea_NK_CD56bright_cells" "Bindea_NK_CD56dim_cells"   
## [35] "Bindea_NK_cells"            "Bindea_pDC"                
## [37] "Bindea_T_cells"             "Bindea_T_helper_cells"     
## [39] "Bindea_Tcm"                 "Claudin"                   
## [41] "Bindea_Tem"                 "Bindea_TFH"                
## [43] "Bindea_Tgd"                 "Bindea_Th1_cells"          
## [45] "Bindea_Th2_cells"           "Byers_EMT"                 
## [47] "BIOCARTA_TGFB_Pathway"      "Martinez_Gordon_M1"        
## [49] "Martinez_Gordon_M2"         "Murray_M1"                 
## [51] "EMT_DOWN"                   "Murray_M2"                 
## [53] "EMT_UP"                     "GO_BCR_signaling"          
## [55] "GO_TCR_signaling"           "IL_8"                      
## [57] "McDermott_T_eff"            "FTBRS"                     
## [59] "EMT_Stroma_core_8"          "EMT_Stroma_core_18"        
## [61] "TCGA_IFNG"                  "Ayers_IFNG"                
## [63] "Ayers_T_cell_inflamed_GEP"  "Vincent_IPRES_Responder"   
## [65] "Vincent_IPRES_NonResponder" "Hollern_Tcell"             
## [67] "Hollern_Bcell"              "AKAP9"                     
## [69] "ARID1A"                     "RNF213"                    
## [71] "ERCC2"                      "TP53"                      
## [73] "KMT2D"                      "KMT2C"                     
## [75] "LRP1B"                      "SPEN"                      
## [77] "KDM6A"                      "BRCA2"                     
## [79] "CDKN2A"                     "TMB.Numeric"               
## [81] "TMB.High"                   "BCG"                       
## [83] "ECOG.0"                     "ECOG.2plus"                
## [85] "prior.platinum"             "age"                       
## [87] "consensusClass.BaSq"        "consensusClass.LumP"       
## [89] "consensusClass.LumU"        "consensusClass.StromaRich" 
## [91] "binaryResponse"
```

```
##   [1] "PREVIOUS" "PREVIOUS" "PREVIOUS" "PREVIOUS" "PREVIOUS" "PREVIOUS"
##   [7] "NEVER"    "NEVER"    "CURRENT"  "PREVIOUS" "NEVER"    "PREVIOUS"
##  [13] "PREVIOUS" "PREVIOUS" "NEVER"    "PREVIOUS" "PREVIOUS" "PREVIOUS"
##  [19] "PREVIOUS" "PREVIOUS" "PREVIOUS" "PREVIOUS" "PREVIOUS" "PREVIOUS"
##  [25] "PREVIOUS" "CURRENT"  "CURRENT"  "CURRENT"  "PREVIOUS" "PREVIOUS"
##  [31] "PREVIOUS" "NEVER"    "CURRENT"  "NEVER"    "PREVIOUS" "NEVER"   
##  [37] "PREVIOUS" "PREVIOUS" "NEVER"    "PREVIOUS" "NEVER"    "PREVIOUS"
##  [43] "PREVIOUS" "NEVER"    "PREVIOUS" "PREVIOUS" "NEVER"    "PREVIOUS"
##  [49] "NEVER"    "PREVIOUS" "PREVIOUS" "PREVIOUS" "PREVIOUS" "PREVIOUS"
##  [55] "NEVER"    "NEVER"    "NEVER"    "NEVER"    "PREVIOUS" "NEVER"   
##  [61] "NEVER"    "NEVER"    "NEVER"    "PREVIOUS" "CURRENT"  "PREVIOUS"
##  [67] "PREVIOUS" "PREVIOUS" "CURRENT"  "CURRENT"  "PREVIOUS" "NEVER"   
##  [73] "PREVIOUS" "PREVIOUS" "PREVIOUS" "NEVER"    "PREVIOUS" "NEVER"   
##  [79] "NEVER"    "PREVIOUS" "CURRENT"  "PREVIOUS" "PREVIOUS" "NEVER"   
##  [85] "NEVER"    "CURRENT"  "NEVER"    "PREVIOUS" "PREVIOUS" "NEVER"   
##  [91] "NEVER"    "NEVER"    "NEVER"    "NEVER"    "PREVIOUS" "NEVER"   
##  [97] "PREVIOUS" "CURRENT"  "NEVER"    "PREVIOUS" "PREVIOUS" "PREVIOUS"
## [103] "PREVIOUS" "PREVIOUS" "PREVIOUS" "PREVIOUS" "NEVER"    "PREVIOUS"
## [109] "PREVIOUS" "NEVER"    "PREVIOUS" "NEVER"    "PREVIOUS" "PREVIOUS"
## [115] "CURRENT"  "NEVER"    "PREVIOUS" "NEVER"    "PREVIOUS" "PREVIOUS"
## [121] "CURRENT"  "NEVER"    "NEVER"    "PREVIOUS" "NEVER"    "PREVIOUS"
## [127] "NEVER"    "PREVIOUS" "PREVIOUS" "NEVER"    "PREVIOUS" "PREVIOUS"
## [133] "PREVIOUS" "PREVIOUS" "PREVIOUS" "PREVIOUS" "CURRENT"  "CURRENT" 
## [139] "PREVIOUS" "NEVER"    "PREVIOUS" "PREVIOUS" "NEVER"    "PREVIOUS"
## [145] "PREVIOUS" "PREVIOUS" "PREVIOUS" "PREVIOUS" "PREVIOUS" "NEVER"   
## [151] "PREVIOUS" "PREVIOUS" "NEVER"    "PREVIOUS" "NEVER"
```

```
##        
##         FALSE TRUE
##   FALSE    76   37
##   TRUE     28   14
```

```
## $A
## [1] 0.7260338
## 
## $n.total
## [1] 104
## 
## $n.events
## [1] 28
## 
## $n.noevents
## [1] 76
## 
## $p.value
## [1] 6.517744e-06
```

```
## Warning in wilcox.test.default(pred[obs == 1], pred[obs == 0], alternative =
## "great"): cannot compute exact p-value with ties
```

```
## $A
## [1] 0.7084942
## 
## $n.total
## [1] 51
## 
## $n.events
## [1] 14
## 
## $n.noevents
## [1] 37
## 
## $p.value
## [1] 0.003580271
```

```
## [10:25:13] WARNING: amalgamation/../src/c_api/c_api.cc:718: `ntree_limit` is deprecated, use `iteration_range` instead.
## [10:25:13] WARNING: amalgamation/../src/c_api/c_api.cc:718: `ntree_limit` is deprecated, use `iteration_range` instead.
## [10:25:13] WARNING: amalgamation/../src/c_api/c_api.cc:718: `ntree_limit` is deprecated, use `iteration_range` instead.
## [10:25:13] WARNING: amalgamation/../src/c_api/c_api.cc:718: `ntree_limit` is deprecated, use `iteration_range` instead.
## [10:25:13] WARNING: amalgamation/../src/c_api/c_api.cc:718: `ntree_limit` is deprecated, use `iteration_range` instead.
## [10:25:13] WARNING: amalgamation/../src/c_api/c_api.cc:718: `ntree_limit` is deprecated, use `iteration_range` instead.
## [10:25:13] WARNING: amalgamation/../src/c_api/c_api.cc:718: `ntree_limit` is deprecated, use `iteration_range` instead.
## [10:25:13] WARNING: amalgamation/../src/c_api/c_api.cc:718: `ntree_limit` is deprecated, use `iteration_range` instead.
## [10:25:13] WARNING: amalgamation/../src/c_api/c_api.cc:718: `ntree_limit` is deprecated, use `iteration_range` instead.
## [10:25:13] WARNING: amalgamation/../src/c_api/c_api.cc:718: `ntree_limit` is deprecated, use `iteration_range` instead.
## [10:25:13] WARNING: amalgamation/../src/c_api/c_api.cc:718: `ntree_limit` is deprecated, use `iteration_range` instead.
## [10:25:13] WARNING: amalgamation/../src/c_api/c_api.cc:718: `ntree_limit` is deprecated, use `iteration_range` instead.
## [10:25:13] WARNING: amalgamation/../src/c_api/c_api.cc:718: `ntree_limit` is deprecated, use `iteration_range` instead.
## [10:25:13] WARNING: amalgamation/../src/c_api/c_api.cc:718: `ntree_limit` is deprecated, use `iteration_range` instead.
## [10:25:13] WARNING: amalgamation/../src/c_api/c_api.cc:718: `ntree_limit` is deprecated, use `iteration_range` instead.
## [10:25:13] WARNING: amalgamation/../src/c_api/c_api.cc:718: `ntree_limit` is deprecated, use `iteration_range` instead.
## [10:25:13] WARNING: amalgamation/../src/c_api/c_api.cc:718: `ntree_limit` is deprecated, use `iteration_range` instead.
## [10:25:13] WARNING: amalgamation/../src/c_api/c_api.cc:718: `ntree_limit` is deprecated, use `iteration_range` instead.
## [10:25:13] WARNING: amalgamation/../src/c_api/c_api.cc:718: `ntree_limit` is deprecated, use `iteration_range` instead.
## [10:25:13] WARNING: amalgamation/../src/c_api/c_api.cc:718: `ntree_limit` is deprecated, use `iteration_range` instead.
## [10:25:13] WARNING: amalgamation/../src/c_api/c_api.cc:718: `ntree_limit` is deprecated, use `iteration_range` instead.
## [10:25:13] WARNING: amalgamation/../src/c_api/c_api.cc:718: `ntree_limit` is deprecated, use `iteration_range` instead.
## [10:25:13] WARNING: amalgamation/../src/c_api/c_api.cc:718: `ntree_limit` is deprecated, use `iteration_range` instead.
## [10:25:13] WARNING: amalgamation/../src/c_api/c_api.cc:718: `ntree_limit` is deprecated, use `iteration_range` instead.
## [10:25:13] WARNING: amalgamation/../src/c_api/c_api.cc:718: `ntree_limit` is deprecated, use `iteration_range` instead.
## [10:25:13] WARNING: amalgamation/../src/c_api/c_api.cc:718: `ntree_limit` is deprecated, use `iteration_range` instead.
## [10:25:13] WARNING: amalgamation/../src/c_api/c_api.cc:718: `ntree_limit` is deprecated, use `iteration_range` instead.
## [10:25:13] WARNING: amalgamation/../src/c_api/c_api.cc:718: `ntree_limit` is deprecated, use `iteration_range` instead.
## [10:25:13] WARNING: amalgamation/../src/c_api/c_api.cc:718: `ntree_limit` is deprecated, use `iteration_range` instead.
## [10:25:13] WARNING: amalgamation/../src/c_api/c_api.cc:718: `ntree_limit` is deprecated, use `iteration_range` instead.
## [10:25:13] WARNING: amalgamation/../src/c_api/c_api.cc:718: `ntree_limit` is deprecated, use `iteration_range` instead.
## [10:25:13] WARNING: amalgamation/../src/c_api/c_api.cc:718: `ntree_limit` is deprecated, use `iteration_range` instead.
## [10:25:13] WARNING: amalgamation/../src/c_api/c_api.cc:718: `ntree_limit` is deprecated, use `iteration_range` instead.
## [10:25:13] WARNING: amalgamation/../src/c_api/c_api.cc:718: `ntree_limit` is deprecated, use `iteration_range` instead.
## [10:25:13] WARNING: amalgamation/../src/c_api/c_api.cc:718: `ntree_limit` is deprecated, use `iteration_range` instead.
## [10:25:13] WARNING: amalgamation/../src/c_api/c_api.cc:718: `ntree_limit` is deprecated, use `iteration_range` instead.
## [10:25:13] WARNING: amalgamation/../src/c_api/c_api.cc:718: `ntree_limit` is deprecated, use `iteration_range` instead.
## [10:25:13] WARNING: amalgamation/../src/c_api/c_api.cc:718: `ntree_limit` is deprecated, use `iteration_range` instead.
## [10:25:13] WARNING: amalgamation/../src/c_api/c_api.cc:718: `ntree_limit` is deprecated, use `iteration_range` instead.
## [10:25:13] WARNING: amalgamation/../src/c_api/c_api.cc:718: `ntree_limit` is deprecated, use `iteration_range` instead.
## [10:25:13] WARNING: amalgamation/../src/c_api/c_api.cc:718: `ntree_limit` is deprecated, use `iteration_range` instead.
## [10:25:13] WARNING: amalgamation/../src/c_api/c_api.cc:718: `ntree_limit` is deprecated, use `iteration_range` instead.
## [10:25:13] WARNING: amalgamation/../src/c_api/c_api.cc:718: `ntree_limit` is deprecated, use `iteration_range` instead.
## [10:25:13] WARNING: amalgamation/../src/c_api/c_api.cc:718: `ntree_limit` is deprecated, use `iteration_range` instead.
## [10:25:13] WARNING: amalgamation/../src/c_api/c_api.cc:718: `ntree_limit` is deprecated, use `iteration_range` instead.
## [10:25:13] WARNING: amalgamation/../src/c_api/c_api.cc:718: `ntree_limit` is deprecated, use `iteration_range` instead.
## [10:25:13] WARNING: amalgamation/../src/c_api/c_api.cc:718: `ntree_limit` is deprecated, use `iteration_range` instead.
## [10:25:13] WARNING: amalgamation/../src/c_api/c_api.cc:718: `ntree_limit` is deprecated, use `iteration_range` instead.
## [10:25:13] WARNING: amalgamation/../src/c_api/c_api.cc:718: `ntree_limit` is deprecated, use `iteration_range` instead.
## [10:25:13] WARNING: amalgamation/../src/c_api/c_api.cc:718: `ntree_limit` is deprecated, use `iteration_range` instead.
## [10:25:13] WARNING: amalgamation/../src/c_api/c_api.cc:718: `ntree_limit` is deprecated, use `iteration_range` instead.
## [10:25:13] WARNING: amalgamation/../src/c_api/c_api.cc:718: `ntree_limit` is deprecated, use `iteration_range` instead.
## [10:25:13] WARNING: amalgamation/../src/c_api/c_api.cc:718: `ntree_limit` is deprecated, use `iteration_range` instead.
## [10:25:13] WARNING: amalgamation/../src/c_api/c_api.cc:718: `ntree_limit` is deprecated, use `iteration_range` instead.
## [10:25:13] WARNING: amalgamation/../src/c_api/c_api.cc:718: `ntree_limit` is deprecated, use `iteration_range` instead.
## [10:25:13] WARNING: amalgamation/../src/c_api/c_api.cc:718: `ntree_limit` is deprecated, use `iteration_range` instead.
## [10:25:13] WARNING: amalgamation/../src/c_api/c_api.cc:718: `ntree_limit` is deprecated, use `iteration_range` instead.
## [10:25:13] WARNING: amalgamation/../src/c_api/c_api.cc:718: `ntree_limit` is deprecated, use `iteration_range` instead.
## [10:25:13] WARNING: amalgamation/../src/c_api/c_api.cc:718: `ntree_limit` is deprecated, use `iteration_range` instead.
## [10:25:13] WARNING: amalgamation/../src/c_api/c_api.cc:718: `ntree_limit` is deprecated, use `iteration_range` instead.
## [10:25:13] WARNING: amalgamation/../src/c_api/c_api.cc:718: `ntree_limit` is deprecated, use `iteration_range` instead.
## [10:25:13] WARNING: amalgamation/../src/c_api/c_api.cc:718: `ntree_limit` is deprecated, use `iteration_range` instead.
## [10:25:13] WARNING: amalgamation/../src/c_api/c_api.cc:718: `ntree_limit` is deprecated, use `iteration_range` instead.
## [10:25:13] WARNING: amalgamation/../src/c_api/c_api.cc:718: `ntree_limit` is deprecated, use `iteration_range` instead.
## [10:25:13] WARNING: amalgamation/../src/c_api/c_api.cc:718: `ntree_limit` is deprecated, use `iteration_range` instead.
## [10:25:13] WARNING: amalgamation/../src/c_api/c_api.cc:718: `ntree_limit` is deprecated, use `iteration_range` instead.
## [10:25:13] WARNING: amalgamation/../src/c_api/c_api.cc:718: `ntree_limit` is deprecated, use `iteration_range` instead.
## [10:25:13] WARNING: amalgamation/../src/c_api/c_api.cc:718: `ntree_limit` is deprecated, use `iteration_range` instead.
## [10:25:13] WARNING: amalgamation/../src/c_api/c_api.cc:718: `ntree_limit` is deprecated, use `iteration_range` instead.
## [10:25:13] WARNING: amalgamation/../src/c_api/c_api.cc:718: `ntree_limit` is deprecated, use `iteration_range` instead.
## [10:25:13] WARNING: amalgamation/../src/c_api/c_api.cc:718: `ntree_limit` is deprecated, use `iteration_range` instead.
## [10:25:13] WARNING: amalgamation/../src/c_api/c_api.cc:718: `ntree_limit` is deprecated, use `iteration_range` instead.
## [10:25:13] WARNING: amalgamation/../src/c_api/c_api.cc:718: `ntree_limit` is deprecated, use `iteration_range` instead.
## [10:25:13] WARNING: amalgamation/../src/c_api/c_api.cc:718: `ntree_limit` is deprecated, use `iteration_range` instead.
## [10:25:13] WARNING: amalgamation/../src/c_api/c_api.cc:718: `ntree_limit` is deprecated, use `iteration_range` instead.
## [10:25:13] WARNING: amalgamation/../src/c_api/c_api.cc:718: `ntree_limit` is deprecated, use `iteration_range` instead.
## [10:25:13] WARNING: amalgamation/../src/c_api/c_api.cc:718: `ntree_limit` is deprecated, use `iteration_range` instead.
## [10:25:13] WARNING: amalgamation/../src/c_api/c_api.cc:718: `ntree_limit` is deprecated, use `iteration_range` instead.
## [10:25:13] WARNING: amalgamation/../src/c_api/c_api.cc:718: `ntree_limit` is deprecated, use `iteration_range` instead.
## [10:25:13] WARNING: amalgamation/../src/c_api/c_api.cc:718: `ntree_limit` is deprecated, use `iteration_range` instead.
## [10:25:13] WARNING: amalgamation/../src/c_api/c_api.cc:718: `ntree_limit` is deprecated, use `iteration_range` instead.
## [10:25:13] WARNING: amalgamation/../src/c_api/c_api.cc:718: `ntree_limit` is deprecated, use `iteration_range` instead.
## [10:25:13] WARNING: amalgamation/../src/c_api/c_api.cc:718: `ntree_limit` is deprecated, use `iteration_range` instead.
## [10:25:13] WARNING: amalgamation/../src/c_api/c_api.cc:718: `ntree_limit` is deprecated, use `iteration_range` instead.
## [10:25:13] WARNING: amalgamation/../src/c_api/c_api.cc:718: `ntree_limit` is deprecated, use `iteration_range` instead.
## [10:25:13] WARNING: amalgamation/../src/c_api/c_api.cc:718: `ntree_limit` is deprecated, use `iteration_range` instead.
## [10:25:13] WARNING: amalgamation/../src/c_api/c_api.cc:718: `ntree_limit` is deprecated, use `iteration_range` instead.
## [10:25:13] WARNING: amalgamation/../src/c_api/c_api.cc:718: `ntree_limit` is deprecated, use `iteration_range` instead.
## [10:25:13] WARNING: amalgamation/../src/c_api/c_api.cc:718: `ntree_limit` is deprecated, use `iteration_range` instead.
## [10:25:13] WARNING: amalgamation/../src/c_api/c_api.cc:718: `ntree_limit` is deprecated, use `iteration_range` instead.
## [10:25:14] WARNING: amalgamation/../src/c_api/c_api.cc:718: `ntree_limit` is deprecated, use `iteration_range` instead.
## [10:25:14] WARNING: amalgamation/../src/c_api/c_api.cc:718: `ntree_limit` is deprecated, use `iteration_range` instead.
## [10:25:14] WARNING: amalgamation/../src/c_api/c_api.cc:718: `ntree_limit` is deprecated, use `iteration_range` instead.
## [10:25:14] WARNING: amalgamation/../src/c_api/c_api.cc:718: `ntree_limit` is deprecated, use `iteration_range` instead.
## [10:25:14] WARNING: amalgamation/../src/c_api/c_api.cc:718: `ntree_limit` is deprecated, use `iteration_range` instead.
## [10:25:14] WARNING: amalgamation/../src/c_api/c_api.cc:718: `ntree_limit` is deprecated, use `iteration_range` instead.
## [10:25:14] WARNING: amalgamation/../src/c_api/c_api.cc:718: `ntree_limit` is deprecated, use `iteration_range` instead.
## [10:25:14] WARNING: amalgamation/../src/c_api/c_api.cc:718: `ntree_limit` is deprecated, use `iteration_range` instead.
## [10:25:14] WARNING: amalgamation/../src/c_api/c_api.cc:718: `ntree_limit` is deprecated, use `iteration_range` instead.
## [10:25:14] WARNING: amalgamation/../src/c_api/c_api.cc:718: `ntree_limit` is deprecated, use `iteration_range` instead.
## [10:25:14] WARNING: amalgamation/../src/c_api/c_api.cc:718: `ntree_limit` is deprecated, use `iteration_range` instead.
## [10:25:14] WARNING: amalgamation/../src/c_api/c_api.cc:718: `ntree_limit` is deprecated, use `iteration_range` instead.
## [10:25:14] WARNING: amalgamation/../src/c_api/c_api.cc:718: `ntree_limit` is deprecated, use `iteration_range` instead.
## [10:25:14] WARNING: amalgamation/../src/c_api/c_api.cc:718: `ntree_limit` is deprecated, use `iteration_range` instead.
## [10:25:14] WARNING: amalgamation/../src/c_api/c_api.cc:718: `ntree_limit` is deprecated, use `iteration_range` instead.
## [10:25:14] WARNING: amalgamation/../src/c_api/c_api.cc:718: `ntree_limit` is deprecated, use `iteration_range` instead.
## [10:25:14] WARNING: amalgamation/../src/c_api/c_api.cc:718: `ntree_limit` is deprecated, use `iteration_range` instead.
## [10:25:14] WARNING: amalgamation/../src/c_api/c_api.cc:718: `ntree_limit` is deprecated, use `iteration_range` instead.
## [10:25:14] WARNING: amalgamation/../src/c_api/c_api.cc:718: `ntree_limit` is deprecated, use `iteration_range` instead.
## [10:25:14] WARNING: amalgamation/../src/c_api/c_api.cc:718: `ntree_limit` is deprecated, use `iteration_range` instead.
## [10:25:14] WARNING: amalgamation/../src/c_api/c_api.cc:718: `ntree_limit` is deprecated, use `iteration_range` instead.
## [10:25:14] WARNING: amalgamation/../src/c_api/c_api.cc:718: `ntree_limit` is deprecated, use `iteration_range` instead.
## [10:25:14] WARNING: amalgamation/../src/c_api/c_api.cc:718: `ntree_limit` is deprecated, use `iteration_range` instead.
## [10:25:14] WARNING: amalgamation/../src/c_api/c_api.cc:718: `ntree_limit` is deprecated, use `iteration_range` instead.
## [10:25:14] WARNING: amalgamation/../src/c_api/c_api.cc:718: `ntree_limit` is deprecated, use `iteration_range` instead.
## [10:25:14] WARNING: amalgamation/../src/c_api/c_api.cc:718: `ntree_limit` is deprecated, use `iteration_range` instead.
## [10:25:14] WARNING: amalgamation/../src/c_api/c_api.cc:718: `ntree_limit` is deprecated, use `iteration_range` instead.
## [10:25:14] WARNING: amalgamation/../src/c_api/c_api.cc:718: `ntree_limit` is deprecated, use `iteration_range` instead.
## [10:25:14] WARNING: amalgamation/../src/c_api/c_api.cc:718: `ntree_limit` is deprecated, use `iteration_range` instead.
## [10:25:14] WARNING: amalgamation/../src/c_api/c_api.cc:718: `ntree_limit` is deprecated, use `iteration_range` instead.
## [10:25:14] WARNING: amalgamation/../src/c_api/c_api.cc:718: `ntree_limit` is deprecated, use `iteration_range` instead.
## [10:25:14] WARNING: amalgamation/../src/c_api/c_api.cc:718: `ntree_limit` is deprecated, use `iteration_range` instead.
## [10:25:14] WARNING: amalgamation/../src/c_api/c_api.cc:718: `ntree_limit` is deprecated, use `iteration_range` instead.
## [10:25:14] WARNING: amalgamation/../src/c_api/c_api.cc:718: `ntree_limit` is deprecated, use `iteration_range` instead.
## [10:25:14] WARNING: amalgamation/../src/c_api/c_api.cc:718: `ntree_limit` is deprecated, use `iteration_range` instead.
## [10:25:14] WARNING: amalgamation/../src/c_api/c_api.cc:718: `ntree_limit` is deprecated, use `iteration_range` instead.
## [10:25:14] WARNING: amalgamation/../src/c_api/c_api.cc:718: `ntree_limit` is deprecated, use `iteration_range` instead.
## [10:25:14] WARNING: amalgamation/../src/c_api/c_api.cc:718: `ntree_limit` is deprecated, use `iteration_range` instead.
## [10:25:14] WARNING: amalgamation/../src/c_api/c_api.cc:718: `ntree_limit` is deprecated, use `iteration_range` instead.
## [10:25:14] WARNING: amalgamation/../src/c_api/c_api.cc:718: `ntree_limit` is deprecated, use `iteration_range` instead.
## [10:25:14] WARNING: amalgamation/../src/c_api/c_api.cc:718: `ntree_limit` is deprecated, use `iteration_range` instead.
## [10:25:14] WARNING: amalgamation/../src/c_api/c_api.cc:718: `ntree_limit` is deprecated, use `iteration_range` instead.
## [10:25:14] WARNING: amalgamation/../src/c_api/c_api.cc:718: `ntree_limit` is deprecated, use `iteration_range` instead.
## [10:25:14] WARNING: amalgamation/../src/c_api/c_api.cc:718: `ntree_limit` is deprecated, use `iteration_range` instead.
## [10:25:14] WARNING: amalgamation/../src/c_api/c_api.cc:718: `ntree_limit` is deprecated, use `iteration_range` instead.
## [10:25:14] WARNING: amalgamation/../src/c_api/c_api.cc:718: `ntree_limit` is deprecated, use `iteration_range` instead.
## [10:25:14] WARNING: amalgamation/../src/c_api/c_api.cc:718: `ntree_limit` is deprecated, use `iteration_range` instead.
## [10:25:14] WARNING: amalgamation/../src/c_api/c_api.cc:718: `ntree_limit` is deprecated, use `iteration_range` instead.
## [10:25:14] WARNING: amalgamation/../src/c_api/c_api.cc:718: `ntree_limit` is deprecated, use `iteration_range` instead.
## [10:25:14] WARNING: amalgamation/../src/c_api/c_api.cc:718: `ntree_limit` is deprecated, use `iteration_range` instead.
## [10:25:14] WARNING: amalgamation/../src/c_api/c_api.cc:718: `ntree_limit` is deprecated, use `iteration_range` instead.
## [10:25:14] WARNING: amalgamation/../src/c_api/c_api.cc:718: `ntree_limit` is deprecated, use `iteration_range` instead.
## [10:25:14] WARNING: amalgamation/../src/c_api/c_api.cc:718: `ntree_limit` is deprecated, use `iteration_range` instead.
## [10:25:14] WARNING: amalgamation/../src/c_api/c_api.cc:718: `ntree_limit` is deprecated, use `iteration_range` instead.
## [10:25:14] WARNING: amalgamation/../src/c_api/c_api.cc:718: `ntree_limit` is deprecated, use `iteration_range` instead.
## [10:25:14] WARNING: amalgamation/../src/c_api/c_api.cc:718: `ntree_limit` is deprecated, use `iteration_range` instead.
## [10:25:14] WARNING: amalgamation/../src/c_api/c_api.cc:718: `ntree_limit` is deprecated, use `iteration_range` instead.
## [10:25:14] WARNING: amalgamation/../src/c_api/c_api.cc:718: `ntree_limit` is deprecated, use `iteration_range` instead.
## [10:25:14] WARNING: amalgamation/../src/c_api/c_api.cc:718: `ntree_limit` is deprecated, use `iteration_range` instead.
## [10:25:14] WARNING: amalgamation/../src/c_api/c_api.cc:718: `ntree_limit` is deprecated, use `iteration_range` instead.
## [10:25:14] WARNING: amalgamation/../src/c_api/c_api.cc:718: `ntree_limit` is deprecated, use `iteration_range` instead.
## [10:25:14] WARNING: amalgamation/../src/c_api/c_api.cc:718: `ntree_limit` is deprecated, use `iteration_range` instead.
## [10:25:14] WARNING: amalgamation/../src/c_api/c_api.cc:718: `ntree_limit` is deprecated, use `iteration_range` instead.
## [10:25:14] WARNING: amalgamation/../src/c_api/c_api.cc:718: `ntree_limit` is deprecated, use `iteration_range` instead.
## [10:25:14] WARNING: amalgamation/../src/c_api/c_api.cc:718: `ntree_limit` is deprecated, use `iteration_range` instead.
## [10:25:14] WARNING: amalgamation/../src/c_api/c_api.cc:718: `ntree_limit` is deprecated, use `iteration_range` instead.
## [10:25:14] WARNING: amalgamation/../src/c_api/c_api.cc:718: `ntree_limit` is deprecated, use `iteration_range` instead.
## [10:25:14] WARNING: amalgamation/../src/c_api/c_api.cc:718: `ntree_limit` is deprecated, use `iteration_range` instead.
## [10:25:14] WARNING: amalgamation/../src/c_api/c_api.cc:718: `ntree_limit` is deprecated, use `iteration_range` instead.
## [10:25:14] WARNING: amalgamation/../src/c_api/c_api.cc:718: `ntree_limit` is deprecated, use `iteration_range` instead.
## [10:25:14] WARNING: amalgamation/../src/c_api/c_api.cc:718: `ntree_limit` is deprecated, use `iteration_range` instead.
## [10:25:14] WARNING: amalgamation/../src/c_api/c_api.cc:718: `ntree_limit` is deprecated, use `iteration_range` instead.
## [10:25:14] WARNING: amalgamation/../src/c_api/c_api.cc:718: `ntree_limit` is deprecated, use `iteration_range` instead.
## [10:25:14] WARNING: amalgamation/../src/c_api/c_api.cc:718: `ntree_limit` is deprecated, use `iteration_range` instead.
## [10:25:14] WARNING: amalgamation/../src/c_api/c_api.cc:718: `ntree_limit` is deprecated, use `iteration_range` instead.
## [10:25:14] WARNING: amalgamation/../src/c_api/c_api.cc:718: `ntree_limit` is deprecated, use `iteration_range` instead.
## [10:25:14] WARNING: amalgamation/../src/c_api/c_api.cc:718: `ntree_limit` is deprecated, use `iteration_range` instead.
## [10:25:14] WARNING: amalgamation/../src/c_api/c_api.cc:718: `ntree_limit` is deprecated, use `iteration_range` instead.
## [10:25:14] WARNING: amalgamation/../src/c_api/c_api.cc:718: `ntree_limit` is deprecated, use `iteration_range` instead.
## [10:25:14] WARNING: amalgamation/../src/c_api/c_api.cc:718: `ntree_limit` is deprecated, use `iteration_range` instead.
## [10:25:14] WARNING: amalgamation/../src/c_api/c_api.cc:718: `ntree_limit` is deprecated, use `iteration_range` instead.
## [10:25:14] WARNING: amalgamation/../src/c_api/c_api.cc:718: `ntree_limit` is deprecated, use `iteration_range` instead.
## [10:25:14] WARNING: amalgamation/../src/c_api/c_api.cc:718: `ntree_limit` is deprecated, use `iteration_range` instead.
## [10:25:14] WARNING: amalgamation/../src/c_api/c_api.cc:718: `ntree_limit` is deprecated, use `iteration_range` instead.
## [10:25:14] WARNING: amalgamation/../src/c_api/c_api.cc:718: `ntree_limit` is deprecated, use `iteration_range` instead.
## [10:25:14] WARNING: amalgamation/../src/c_api/c_api.cc:718: `ntree_limit` is deprecated, use `iteration_range` instead.
## [10:25:14] WARNING: amalgamation/../src/c_api/c_api.cc:718: `ntree_limit` is deprecated, use `iteration_range` instead.
## [10:25:14] WARNING: amalgamation/../src/c_api/c_api.cc:718: `ntree_limit` is deprecated, use `iteration_range` instead.
## [10:25:14] WARNING: amalgamation/../src/c_api/c_api.cc:718: `ntree_limit` is deprecated, use `iteration_range` instead.
## [10:25:14] WARNING: amalgamation/../src/c_api/c_api.cc:718: `ntree_limit` is deprecated, use `iteration_range` instead.
## [10:25:14] WARNING: amalgamation/../src/c_api/c_api.cc:718: `ntree_limit` is deprecated, use `iteration_range` instead.
## [10:25:14] WARNING: amalgamation/../src/c_api/c_api.cc:718: `ntree_limit` is deprecated, use `iteration_range` instead.
## [10:25:14] WARNING: amalgamation/../src/c_api/c_api.cc:718: `ntree_limit` is deprecated, use `iteration_range` instead.
## [10:25:14] WARNING: amalgamation/../src/c_api/c_api.cc:718: `ntree_limit` is deprecated, use `iteration_range` instead.
## [10:25:14] WARNING: amalgamation/../src/c_api/c_api.cc:718: `ntree_limit` is deprecated, use `iteration_range` instead.
## [10:25:14] WARNING: amalgamation/../src/c_api/c_api.cc:718: `ntree_limit` is deprecated, use `iteration_range` instead.
## [10:25:14] WARNING: amalgamation/../src/c_api/c_api.cc:718: `ntree_limit` is deprecated, use `iteration_range` instead.
## [10:25:14] WARNING: amalgamation/../src/c_api/c_api.cc:718: `ntree_limit` is deprecated, use `iteration_range` instead.
## [10:25:14] WARNING: amalgamation/../src/c_api/c_api.cc:718: `ntree_limit` is deprecated, use `iteration_range` instead.
## [10:25:14] WARNING: amalgamation/../src/c_api/c_api.cc:718: `ntree_limit` is deprecated, use `iteration_range` instead.
## [10:25:14] WARNING: amalgamation/../src/c_api/c_api.cc:718: `ntree_limit` is deprecated, use `iteration_range` instead.
## [10:25:14] WARNING: amalgamation/../src/c_api/c_api.cc:718: `ntree_limit` is deprecated, use `iteration_range` instead.
## [10:25:14] WARNING: amalgamation/../src/c_api/c_api.cc:718: `ntree_limit` is deprecated, use `iteration_range` instead.
## [10:25:14] WARNING: amalgamation/../src/c_api/c_api.cc:718: `ntree_limit` is deprecated, use `iteration_range` instead.
## [10:25:14] WARNING: amalgamation/../src/c_api/c_api.cc:718: `ntree_limit` is deprecated, use `iteration_range` instead.
## [10:25:14] WARNING: amalgamation/../src/c_api/c_api.cc:718: `ntree_limit` is deprecated, use `iteration_range` instead.
## [10:25:14] WARNING: amalgamation/../src/c_api/c_api.cc:718: `ntree_limit` is deprecated, use `iteration_range` instead.
## [10:25:14] WARNING: amalgamation/../src/c_api/c_api.cc:718: `ntree_limit` is deprecated, use `iteration_range` instead.
## [10:25:14] WARNING: amalgamation/../src/c_api/c_api.cc:718: `ntree_limit` is deprecated, use `iteration_range` instead.
## [10:25:14] WARNING: amalgamation/../src/c_api/c_api.cc:718: `ntree_limit` is deprecated, use `iteration_range` instead.
## [10:25:14] WARNING: amalgamation/../src/c_api/c_api.cc:718: `ntree_limit` is deprecated, use `iteration_range` instead.
## [10:25:14] WARNING: amalgamation/../src/c_api/c_api.cc:718: `ntree_limit` is deprecated, use `iteration_range` instead.
## [10:25:14] WARNING: amalgamation/../src/c_api/c_api.cc:718: `ntree_limit` is deprecated, use `iteration_range` instead.
## [10:25:14] WARNING: amalgamation/../src/c_api/c_api.cc:718: `ntree_limit` is deprecated, use `iteration_range` instead.
## [10:25:14] WARNING: amalgamation/../src/c_api/c_api.cc:718: `ntree_limit` is deprecated, use `iteration_range` instead.
## [10:25:14] WARNING: amalgamation/../src/c_api/c_api.cc:718: `ntree_limit` is deprecated, use `iteration_range` instead.
## [10:25:14] WARNING: amalgamation/../src/c_api/c_api.cc:718: `ntree_limit` is deprecated, use `iteration_range` instead.
## [10:25:14] WARNING: amalgamation/../src/c_api/c_api.cc:718: `ntree_limit` is deprecated, use `iteration_range` instead.
## [10:25:14] WARNING: amalgamation/../src/c_api/c_api.cc:718: `ntree_limit` is deprecated, use `iteration_range` instead.
## [10:25:14] WARNING: amalgamation/../src/c_api/c_api.cc:718: `ntree_limit` is deprecated, use `iteration_range` instead.
## [10:25:14] WARNING: amalgamation/../src/c_api/c_api.cc:718: `ntree_limit` is deprecated, use `iteration_range` instead.
## [10:25:14] WARNING: amalgamation/../src/c_api/c_api.cc:718: `ntree_limit` is deprecated, use `iteration_range` instead.
## [10:25:14] WARNING: amalgamation/../src/c_api/c_api.cc:718: `ntree_limit` is deprecated, use `iteration_range` instead.
## [10:25:14] WARNING: amalgamation/../src/c_api/c_api.cc:718: `ntree_limit` is deprecated, use `iteration_range` instead.
## [10:25:14] WARNING: amalgamation/../src/c_api/c_api.cc:718: `ntree_limit` is deprecated, use `iteration_range` instead.
## [10:25:14] WARNING: amalgamation/../src/c_api/c_api.cc:718: `ntree_limit` is deprecated, use `iteration_range` instead.
## [10:25:14] WARNING: amalgamation/../src/c_api/c_api.cc:718: `ntree_limit` is deprecated, use `iteration_range` instead.
## [10:25:14] WARNING: amalgamation/../src/c_api/c_api.cc:718: `ntree_limit` is deprecated, use `iteration_range` instead.
## [10:25:14] WARNING: amalgamation/../src/c_api/c_api.cc:718: `ntree_limit` is deprecated, use `iteration_range` instead.
## [10:25:14] WARNING: amalgamation/../src/c_api/c_api.cc:718: `ntree_limit` is deprecated, use `iteration_range` instead.
## [10:25:14] WARNING: amalgamation/../src/c_api/c_api.cc:718: `ntree_limit` is deprecated, use `iteration_range` instead.
## [10:25:14] WARNING: amalgamation/../src/c_api/c_api.cc:718: `ntree_limit` is deprecated, use `iteration_range` instead.
## [10:25:14] WARNING: amalgamation/../src/c_api/c_api.cc:718: `ntree_limit` is deprecated, use `iteration_range` instead.
## [10:25:14] WARNING: amalgamation/../src/c_api/c_api.cc:718: `ntree_limit` is deprecated, use `iteration_range` instead.
## [10:25:14] WARNING: amalgamation/../src/c_api/c_api.cc:718: `ntree_limit` is deprecated, use `iteration_range` instead.
## [10:25:14] WARNING: amalgamation/../src/c_api/c_api.cc:718: `ntree_limit` is deprecated, use `iteration_range` instead.
## [10:25:14] WARNING: amalgamation/../src/c_api/c_api.cc:718: `ntree_limit` is deprecated, use `iteration_range` instead.
## [10:25:14] WARNING: amalgamation/../src/c_api/c_api.cc:718: `ntree_limit` is deprecated, use `iteration_range` instead.
## [10:25:14] WARNING: amalgamation/../src/c_api/c_api.cc:718: `ntree_limit` is deprecated, use `iteration_range` instead.
## [10:25:14] WARNING: amalgamation/../src/c_api/c_api.cc:718: `ntree_limit` is deprecated, use `iteration_range` instead.
## [10:25:14] WARNING: amalgamation/../src/c_api/c_api.cc:718: `ntree_limit` is deprecated, use `iteration_range` instead.
## [10:25:14] WARNING: amalgamation/../src/c_api/c_api.cc:718: `ntree_limit` is deprecated, use `iteration_range` instead.
## [10:25:14] WARNING: amalgamation/../src/c_api/c_api.cc:718: `ntree_limit` is deprecated, use `iteration_range` instead.
## [10:25:14] WARNING: amalgamation/../src/c_api/c_api.cc:718: `ntree_limit` is deprecated, use `iteration_range` instead.
## [10:25:14] WARNING: amalgamation/../src/c_api/c_api.cc:718: `ntree_limit` is deprecated, use `iteration_range` instead.
## [10:25:14] WARNING: amalgamation/../src/c_api/c_api.cc:718: `ntree_limit` is deprecated, use `iteration_range` instead.
## [10:25:14] WARNING: amalgamation/../src/c_api/c_api.cc:718: `ntree_limit` is deprecated, use `iteration_range` instead.
## [10:25:14] WARNING: amalgamation/../src/c_api/c_api.cc:718: `ntree_limit` is deprecated, use `iteration_range` instead.
## [10:25:14] WARNING: amalgamation/../src/c_api/c_api.cc:718: `ntree_limit` is deprecated, use `iteration_range` instead.
## [10:25:14] WARNING: amalgamation/../src/c_api/c_api.cc:718: `ntree_limit` is deprecated, use `iteration_range` instead.
## [10:25:14] WARNING: amalgamation/../src/c_api/c_api.cc:718: `ntree_limit` is deprecated, use `iteration_range` instead.
## [10:25:14] WARNING: amalgamation/../src/c_api/c_api.cc:718: `ntree_limit` is deprecated, use `iteration_range` instead.
## [10:25:14] WARNING: amalgamation/../src/c_api/c_api.cc:718: `ntree_limit` is deprecated, use `iteration_range` instead.
## [10:25:14] WARNING: amalgamation/../src/c_api/c_api.cc:718: `ntree_limit` is deprecated, use `iteration_range` instead.
## [10:25:14] WARNING: amalgamation/../src/c_api/c_api.cc:718: `ntree_limit` is deprecated, use `iteration_range` instead.
## [10:25:14] WARNING: amalgamation/../src/c_api/c_api.cc:718: `ntree_limit` is deprecated, use `iteration_range` instead.
## [10:25:14] WARNING: amalgamation/../src/c_api/c_api.cc:718: `ntree_limit` is deprecated, use `iteration_range` instead.
## [10:25:14] WARNING: amalgamation/../src/c_api/c_api.cc:718: `ntree_limit` is deprecated, use `iteration_range` instead.
## [10:25:14] WARNING: amalgamation/../src/c_api/c_api.cc:718: `ntree_limit` is deprecated, use `iteration_range` instead.
## [10:25:14] WARNING: amalgamation/../src/c_api/c_api.cc:718: `ntree_limit` is deprecated, use `iteration_range` instead.
## [10:25:14] WARNING: amalgamation/../src/c_api/c_api.cc:718: `ntree_limit` is deprecated, use `iteration_range` instead.
## [10:25:14] WARNING: amalgamation/../src/c_api/c_api.cc:718: `ntree_limit` is deprecated, use `iteration_range` instead.
## [10:25:15] WARNING: amalgamation/../src/c_api/c_api.cc:718: `ntree_limit` is deprecated, use `iteration_range` instead.
## [10:25:15] WARNING: amalgamation/../src/c_api/c_api.cc:718: `ntree_limit` is deprecated, use `iteration_range` instead.
## [10:25:15] WARNING: amalgamation/../src/c_api/c_api.cc:718: `ntree_limit` is deprecated, use `iteration_range` instead.
## [10:25:15] WARNING: amalgamation/../src/c_api/c_api.cc:718: `ntree_limit` is deprecated, use `iteration_range` instead.
## [10:25:15] WARNING: amalgamation/../src/c_api/c_api.cc:718: `ntree_limit` is deprecated, use `iteration_range` instead.
## [10:25:15] WARNING: amalgamation/../src/c_api/c_api.cc:718: `ntree_limit` is deprecated, use `iteration_range` instead.
## [10:25:15] WARNING: amalgamation/../src/c_api/c_api.cc:718: `ntree_limit` is deprecated, use `iteration_range` instead.
## [10:25:15] WARNING: amalgamation/../src/c_api/c_api.cc:718: `ntree_limit` is deprecated, use `iteration_range` instead.
## [10:25:15] WARNING: amalgamation/../src/c_api/c_api.cc:718: `ntree_limit` is deprecated, use `iteration_range` instead.
## [10:25:15] WARNING: amalgamation/../src/c_api/c_api.cc:718: `ntree_limit` is deprecated, use `iteration_range` instead.
## [10:25:15] WARNING: amalgamation/../src/c_api/c_api.cc:718: `ntree_limit` is deprecated, use `iteration_range` instead.
## [10:25:15] WARNING: amalgamation/../src/c_api/c_api.cc:718: `ntree_limit` is deprecated, use `iteration_range` instead.
## [10:25:15] WARNING: amalgamation/../src/c_api/c_api.cc:718: `ntree_limit` is deprecated, use `iteration_range` instead.
## [10:25:15] WARNING: amalgamation/../src/c_api/c_api.cc:718: `ntree_limit` is deprecated, use `iteration_range` instead.
## [10:25:15] WARNING: amalgamation/../src/c_api/c_api.cc:718: `ntree_limit` is deprecated, use `iteration_range` instead.
## [10:25:15] WARNING: amalgamation/../src/c_api/c_api.cc:718: `ntree_limit` is deprecated, use `iteration_range` instead.
## [10:25:15] WARNING: amalgamation/../src/c_api/c_api.cc:718: `ntree_limit` is deprecated, use `iteration_range` instead.
## [10:25:15] WARNING: amalgamation/../src/c_api/c_api.cc:718: `ntree_limit` is deprecated, use `iteration_range` instead.
## [10:25:15] WARNING: amalgamation/../src/c_api/c_api.cc:718: `ntree_limit` is deprecated, use `iteration_range` instead.
## [10:25:15] WARNING: amalgamation/../src/c_api/c_api.cc:718: `ntree_limit` is deprecated, use `iteration_range` instead.
## [10:25:15] WARNING: amalgamation/../src/c_api/c_api.cc:718: `ntree_limit` is deprecated, use `iteration_range` instead.
## [10:25:15] WARNING: amalgamation/../src/c_api/c_api.cc:718: `ntree_limit` is deprecated, use `iteration_range` instead.
## [10:25:15] WARNING: amalgamation/../src/c_api/c_api.cc:718: `ntree_limit` is deprecated, use `iteration_range` instead.
## [10:25:15] WARNING: amalgamation/../src/c_api/c_api.cc:718: `ntree_limit` is deprecated, use `iteration_range` instead.
## [10:25:15] WARNING: amalgamation/../src/c_api/c_api.cc:718: `ntree_limit` is deprecated, use `iteration_range` instead.
## [10:25:15] WARNING: amalgamation/../src/c_api/c_api.cc:718: `ntree_limit` is deprecated, use `iteration_range` instead.
## [10:25:15] WARNING: amalgamation/../src/c_api/c_api.cc:718: `ntree_limit` is deprecated, use `iteration_range` instead.
## [10:25:15] WARNING: amalgamation/../src/c_api/c_api.cc:718: `ntree_limit` is deprecated, use `iteration_range` instead.
## [10:25:15] WARNING: amalgamation/../src/c_api/c_api.cc:718: `ntree_limit` is deprecated, use `iteration_range` instead.
## [10:25:15] WARNING: amalgamation/../src/c_api/c_api.cc:718: `ntree_limit` is deprecated, use `iteration_range` instead.
```

```
## eXtreme Gradient Boosting 
## 
## 104 samples
##  74 predictor
##   2 classes: 'FALSE', 'TRUE' 
## 
## No pre-processing
## Resampling: Cross-Validated (3 fold) 
## Summary of sample sizes: 70, 69, 69 
## Resampling results:
## 
##   Accuracy   Kappa     
##   0.6913165  0.09215387
## 
## Tuning parameter 'nrounds' was held constant at a value of 5
## Tuning
##  held constant at a value of 1
## Tuning parameter 'subsample' was held
##  constant at a value of 1
```

```
## $A
## [1] 0.8009868
## 
## $n.total
## [1] 104
## 
## $n.events
## [1] 28
## 
## $n.noevents
## [1] 76
## 
## $p.value
## [1] 2.89328e-07
```

```
## Warning in wilcox.test.default(pred[obs == 1], pred[obs == 0], alternative =
## "great"): cannot compute exact p-value with ties
```

```
## $A
## [1] 0.7606178
## 
## $n.total
## [1] 51
## 
## $n.events
## [1] 14
## 
## $n.noevents
## [1] 37
## 
## $p.value
## [1] 0.00133722
```

```
##   [1] "PREVIOUS" "PREVIOUS" "PREVIOUS" "PREVIOUS" "PREVIOUS" "PREVIOUS"
##   [7] "NEVER"    "NEVER"    "CURRENT"  "PREVIOUS" "NEVER"    "PREVIOUS"
##  [13] "PREVIOUS" "PREVIOUS" "NEVER"    "PREVIOUS" "PREVIOUS" "PREVIOUS"
##  [19] "PREVIOUS" "PREVIOUS" "PREVIOUS" "PREVIOUS" "PREVIOUS" "PREVIOUS"
##  [25] "PREVIOUS" "CURRENT"  "CURRENT"  "CURRENT"  "PREVIOUS" "PREVIOUS"
##  [31] "PREVIOUS" "NEVER"    "CURRENT"  "NEVER"    "PREVIOUS" "NEVER"   
##  [37] "PREVIOUS" "PREVIOUS" "NEVER"    "PREVIOUS" "NEVER"    "PREVIOUS"
##  [43] "PREVIOUS" "NEVER"    "PREVIOUS" "PREVIOUS" "NEVER"    "PREVIOUS"
##  [49] "NEVER"    "PREVIOUS" "PREVIOUS" "PREVIOUS" "PREVIOUS" "PREVIOUS"
##  [55] "NEVER"    "NEVER"    "NEVER"    "NEVER"    "PREVIOUS" "NEVER"   
##  [61] "NEVER"    "NEVER"    "NEVER"    "PREVIOUS" "CURRENT"  "PREVIOUS"
##  [67] "PREVIOUS" "PREVIOUS" "CURRENT"  "CURRENT"  "PREVIOUS" "NEVER"   
##  [73] "PREVIOUS" "PREVIOUS" "PREVIOUS" "NEVER"    "PREVIOUS" "NEVER"   
##  [79] "NEVER"    "PREVIOUS" "CURRENT"  "PREVIOUS" "PREVIOUS" "NEVER"   
##  [85] "NEVER"    "CURRENT"  "NEVER"    "PREVIOUS" "PREVIOUS" "NEVER"   
##  [91] "NEVER"    "NEVER"    "NEVER"    "NEVER"    "PREVIOUS" "NEVER"   
##  [97] "PREVIOUS" "CURRENT"  "NEVER"    "PREVIOUS" "PREVIOUS" "PREVIOUS"
## [103] "PREVIOUS" "PREVIOUS" "PREVIOUS" "PREVIOUS" "NEVER"    "PREVIOUS"
## [109] "PREVIOUS" "NEVER"    "PREVIOUS" "NEVER"    "PREVIOUS" "PREVIOUS"
## [115] "CURRENT"  "NEVER"    "PREVIOUS" "NEVER"    "PREVIOUS" "PREVIOUS"
## [121] "CURRENT"  "NEVER"    "NEVER"    "PREVIOUS" "NEVER"    "PREVIOUS"
## [127] "NEVER"    "PREVIOUS" "PREVIOUS" "NEVER"    "PREVIOUS" "PREVIOUS"
## [133] "PREVIOUS" "PREVIOUS" "PREVIOUS" "PREVIOUS" "CURRENT"  "CURRENT" 
## [139] "PREVIOUS" "NEVER"    "PREVIOUS" "PREVIOUS" "NEVER"    "PREVIOUS"
## [145] "PREVIOUS" "PREVIOUS" "PREVIOUS" "PREVIOUS" "PREVIOUS" "NEVER"   
## [151] "PREVIOUS" "PREVIOUS" "NEVER"    "PREVIOUS" "NEVER"
```

```
##        
##         FALSE TRUE
##   FALSE    76   37
##   TRUE     28   14
```

```
## Warning in nominalTrainWorkflow(x = x, y = y, wts = weights, info = trainInfo, :
## There were missing values in resampled performance measures.
```

## Sup. 7: Cross cohort beta-coef comparison

```
##   [1] "PREVIOUS" "PREVIOUS" "PREVIOUS" "PREVIOUS" "PREVIOUS" "PREVIOUS"
##   [7] "NEVER"    "NEVER"    "CURRENT"  "PREVIOUS" "NEVER"    "PREVIOUS"
##  [13] "PREVIOUS" "PREVIOUS" "NEVER"    "PREVIOUS" "PREVIOUS" "PREVIOUS"
##  [19] "PREVIOUS" "PREVIOUS" "PREVIOUS" "PREVIOUS" "PREVIOUS" "PREVIOUS"
##  [25] "PREVIOUS" "CURRENT"  "CURRENT"  "CURRENT"  "PREVIOUS" "PREVIOUS"
##  [31] "PREVIOUS" "NEVER"    "CURRENT"  "NEVER"    "PREVIOUS" "NEVER"   
##  [37] "PREVIOUS" "PREVIOUS" "NEVER"    "PREVIOUS" "NEVER"    "PREVIOUS"
##  [43] "PREVIOUS" "NEVER"    "PREVIOUS" "PREVIOUS" "NEVER"    "PREVIOUS"
##  [49] "NEVER"    "PREVIOUS" "PREVIOUS" "PREVIOUS" "PREVIOUS" "PREVIOUS"
##  [55] "NEVER"    "NEVER"    "NEVER"    "NEVER"    "PREVIOUS" "NEVER"   
##  [61] "NEVER"    "NEVER"    "NEVER"    "PREVIOUS" "CURRENT"  "PREVIOUS"
##  [67] "PREVIOUS" "PREVIOUS" "CURRENT"  "CURRENT"  "PREVIOUS" "NEVER"   
##  [73] "PREVIOUS" "PREVIOUS" "PREVIOUS" "NEVER"    "PREVIOUS" "NEVER"   
##  [79] "NEVER"    "PREVIOUS" "CURRENT"  "PREVIOUS" "PREVIOUS" "NEVER"   
##  [85] "NEVER"    "CURRENT"  "NEVER"    "PREVIOUS" "PREVIOUS" "NEVER"   
##  [91] "NEVER"    "NEVER"    "NEVER"    "NEVER"    "PREVIOUS" "NEVER"   
##  [97] "PREVIOUS" "CURRENT"  "NEVER"    "PREVIOUS" "PREVIOUS" "PREVIOUS"
## [103] "PREVIOUS" "PREVIOUS" "PREVIOUS" "PREVIOUS" "NEVER"    "PREVIOUS"
## [109] "PREVIOUS" "NEVER"    "PREVIOUS" "NEVER"    "PREVIOUS" "PREVIOUS"
## [115] "CURRENT"  "NEVER"    "PREVIOUS" "NEVER"    "PREVIOUS" "PREVIOUS"
## [121] "CURRENT"  "NEVER"    "NEVER"    "PREVIOUS" "NEVER"    "PREVIOUS"
## [127] "NEVER"    "PREVIOUS" "PREVIOUS" "NEVER"    "PREVIOUS" "PREVIOUS"
## [133] "PREVIOUS" "PREVIOUS" "PREVIOUS" "PREVIOUS" "CURRENT"  "CURRENT" 
## [139] "PREVIOUS" "NEVER"    "PREVIOUS" "PREVIOUS" "NEVER"    "PREVIOUS"
## [145] "PREVIOUS" "PREVIOUS" "PREVIOUS" "PREVIOUS" "PREVIOUS" "NEVER"   
## [151] "PREVIOUS" "PREVIOUS" "NEVER"    "PREVIOUS" "NEVER"
```
